# The Natural Redox Cofactor Pyrroloquinoline Quinone (PQQ) Enables Photocatalytic Radical Cyclizations

**DOI:** 10.1002/anie.202505431

**Published:** 2025-07-30

**Authors:** Srishti B. Bahukhandi, Andreas S. Klein, Ghulam Mustafa, Maria Weyh, Alexandra Walter, Erling Thyrhaug, Jürgen Hauer, Golo Storch, Cathleen Zeymer

**Affiliations:** ^1^ Center for Functional Protein Assemblies & Department of Bioscience, TUM School of Natural Sciences Technical University of Munich (TUM) 85748 Garching Germany; ^2^ Department of Chemistry, TUM School of Natural Sciences Technical University of Munich (TUM) 85748 Garching Germany; ^3^ TUM Catalysis Research Center Technical University of Munich (TUM) 85748 Garching Germany

**Keywords:** Biocatalysis, Photoenzyme, Photoredox catalysis, Pyrroloquinoline quinone, Radical reactions

## Abstract

Photoenzymatic catalysis facilitates stereoselective new‐to‐nature chemistry under mild conditions. In addition to the rational design of artificial photoenzymes, naturally occurring redox enzymes can be repurposed to promote photoredox catalysis in the chiral protein environment. Here, we show that enzymes utilizing the pyrroloquinoline quinone (PQQ) cofactor expand the toolbox of photobiocatalysis. PQQ absorbs visible light and is capable of single‐electron transfer. It thus exhibits mechanistic similarities to flavin cofactors, which are widely used for photoenzymatic approaches. First, we established the trimethyl ester PQQMe_3_ as a stand‐alone photoredox catalyst in pure organic solvent. Upon excitation, PQQMe_3_ enables the redox‐neutral radical cyclization of an *N*‐(bromoalkyl)‐substituted indole. We then tested a panel of PQQ‐dependent sugar and alcohol dehydrogenases for photoenzymatic catalysis in aqueous buffer, focusing on a redox‐neutral radical reaction to form oxindoles. Under optimized reaction conditions, we obtained a 69% yield and an 82:18 enantiomeric ratio. Our work thus demonstrates that PQQ enzymes are capable of stereoselective photoredox catalysis. Future enzyme engineering efforts based on computational modeling and directed evolution will fully unlock their synthetic potential.

## Graphical abstract

Natural alcohol and sugar dehydrogenases that utilize the redox cofactor pyrroloquinoline quinone (PQQ) can be repurposed for enantioselective photoredox catalysis. Upon blue‐light irradiation, redox‐neutral radical cyclizations are catalyzed. This work adds a new class of enzymes to the toolbox of photobiocatalysis.

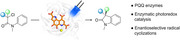

Photoenzymes catalyze light‐driven reactions in the chiral environment of a protein. They thus combine the versatile mechanistic opportunities of substrate activation based on photoexcitation with excellent stereocontrol enabled by specific interactions that stabilize reactive intermediates.^[^ ^1^ ^]^ While only a handful of such enzymes occur in nature,^[^ ^2^ ^]^ protein engineering facilitates the development of artificial photoenzymes,^[^ ^3^, ^4^ ^]^ which represent an important pillar in modern biocatalysis.^[^ ^5^ ^]^ Synthetic photosensitizers can be integrated into protein scaffolds by chemical modification or genetic code expansion. For instance, amino acids with benzophenone or thioxanthone side chains are genetically encodable and have been used to promote stereoselective [2+2] photo‐cycloadditions.^[^ ^6^, ^7^, ^8^ ^]^ Metal‐based photosensitizers can also be incorporated, even by direct coordination of photoredox‐active metal ions inside of proteins, as recently shown by engineering a cerium photoredox enzyme based on a lanthanide‐binding de novo protein.^[^ ^9^ ^]^ Other approaches elegantly exploit the direct excitation of protein‐bound catalytic intermediates^[^ ^10^ ^]^ or use naturally occurring fluorescent proteins^[^ ^11^ ^]^ as a starting point for photoenzyme design.

In contrast to these rational design approaches, natural redox enzymes with flavin cofactors, such as flavin mononucleotide (FMN) and flavin adenine dinucleotide (FAD), have been repurposed successfully for new‐to‐nature photoredox catalysis.^[^ ^12^ ^]^ Upon excitation, both the oxidized quinone state and the reduced hydroquinone state of the cofactors are capable of photoinduced single electron transfer (SET) from/to a substrate molecule. This generates radical intermediates in an oxidative or reductive fashion, often via substrate decarboxylation or dehalogenation, respectively. Examples include stereoselective radical cyclizations^[^ ^13^, ^14^, ^15^ ^]^ as well as intermolecular C─C bond‐forming reactions.^[^ ^16^, ^17^, ^18^ ^]^ While oxidized flavin can be directly excited, the formation of electron‐donor‐acceptor (EDA) complexes is crucial in the reductive cases.^[^ ^19^ ^]^ Furthermore, flavoenzymes have been reported to catalyze redox‐neutral radical cyclizations upon photocatalytic formation of the reactive flavin semiquinone radical state.^[^ ^20^ ^]^ Based on mechanistic analogy to flavin, we hypothesized that pyrroloquinoline quinone (PQQ), another natural redox cofactor previously unexplored in the context of photoenzymatic catalysis, will be suitable to promote light‐driven radical chemistry.

PQQ is a bright red heterocyclic quinone derived from a ribosomally synthesized peptide in a complex biosynthetic pathway.^[^ ^21^ ^]^ It serves as a redox cofactor in bacterial alcohol and sugar dehydrogenases, which adopt a characteristic β‐propeller fold and are typically exported to the periplasm (Figure 1a).^[^ ^22^, ^23^ ^]^ In the active site, the PQQ cofactor usually binds with nanomolar to picomolar affinity^[^ ^24^, ^25^ ^]^ and is activated by a Lewis‐acidic metal ion, either Ca(II) or lanthanide(III).^[^ ^26^, ^27^, ^28^ ^]^ Substrate oxidation is proposed to occur via hydride transfer forming the reduced hydroquinone PQQH_2_. Cofactor regeneration, however, proceeds via the radical semiquinone state PQQ^●−^ upon two sequential SET steps to a cytochrome‐type redox partner.^[^ ^29^ ^]^ Electrochemistry and photophysics of synthetic PQQ derivatives have been studied previously, reporting a triplet excited state one‐electron redox potential of *E*_1/2_^*^ = 1.59 V versus SCE for the trimethyl ester PQQMe_3_.^[^ ^30^, ^31^ ^]^ However, the application of PQQ or PQQ enzymes in photoredox catalysis has not been considered to date. We thus selected a set of redox‐neutral radical cyclizations as our test bed and first established PQQMe_3_ as a stand‐alone photoredox catalyst in organic solvent before moving to stereoselective photoenzymatic reactions in aqueous buffer (Figure 1b).

**Figure 1:**
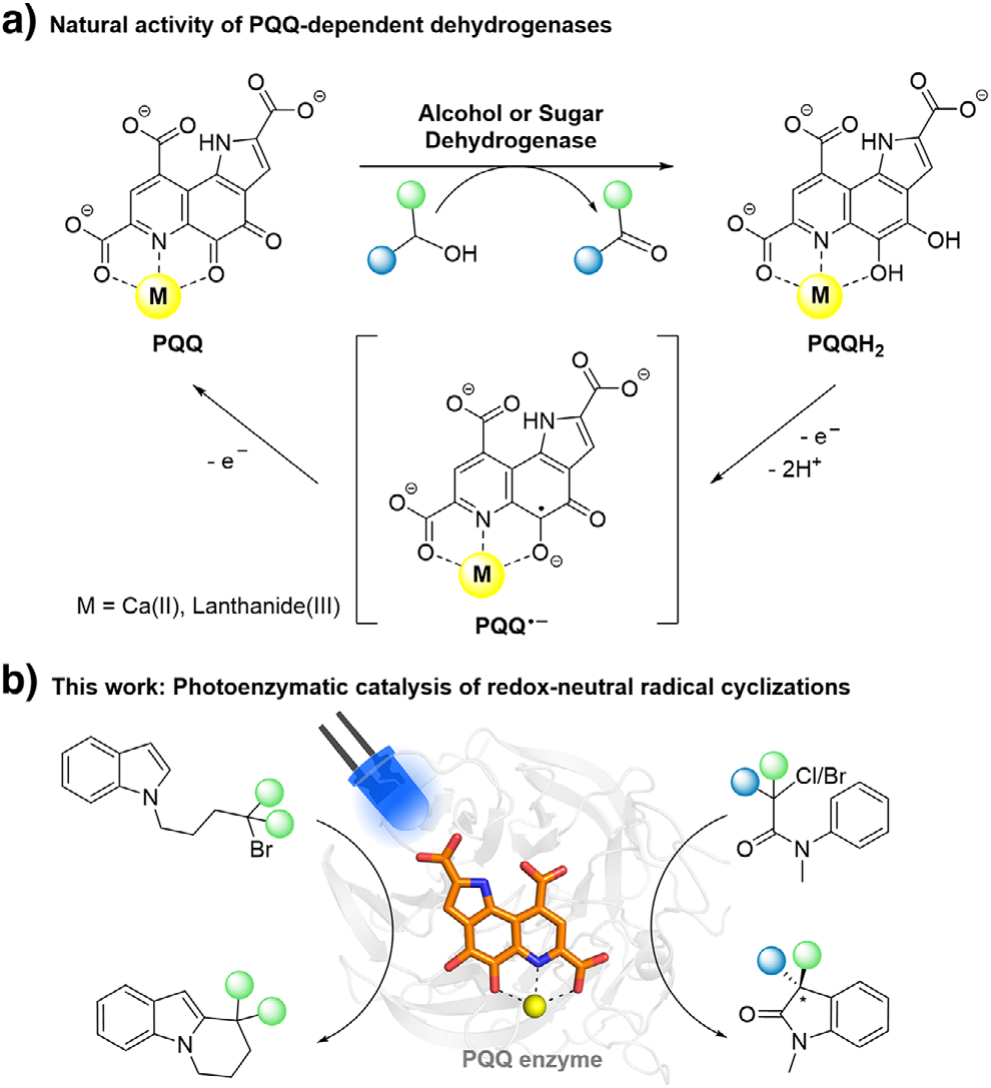
PQQ‐dependent enzyme catalysis. a) Oxidation states of the PQQ cofactor within the native catalytic cycle of bacterial alcohol and sugar dehydrogenases. b) The PQQ cofactor as a photoredox catalyst for new‐to‐nature radical chemistry.

We started by synthesizing PQQMe_3_ from the PQQ sodium salt, which is commercially available as a nutritional supplement. The obtained UV–vis absorption, fluorescence, and cyclic voltammetry data of PQQMe_3_ in MeCN and DCM were in agreement with the available literature (Figures 2a and S4, S5).^[^ ^30^, ^31^ ^]^ We then selected the redox‐neutral radical cyclization of *N*‐(bromoalkyl)‐substituted indole **1** to test for photoredox catalysis by PQQMe_3_. When performing the reaction under visible‐light irradiation in MeCN in the presence of NEt_3_ as the sacrificial electron donor, we obtained 80% yield of cyclized product **2** and high chemoselectivity over the linear dehalogenated product **3**. Controls in the absence of light, NEt_3_, or PQQMe_3_ gave no or a significantly reduced yield (Figure 2b). It is important to note that the reaction needs to be performed under inert atmosphere; in the presence of air, no reaction was observed. For reaction optimization, different wavelengths, solvents, sacrificial electron donors, catalyst loading, and reaction times were tested (Tables S1–S5). This cyclization has been reported previously using [Ru(bpy)_3_]Cl_2_ and [Ni(Mabiq)]OTf as photoredox catalysts.^[^ ^32^, ^33^ ^]^ We thus propose a similar mechanism (Figure 2d), in which PQQMe_3_ is excited and reductively quenched by NEt_3_. The semiquinone radical PQQMe_3_^●−^ then undergoes SET to reductively cleave the C─Br bond, generating a carbon‐centered tertiary radical, which reacts with the indole C2 position to form the cyclized product. We performed light on/off kinetics showing that the reaction only proceeds under constant irradiation (Figure 2c). In further mechanistic experiments, we ran the reaction in the presence of radical trapping agents and triplet quenchers. The addition of 2,2,6,6‐tetramethylpiperidinyloxyl (TEMPO) and 1,1‐diphenylethylene (DPE) reduced the product yield to trace amounts and the respective adducts with the radical intermediate were identified by mass spectrometry (Figures 2b and FS8). Triplet quenchers dibutylhydroxytoluene (BHT) and pyridazine also abolished the reaction, which supports the previously described finding that PQQMe_3_ preferentially populates the triplet excited state (Figure 2b).^[^ ^31^ ^]^ This is also in agreement with Stern–Volmer experiments showing no significant changes in singlet excited state lifetimes in the presence of NEt_3_ (Figures S9, S10), while PQQMe_3_ absorption spectra exhibit substantial changes in photolysis experiments with NEt_3_ (Figure 2a). Overall, our experiments show that the trimethyl ester of the natural PQQ cofactor can be employed as a stand‐alone photoredox catalyst in organic solvent.

**Figure 2:**
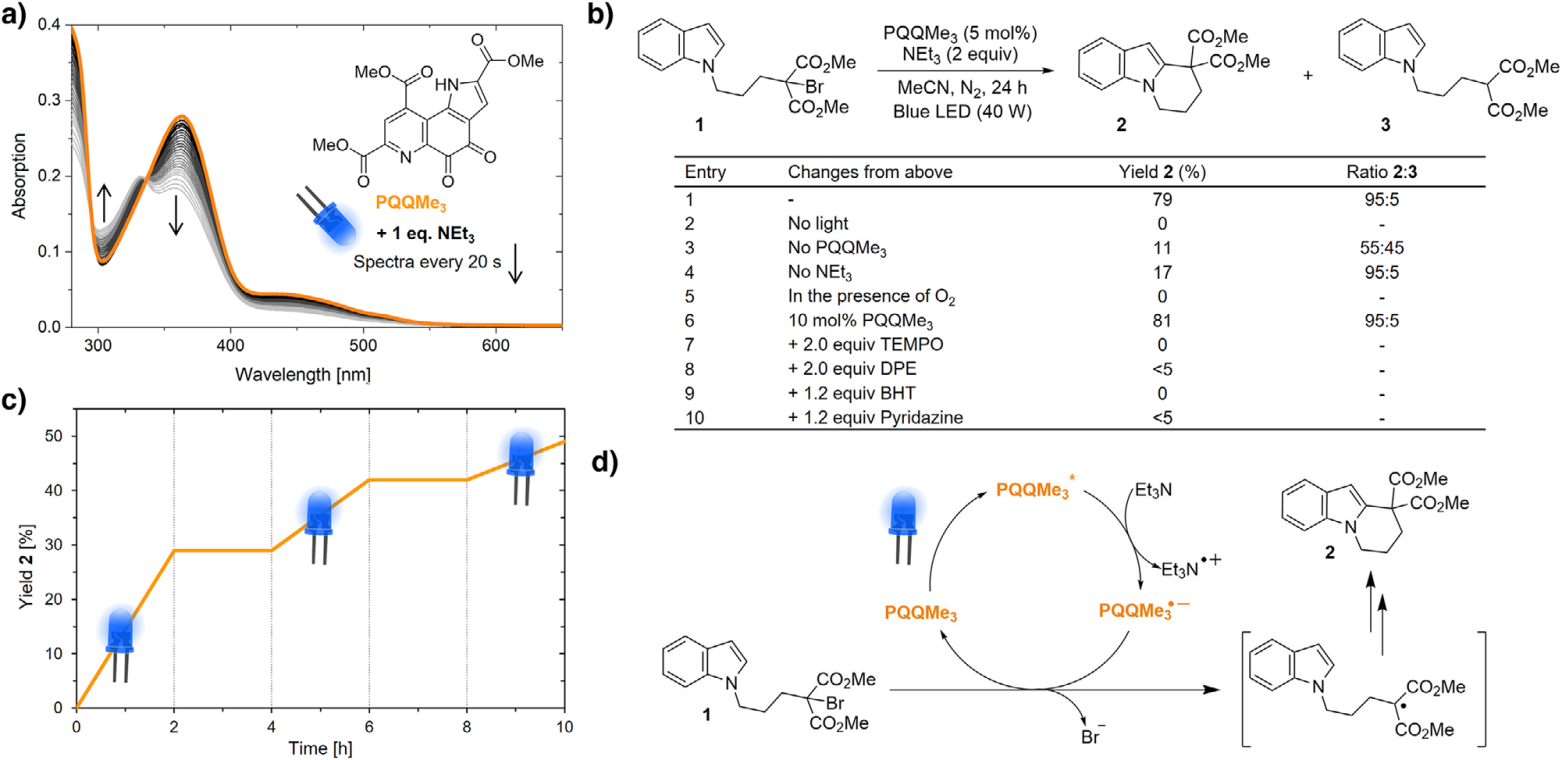
PQQMe_3_ as a stand‐alone photoredox catalyst in organic solvent. a) Structure and absorption spectrum of PQQMe_3_ in orange. Spectra recorded in a photolysis setup upon addition of 1 equiv. NEt_3_ and irradiation in 20 s intervals are shown in shades of gray. b) Redox‐neutral radical cyclization of *N*‐(bromoalkyl)‐substituted indole **1** catalyzed by PQQMe_3_ upon visible‐light irradiation. The reported yield of **2** is an isolated yield, while the ratio **2:3** has been determined by ^1^H‐NMR. c) Light on/off kinetics. d) Proposed catalytic cycle.

Next, we asked whether PQQ‐dependent dehydrogenases, just like flavoenzymes, could facilitate enzymatic photoredox catalysis in aqueous buffer. To that end, we selected a panel of bacterial PQQ enzymes: three Ca(II)‐dependent aldose sugar dehydrogenases from *Escherichia coli*, *Thermus thermophilus*, and *Acinetobacter calcoaceticus*, respectively, and one lanthanide(III)‐dependent alcohol dehydrogenase from *Pseudomonas putida* (all sequences available in the Supporting Information).^[^ ^22^, ^23^, ^34^, ^35^ ^]^ While all three sugar dehydrogenases have a relatively large substrate binding pocket (Figure 3a), the natural substrate of the alcohol dehydrogenase is ethanol, which binds in a small and buried active site. However, this enzyme has been previously engineered to accept larger substrates and we thus decided to work with the reported double mutant (F412V/W561A) of PedH from *P. putida*.^[^ ^35^ ^]^ We recombinantly expressed all proteins in *E. coli* and reconstituted the enzymes by adding PQQ and the respective metal ions in vitro after affinity purification (Figure 3b). To verify their natural enzymatic activity, which is the oxidation of glucose and ethanol, respectively, we performed a coupled colorimetric assay (Figure S2). Furthermore, we obtained UV–vis absorption spectra, fluorescence spectra, and lifetimes of PQQ enzymes in comparison to free PQQ in aqueous solution. Interestingly, PQQ's absorption spectrum shows a distinct change when bound to the protein, which is in agreement with the observed color change from red to yellow during reconstitution (Figure 3c). Both the free cofactor as well as the reconstituted enzyme show fluorescence with an emission maximum at 500 nm and a lifetime of *τ* = 1.2 ns in aqueous buffer (Figures S6, S7). For PQQ bound to the enzyme, a weak second emission band at around 600 nm was observed when exciting at 450 nm. Due to different protonation states and adduct formation at the quinone, for instance with water or other nucleophiles,^[^ ^36^ ^]^ several PQQ species may be present in parallel.

**Figure 3:**
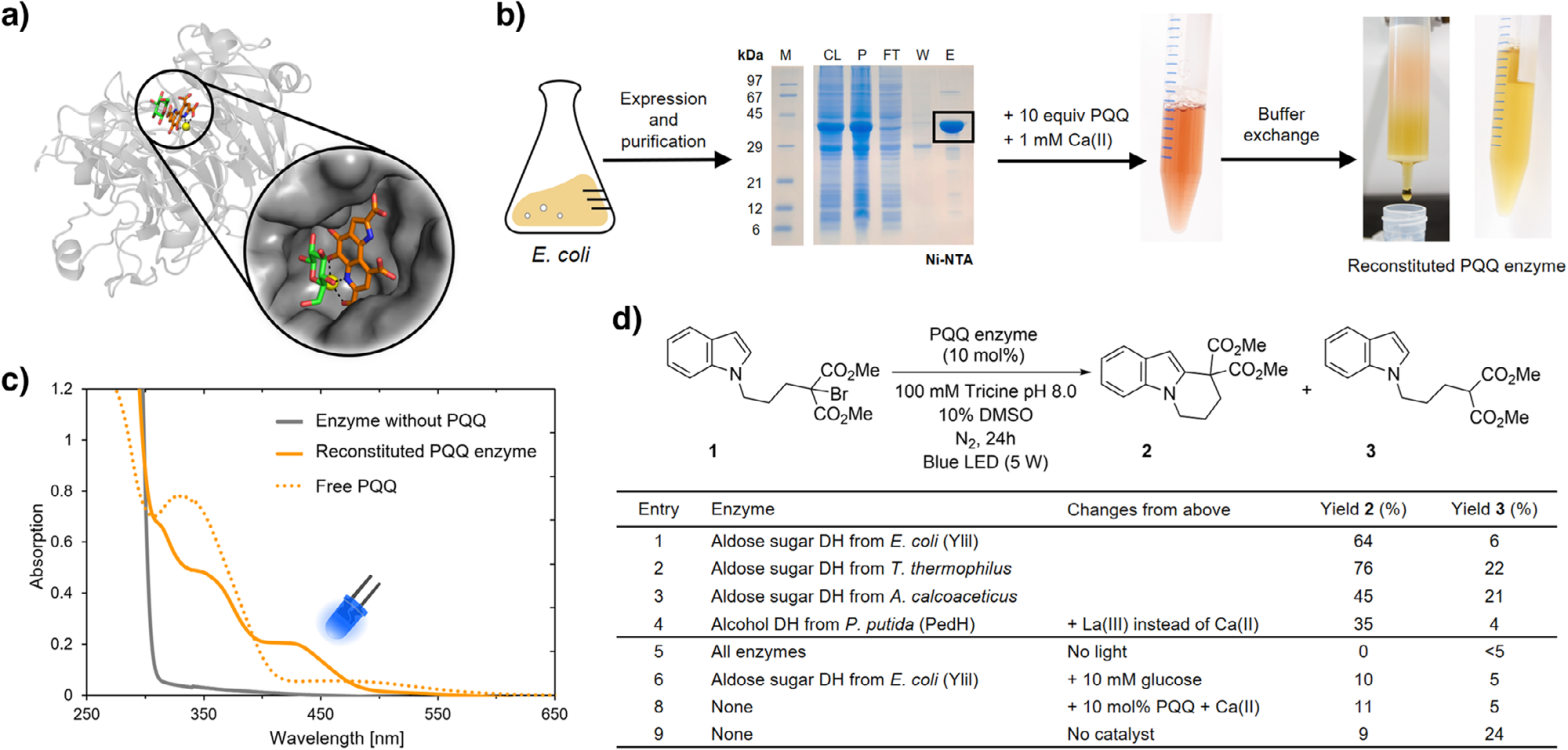
Enzymatic photoredox catalysis with PQQ enzymes. a) Crystal structure of a bacterial sugar aldose dehydrogenase (DH) in complex with PQQ, Ca(II), and glucose (PDB: 1CQ1, PQQ in orange, glucose in green, and Ca(II) in yellow). b) Recombinant expression, purification, and reconstitution of PQQ enzymes. c) Absorption spectra of purified protein without cofactor, reconstituted PQQ enzyme, and free PQQ in aqueous buffer, shown here exemplarily for YliI from *E. coli*. d) Redox‐neutral radical cyclization of *N*‐(bromoalkyl)‐substituted indole **1** catalyzed by PQQ enzymes upon visible‐light irradiation.

Enzymatic photoredox catalysis was tested using the same reaction as above for PQQMe_3_. We performed the reaction in 100 mM tricine buffer at pH 8.0, where the zwitterionic amino acid not only served as the buffer component but also replaced NEt_3_ as the sacrificial electron donor (supported by photolysis data shown in Figure S20). Upon blue‐light irradiation, we obtained up to 76% yield of cyclized product **2**, with best results for the aldose sugar dehydrogenases from *E. coli* and *T. thermophilus* (Figure 3d). Controls without light or enzyme showed no or significantly lower yields, respectively, and also the enzymatic reactions were strongly sensitive to oxygen and small‐molecule triplet quenchers (Table S14). When adding the native substrate glucose to fully reduce the enzyme, the cyclization activity dropped to the level of background, indicating that the oxidized PQQ state is photocatalytically essential (Figure 3d). To further exclude a photocatalytic contribution of the reduced PQQH_2_ state, we performed Stern–Volmer experiments (Figures S13–S19) and investigated the formation of a potential charge transfer complex with the substrate (Figures S21, S22). However, all results suggest no productive interaction between the substrate and the reduced enzyme. We thus conclude that PQQ enzymes are capable of promoting this light‐driven radical reaction upon initial excitation of the oxidized cofactor rather than the reduced one.

With this proof‐of‐concept in hand, we aimed to explore enantioselective redox‐neutral radical cyclizations. To that end, we shifted to a different class of substrates previously reported in the context of flavin‐dependent ene reductases.^[^ ^20^ ^]^ Importantly, these α‐chloroamides gave no background reaction upon irradiation without enzyme. Using substrate **4**, we obtained oxindole product **5** with 69% yield and 82:18 *e.r*. (Figure 4a), confirming that PQQ enzymes indeed enable stereocontrol in radical cyclizations. The different enzymes showed variable yield and enantioselectivity, which is certainly due to their different substrate binding pockets, but also due to variable stability under the reaction conditions. A time course measurement revealed that cyclized product **5** exhibits a slight drop in *e.r*. over reaction time, from 88:12 after 4 h to 70:30 after 48 h. Notably, the linear dehalogenated side product **6** is initially also formed with high enantioselectivity (up to 95:5 *e.r*. after 2 h of reaction time) but ends up almost completely racemic after 48 h (Table S11). This is likely due to subsequent nonenzymatic racemization, as the stereocenter of **6** carries an acidic proton. Still, our observation suggests a stereoselective hydrogen atom transfer (HAT) step, involving either the protonated semiquinone radical PQQH^●^ or a close‐by amino acid residue. As also the substrate (**4**) is chiral, we could investigate if the enzyme confers stereocontrol also in the initial radical formation step. However, here we did not observe any kinetic resolution of the racemic starting material (Table S11). In the final step of the mechanism to form cyclized product **5**, the semiquinone radical is formally regenerated and could immediately enter the next catalytic cycle. However, light on/off kinetics obtained for the enzymatic reaction show no conversion in the dark (Figure S11). Furthermore, we preirradiated the PQQ‐bound enzyme for 30 min to form the PQQ^●−^ state, then added substrate and performed the reaction in the dark. Here, we obtained a very small amount of product (Figure S12). We thus propose, as also suggested for the flavoenzyme‐catalyzed case,^[^ ^20^ ^]^ that the semiquinone radical must thus be constantly regenerated in a photocatalytic fashion (Figure 4b).

**Figure 4:**
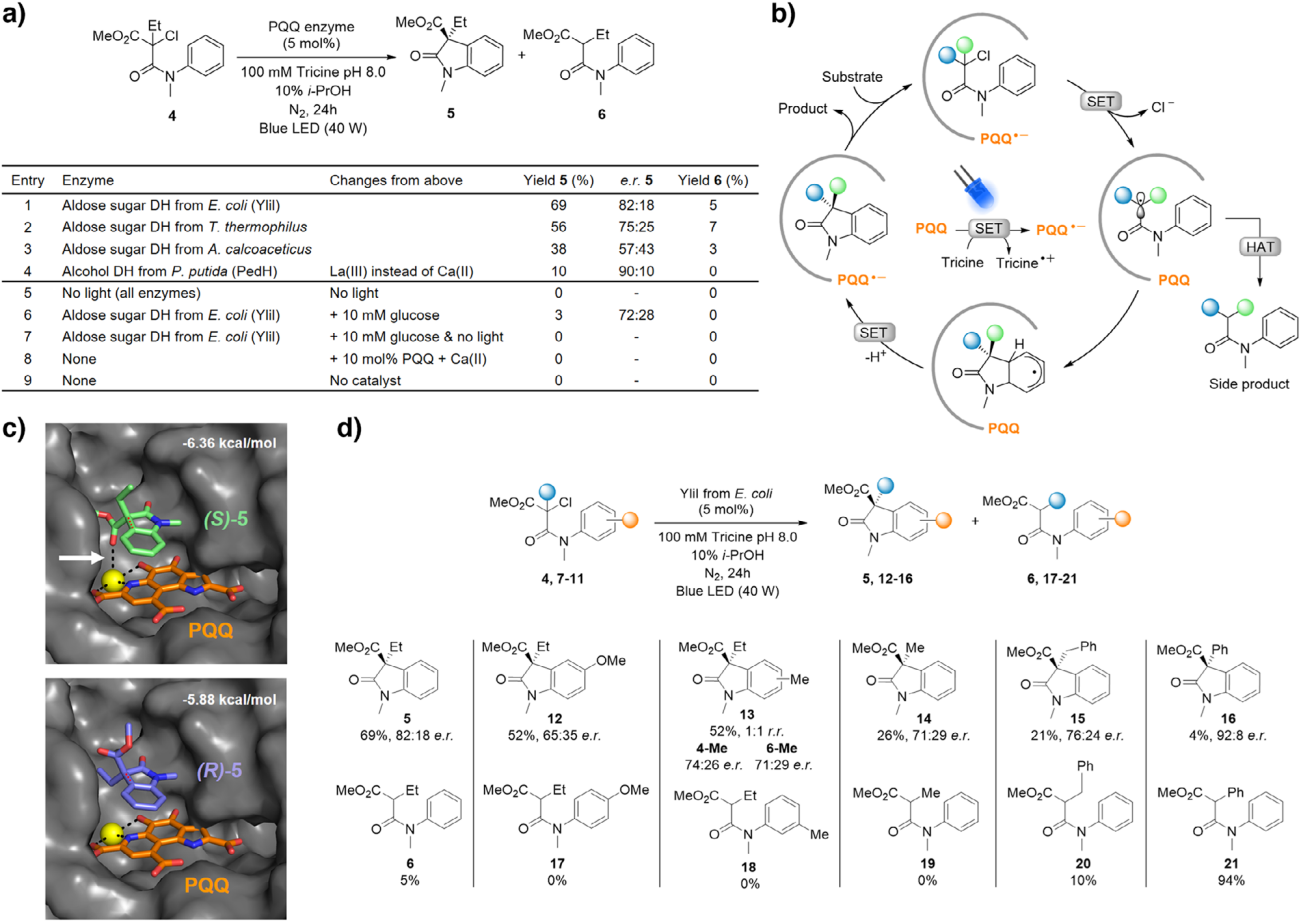
Stereoselective cyclizations. a) Redox‐neutral radical cyclization to form oxindole product **5** catalyzed by PQQ enzymes upon visible‐light irradiation. b) Proposed catalytic cycle. c) Computational model of product enantiomers binding in the active site of YliI (PDB: 2G8S, PQQ in orange, oxindole product in light green and purple, and Ca(II) in yellow). The binding energies have been generated by the deep learning‐based docking tool GNINA.^[^ ^37^ ^]^ d) Substrate scope.

To gain insights into how the protein environment in the best‐performing enzyme YliI from *E. coli* discriminates between both product enantiomers, we performed computational modeling. Binding of both (*S*)‐**5** and (*R*)‐**5** to the active site in the presence of Ca(II) and PQQ was assessed using the deep learning‐based structure prediction and docking tools Boltz^[^ ^38^ ^]^ and GNINA,^[^ ^37^ ^]^ respectively. The lowest energy docking poses show both enantiomers in essentially the same position above the PQQ cofactor. However, the ester carbonyl group of (*S*)‐**5** can interact with the Ca(II) ion and is thus stabilized compared to (*R*)‐**5** (Figure 4c). It can be assumed that this energy contribution is also decisive in the transition state of the cyclization, thus favoring the formation of (*S*)‐**5**, which is the enantiomer we observe experimentally.

Finally, we investigated a scope of α‐chloroamide substrates (Figure 4d). We hypothesized that larger groups at the quaternary carbon, such as benzyl and phenyl in substrates **10** and **11**, may be supported by the open active site architecture. Indeed, the respective products were formed with enantiocontrol but at low yields. This may be due to subtle changes in protein–substrate interactions that can influence the binding pose, distance to the cofactor, or dissociation kinetics, and thereby modify the reactivity. Interestingly, the phenyl substituent inverts the ratio between cyclized and linear dehalogenated product. Here, a benzylic radical intermediate is generated, which is significantly more stable and long‐lived than in all other cases. It could thus diffuse out of the active site and quench unspecifically, which seems to be favored over the cyclization. To support this hypothesis, we also analyzed the *e.r*. of product **21** over the course of the reaction. In contrast to the equivalent product **6**, where we observed initial stereoselectivity at short reaction times, product **21** is formed completely racemically (Table S11).

In conclusion, we demonstrate that the natural redox cofactor PQQ facilitates photocatalytic radical cyclizations upon visible‐light irradiation. The trimethyl ester PQQMe_3_ can be utilized as a stand‐alone photoredox catalyst in organic solvent. With its long‐lived and strongly oxidizing triplet state,^[^ ^31^ ^]^ it may be utilized in various other photoredox reactions in the future. Its availability as a nutritional supplement also makes it an inexpensive alternative compared to most metal‐based photoredox catalysts. Most importantly, we show that PQQ‐dependent dehydrogenases represent an attractive addition to the toolbox of stereoselective photoenzymatic catalysis. The synthetic potential of this enzyme class likely extends beyond the redox‐neutral radical cyclizations reported here, offering exciting opportunities for future exploration.

## Conflict of Interests

The authors declare no conflict of interest.

## Supporting information



Supporting Information

## Data Availability

All raw data files associated with this study are openly available in the repository *mediaTUM* via https://doi.org/10.14459/2025mp1782387.
