# Supplementary material for: The Natural Redox Cofactor Pyrroloquinoline Quinone (PQQ) Enables Photocatalytic Radical Cyclizations
Source: Angew Chem Int Ed Engl. 2025 Jul 30;64(40):e202505431. doi: 10.1002/anie.202505431 (PMC12462760; doi:10.1002/anie.202505431)
Supplement: Supplementary file 1 — Supporting Information [file ANIE-64-e202505431-s001.pdf]

Supporting Information

The Natural Redox Cofactor Pyrroloquinoline Quinone (PQQ) Enables Photocatalytic Radical Cyclizations

Srishti B. Bahukhandi[1], Andreas S. Klein[1], Ghulam Mustafa[1], Maria Weyh[1], Alexandra Walter[2], Erling Thyrhaug[2,3], Jürgen Hauer[2,3], Golo Storch[2,3], Cathleen Zeymer[1,3,*]

[1] Center for Functional Protein Assemblies & Department of Bioscience, TUM School of Natural Sciences, Technical University of Munich (TUM), 85748 Garching, Germany

[2] Department of Chemistry, TUM School of Natural Sciences, Technical University of Munich (TUM), 85748 Garching, Germany

[3] TUM Catalysis Research Center, Technical University of Munich (TUM), 85748 Garching, Germany

*Corresponding author: Cathleen Zeymer, cathleen.zeymer@tum.de

Table of contents

S1	General methods	Page 4
S2	Molecular cloning, recombinant expression, protein purification, and enzyme reconstitution	Page 7
S3	Native activity assays of PQQ enzymes	Page 13
S4	Protein mass spectrometry	Page 14
S5	Chemical syntheses	Page 15
S6	Cyclic voltammetry of $PQQMe_3$	Page 32
S7	Fluorescence spectroscopy	Page 34
S8	Photocatalytic cyclizations with $PQQMe_3$ in organic solvent	Page 37
S9	Mechanistic investigations with $PQQMe_3$	Page 40
S10	Photoenzymatic cyclizations with PQQ enzymes	Page 43
S11	Mechanistic investigations with PQQ enzymes	Page 47
S12	Computational modeling and docking	Page 54
S13	HPLC data	Page 55
S14	NMR spectra	Page 70
S15	References	Page 92

List of Figures

Figure S1	The 6-vial photoreactor setup	Page 5
Figure S2	DCPIP assay for the determination of native enzymatic activity of all PQQ enzymes	Page 13
Figure S3	Mass spectrometry analysis of PQQ enzymes	Page 14
Figure S4	Cyclic voltammetry data of $PQQMe_3$	Page 32
Figure S5	Absorption and fluorescence spectra of $PQQMe_3$ in DCM	Page 34
Figure S6	Absorption and fluorescence spectra of free PQQ	Page 35
Figure S7	Absorption and fluorescence spectra of PQQ bound to the enzyme	Page 35
Figure S8	Radical intermediate trapping with TEMPO and DPE detected by LC-MS	Page 40
Figure S9	Fluorescence quenching of $PQQMe_3$ (left) and Stern-Volmer plot (right)	Page 41
Figure S10	Changes in fluorescence lifetimes of $PQQMe_3$ upon addition of NEt_3	Page 42
Figure S11	Light on/off experiment with aldose sugar DH from *E. coli* (YliI)	Page 47
Figure S12	Photoreduction of aldose sugar DH from *E. coli* (YliI) and subsequent dark reaction	Page 47
Figure S13	Normalized emission spectra of aldose sugar DH from *E. coli* (YliI) in tricine buffer	Page 49
Figure S14	Comparison of normalized absorption and excitation spectra of YliI	Page 49
Figure S15	Comparison of YliI fluorescence after 450 nm excitation in reducing (tricine) and non-reducing (KPi) buffer solution	Page 50
Figure S16	Fluorescence lifetimes of YliI in phosphate buffer after 375 nm excitation (left) and 450 nm excitation (right)	Page 50
Figure S17	Fluorescence lifetimes of YliI in tricine buffer after 375 nm excitation (left) and 450 nm excitation (right)	Page 51
Figure S18	Fluorescence spectra of reduced YliI in the presence and absence of substrate **4**	Page 51
Figure S19	Fluorescence lifetimes of reduced YliI in the presence and absence of substrate **4**	Page 52
Figure S20	Photolysis experiment with aldose sugar DH from *E. coli* (YliI) in tricine buffer	Page 52
Figure S21	Absorption spectra of aldose sugar DH from *E. coli* (YliI) in the absence and presence of substrate **1**	Page 53
Figure S22	Absorption spectra of aldose sugar DH from *E. coli* (YliI) in the absence and presence of substrate **4**	Page 53

List of Tables

**$PQQMe_3$ as stand-alone photoredox catalyst in organic solvent**		
Table S1	Solvent screening	Page 37
Table S2	Reaction time screening	Page 38
Table S3	Catalyst loading screening	Page 38
Table S4	Sacrificial donor screening	Page 38
Table S5	Wavelength screening	Page 39
Table S6	Radical trapping with TEMPO and DPE	Page 40
Table S7	Triplet quenching with BHT and pyridazine	Page 41
**Enzymatic photoredox catalysis with PQQ enzymes enabling stereoselective redox-neutral cyclizations of α-chloro amides**		
Table S8	Evaluation of different wavelengths	Page 44
Table S9	Evaluation of organic co-solvents	Page 44
Table S10	Evaluation of substrate concentration	Page 45
Table S11	Evaluation of reaction time	Page 45
Table S12	Evaluation of pH	Page 46
Table S13	Enzyme screening under optimized conditions	Page 46
Table S14	Triplet quenching with BHT and pyridazine	Page 48

S1 General methods

Unless otherwise stated, all commercially available compounds were used as provided without further purification. Chemicals used in this manuscript were purchased from Sigma Aldrich, Alfa Aesar, BLDpharm, TCI and abcr. Solvents used in reactions were p.A. grade. Solvents for chromatography were technical grade and distilled prior to use. Analytical thin-layer chromatography (TLC) was performed on pre-coated plastic sheets (Polygram SIL G/UV254, Macherey–Nagel, Düren, Germany), visualized by irradiation with a UV lamp. Column chromatography was performed using silica gel 60 (particle size 0.040–0.063 mm, 230–240 mesh, Macherey–Nagel, Düren, Germany) or via a Biotage system using Sfär Silica Duo 60 µm (Biotage, Uppsala, Sweden). Solvent mixtures are understood as volume/volume. Room temperature is specified as 21-23 °C. All air sensitive reactions were conducted under N_2 using standard Schlenk techniques.

NMR spectroscopy: ^{1}H and ^{13}C NMR spectra were recorded at room temperature on a Bruker Avance III HD 400 or a Bruker Avance III HD 500 nuclear magnetic resonance spectrometer (Bruker, Billerica, USA) at ambient temperature in $CDCl_3$, DMSO-d_6 or C_6D_6 at 400/101 MHz or 500/126 MHz, respectively. The chemical shifts are given in ppm relative to tetramethylsilane [^{1}H: δ($SiMe_4$) = 0.00 ppm] as an internal standard or relative to the solvent [^{1}H: δ($CDCl_3$) = 7.26 ppm; ^{13}C: δ($CDCl_3$) = 77.16 ppm, ^{1}H: δ(DMSO-d_6) = 2.50 ppm; ^{13}C: δ(DMSO-d_6) = 39.52 ppm], or ^{1}H: δ(C_6D_6) = 7.16 ppm]. Signals were assigned by means of ^{1}H, ^{13}C experiments; splitting patterns are given as singlet (s), doublet (d), triplet (t), quartett (q), doublet of doublet (dd), doublet of doublet of doublet (ddd), multiplet (m) and broad singlet (brs). Coupling constants (J) are reported in Hz.

UV/Vis absorption spectroscopy: Absorption spectra were recorded on a Jasco V-750 photometer (JASCO Deutschland GmbH, Pfungstadt, Germany) at room temperature or otherwise specified (data interval: 1 nm; bandwidth: 0.2 nm; response: 0.24 s, path length: 1 cm). Background absorption was corrected by recording a blank spectrum in advance.

The 6-vial photoreactor setup: All LEDs were purchased at *Avonec* (*Avonec*, Wesel, Germany) as high-power LEDs soldered on starboards (455–460 nm 5 W, 410–420 nm 3 W, and 480–485 nm 3 W). The LEDs were glued using Keratherm Bond 100 RT thermal adhesive (KERAFOL, Eschenbach in der Oberpfalz, Germany) onto Fischer Elektronik SK 42 heat sinks (100x160x25 mm, aluminum, 0.95K/W; Fischer Elektronik, Lüdenscheid, Germany; Figure S1A). Cooling of the LED heat sink and the six reaction vials (each positioned precisely above one LED) was carried out using a hollow aluminum block (Figure S1B) attached to a Minichiller 280 OLÉ cooling unit (Peter Huber Kältemaschinenbau SE, Offenburg, Germany). The cooling water temperature was set to 18 °C giving a reproducible reaction temperature of approx. 23 °C. The MW LCM-40 LED drivers (MEAN WELL Enterprises Co, New Taipei City, Taiwan) were operated at a constant current of 0.7 A. Importantly, this setup ensured reproducible conditions for all photoenzymatic reactions.

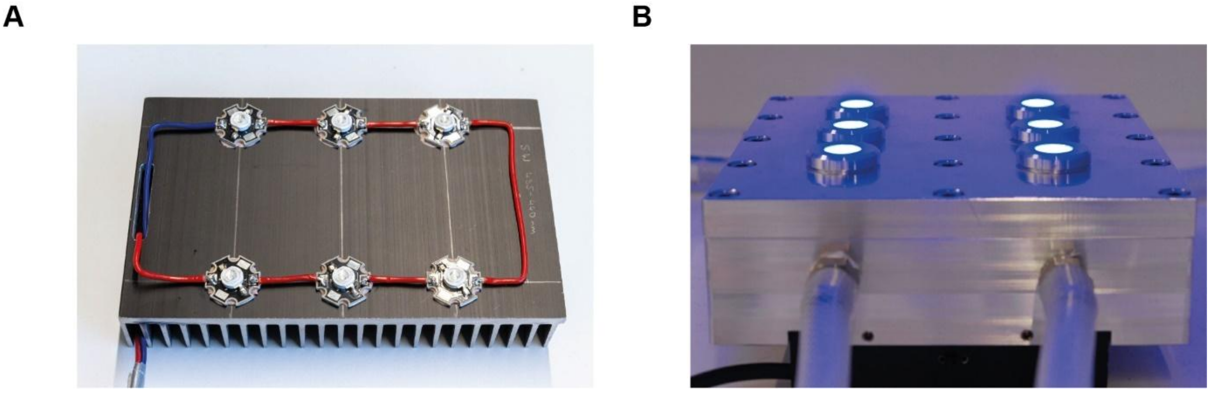

Figure S1: The 6-vial photoreactor setup. ***(A)*** *High power LEDs glued onto the heat sink.* ***(B)*** *The water-cooled aluminum block positions the reaction vials above the LEDs.*

LED Lamps: Kessil lamps used in this study were A160WE TUNA BLUE (40W), PR160L-427 nm (40W), and PR160L-390 nm (40W).

HPLC: HPLC analysis was performed on a Vanquish System (Thermo Fisher Scientific, Waltham, MA, USA) with VF-D11-A diode array detector equipped with a chiral CHIRALCEL® OJ-RH column (150 x 4.6 mm, 5 µm particle size, Daicel Corporation, Osaka, Japan) at room temperature, a chiral CHIRALPAK® IC (4.6x250mm, 5 µm particle size, Daicel Corporation, Osaka, Japan) at room temperature and 40°C, and a CHIRALCEL® OD-RH (150 x 4.6mm, 5 µm particle size, Daicel Corporation, Osaka, Japan) at room temperature. Detection wavelengths were 205 nm and 254 nm, 3D field (190 nm–450 nm).

Liquid chromatography / mass spectrometry (LC-MS): The measurements were performed using an Agilent 1290 Infinity II series UHPLC (Agilent Technologies, Santa Clara, CA, USA) equipped with a diode array detector (G7117C) and a single quadrupole mass detector (G6135B) with an AJS (Agilent Jet Stream) electrospray ion source. Analytes were separated on a Poroshell 120 EC-C18 column (100 x 2.1 mm, 1.9 µm particle size, Agilent Technologies, Santa Clara, CA, USA) at 30 °C. Water + 0.1% (v/v) formic acid and acetonitrile + 0.1% (v/v) formic acid were used as eluents. 2 µL of each sample was injected. Compounds were identified by their UV absorption spectra and their mass to charge ratio (m/z). Detection wavelengths were 254 nm and 3D field (220 nm–450 nm).

Cyclic voltammetry: Electrochemical measurements were carried out with a Metrohm Autolab PGSTAT204 using glassy carbon (diameter = 3 mm) as a working electrode, a platinum wire as a counter electrode and Ag/$AgNO_3$ (0.01 M $AgNO_3$ in electrolyte) separated by a vycor frit as the reference electrode. A five-necked flask was used: One inlet was used to flush the cell with argon, one inlet was used for the addition of the samples, and the remaining inlets were fitted with the counter, working, and reference electrodes. All potentials are given *vs.* SCE. For referencing, ferrocene was used as an internal standard ($E_{1/2}$(Fc^+/Fc) = 74 mV *vs.* Ag/$AgNO_3$) and the potentials were adjusted to the SCE scale (Ag/$AgNO_3$ = 298 mV *vs.* SCE). All cyclic voltammograms (CVs) were measured with a scan rate of 0.5 V s^{-1}, unless otherwise noted. The measurements were carried out using pure acetonitrile (MeCN) as solvent, and tetrabutylammonium hexafluorophosphate ($TBAPF_6$; c = 0.1 M) was used as the electrolyte.

Fluorescence spectroscopy: Fluorescence experiments were performed on an Edinburgh Instruments Spectrofluorometer FS5 equipped with a continuous Xe-source for steady state spectra and a TCSPC laser diode for excited state lifetime measurements. Spectra were recorded in screw cap cuvettes (1 cm pathway). All solutions were handled under N_2 atmosphere to ensure a complete absence of oxygen during the measurements.

S2 Molecular cloning, recombinant expression, protein purification, and enzyme reconstitution

S2.1 Bacterial strains and culture conditions

Cultivation of *Escherichia coli* (*E. coli*) BL21-Gold(DE3), SoluBL21, and DH10B strains was carried out in LB (lysogeny broth) liquid medium (Carl Roth, Karlsruhe, Germany: 10 g/L tryptone, 5 g/L yeast extract, 10 g/L sodium chloride) or on LB agar plates [addition of 1.5% (w/v) agar] at 37 °C. Ampicillin (final concentration of 100 µg/mL) or kanamycin (final concentration of 30 µg/mL) were added as antibiotics to the culture medium. All cultivation media were prepared using distilled water and sterilized by autoclaving.

S2.2 Molecular cloning

Synthetic genes were ordered from *Twist Bioscience* (South San Francisco, CA, USA). pET-29b(+) was used as the expression vector for all PQQ enzymes. All genes were inserted between the NdeI and XhoI restriction sites and contained an N-terminal or C-terminal 6xHis tag.

S2.3 Protein and DNA sequences

Molecular weights (MW) and molar extinction coefficients (ε_{280}) were calculated using the ProtParam tool on the Expasy Server.

Aldose sugar DH from *E. coli* (YliI)
PDB: 2G8S

Molecular weight [Da]: 39763.78
ε_{280} [$M^{-1}cm^{-1}$]: 75400

Protein Sequence

MGHHHHHHAPATVNVEVLQDKLDHPWALAFLPDNHGMLITLRGGELRHWQAGKGLSAPLSGVPDV
WAHGQGGLLDVVLAPDFAQSRRIWLSYSEVGDDGKAGTAVGYGRLSDDLSKVTDFRTVFRQMPKLS
TGNHFGGRLVFDGKGYLFIALGENNQRPTAQDLDKLQGKLVRLTDQGEIPDDNPFIKESGARAEIWSY
GIRNPQGMAMNPWSNALWLNEHGPRGGDEINIPQKGKNYGWPLATWGINYSGFKIPEAKGEIVAGTE
QPVFYWKDSPAVSGMAFYNSDKFPQWQQKLFIGALKDKDVIVMSVNGDKVTEDGRILTDRGQRIRDV
RTGPDGYLYVLTDESSGELLKVSPRN

DNA Sequence

ATGGGCCACCATCATCATCACCATGCTCCTGCAACGGTAAATGTCGAAGTACTGCAAGACAAACT
CGACCATCCCTGGGCACTGGCCTTTTTACCCGATAATCACGGTATGTTAATCACTCTGCGCGGCG
GCGAGTTGCGTCACTGGCAAGCAGGAAAAGGATTATCTGCGCCGCTTTCCGGAGTTCCGGACGT
TTGGGCGCACGGGCAGGGCGGCCTGCTGGACGTGGTTTTAGCGCCTGATTTTGCTCAGTCTCG
CCGCATCTGGTTAAGTTATTCCGAAGTTGGCGATGATGGCAAAGCCGGAACTGCTGTGGGTTATG
GCCGCTTAAGTGATGATCTCTCAAAAGTGACCGACTTCCGCACCGTCTTTCGCCAGATGCCAAAA
CTGTCTACCGGCAACCATTTTGGCGGGCGGCTGGTATTCGACGGTAAAGGTTATCTTTTTATTGCT
CTGGGCGAAAACAATCAGCGCCCGACGGCGCAGGATCTGGATAAATTACAGGGCAAACTGGTGC
GTCTGACCGACCAGGGCGAAATCCCGGATGATAATCCTTTTATAAAGGAATCCGGTGCGCGCGCC
GAGATCTGGTCTTATGGCATTCGTAATCCGCAAGGAATGGCGATGAATCCGTGGAGTAATGCACTG
TGGCTGAATGAACATGGCCCGCGCGGTGGTGATGAAATTAATATCCCGCAAAAAGGCAAAAACTA

CGGCTGGCCGCTGGCAACCTGGGGAATCAACTATTCAGGCTTTAAGATACCGGAAGCGAAAGGG
GAGATCGTCGCCGGGACCGAGCAACCTGTTTTTTACTGGAAAGATTCGCCCGCTGTGAGCGGCA
TGGCCTTCTATAACAGCGATAAATTCCCCCAGTGGCAGCAAAAATTATTTATTGGCGCGCTGAAAG
ATAAAGATGTCATTGTGATGAGCGTCAACGGCGACAAAGTGACAGAAGATGGCCGTATTTTAACG
GACAGAGGGCAGCGAATTCGTGATGTTCGCACTGGACCCGACGGTTATTTATACGTTCTCACCGA
CGAGTCCAGTGGGGAATTACTTAAAGTTAGCCCACGCAATTAG

Aldose sugar DH from *T. thermophilus*
PDB: 2ISM

Molecular weight [Da]: 39648.09
ε_{280} [$M^{-1}cm^{-1}$]: 48360

Protein Sequence

MDRRRFLVGLLGLGLARGQGLRVEEVVGGLEVPWALAFLPDGGMLIAERPGRIRLFREGRLSTYAEL
SVYHRGESGLLGLALHPRFPQEPYVYAYRTVAEGGLRNQVVRLRHLGERGVLDRVVLDGIPARPHGL
HSGGRIAFGPDGMLYVTTGEVYERELAQDLASLGGKILRLTPEGEPAPGNPFLGRRGARPEVYSLGH
RNPQGLAWHPKTGELFSSEHGPSGEQGYGHDEVNLIVPGGNYGWPRVVGRGNDPRYRDPLYFWP
QGFPPGNLAFFRGDLYVAGLRGQALLRLVLEGERGRWRVLRVETALSGFGRLREVQVGPDGALYVT
TSNRDGRGQVRPGDDRVLRLLHHHHHH

DNA Sequence

ATGGATCGTCGTCGTTTCCTGGTTGGTCTGCTGGGTCTGGGGCTGGCACGTGGTCAGGGTCTGC
GTGTTGAAGAAGTTGTTGGTGGTCTGGAAGTTCCGTGGGCACTCGCTTTTCTGCCGGATGGTGG
TATGCTGATTGCAGAACGTCCGGGTCGTATTCGTCTGTTCCGTGAAGGTCGTCTTAGCACCTATGC
AGAACTTAGCGTTTATCATCGTGGTGAATCGGGTCTTCTCGGTCTGGCACTGCATCCCCGTTTCC
CGCAAGAACCGTATGTTTATGCATATCGTACCGTTGCAGAGGGTGGTCTGCGTAACCAGGTTGTT
CGTCTGCGTCATCTGGGTGAGCGTGGTGTTCTCGATCGTGTTGTTCTGGATGGTATTCCGGCACG
TCCGCATGGTCTGCATTCGGGTGGTCGTATTGCATTTGGTCCGGATGGTATGCTGTATGTTACCAC
TGGTGAAGTTTATGAACGTGAACTGGCACAGGATCTGGCAAGCCTCGGTGGTAAAATTCTGCGTC
TGACCCCGGAGGGTGAACCGGCACCGGGGAACCCGTTTCTGGGTCGCCGTGGTGCACGTCCG
GAAGTTTACTCTCTGGGTCATCGTAACCCGCAGGGTCTGGCATGGCATCCCAAAACCGGTGAACT
TTTTAGCTCTGAACATGGTCCGTCTGGTGAACAGGGTTACGGTCATGATGAAGTTAACCTGATTGT
TCCGGGTGGTAACTATGGTTGGCCGCGTGTTGTTGGTCGTGGTAACGATCCGCGTTATCGTGATC
CGCTGTATTTTTGGCCGCAGGGTTTTCCGCCGGGTAACCTGGCATTTTTTCGTGGTGATCTGTATG
TTGCAGGTCTGCGTGGTCAGGCACTGCTCCGTCTGGTTCTGGAAGGTGAACGTGGTCGTTGGC
GTGTACTCCGTGTTGAAACCGCACTTAGCGGTTTTGGTCGTCTTCGTGAAGTTCAGGTTGGTCCG
GACGGTGCACTTTATGTTACCACCAGCAACCGTGATGGTCGTGGTCAGGTTCGTCCGGGTGATG
ATCGTGTTCTGCGTCTGCTTCACCATCATCACCACCATTAA

Aldose sugar DH from *A. calcoaceticus*
PDB: 1C9U

Note: The protein sequence contains a **leader peptide**, which is cleaved and thus not present in the purified enzyme.

Molecular weight [Da]: 53550.46 (with leader peptide)
ε_{280} [$M^{-1}cm^{-1}$]: 71865

Molecular weight [Da]: 51073.41 (without leader peptide)
ε_{280} [$M^{-1}cm^{-1}$]: 71865
Protein Sequence

MNKHLLAKIALLGAAQLVTLSAFADVPLTPSQFAKAKTENFDKKVILSNLNKPHALLWGPDNQIWLTE
RATGKILRVNPESGSVKTVFQVPEIVNDADGQNGLLGFAFHPDFKNIPYIYISGTFKNPKSTDKELPNQ
TIIRRYTYNKSTDTLEKPVDLLAGLPSSKDHQSGRLVIGPDQKIYYTIGDQGRNQLAYLFLPNQAQHTP
TQQELNGKDYHTYMGKVLRLNLDGSIPKDNPSFNGVVSHIYTLGHRNPQGLAFTPNGKLLQSEQGPN
SDDEINLIVKGGNYGWPNVAGYKDDSGYAYANYSAAANKSIKDLAQNGVKVAAGVPVTKESEWTGKN
FVPPLKTLYTVQDTYNYNDPTCGEMTYICWPTVAPSSAYVYKGGKKAITGWENTLLVPSLKRGVIFRIK
LDPTYSTTYDDAVPMFKSNNRYRDVIASPDGNVLYVLTDTAGNVQKDDGSVTNTLENPGSLIKFTYKA
KHHHHHH

DNA Sequence

ATGAATAAACATTTACTGGCAAAAATTGCTTTATTAGGCGCCGCTCAGCTGGTTACACTCTCAGCAT
TTGCTGATGTTCCGTTAACGCCGAGTCAGTTCGCAAAAGCAAAAACAGAAAACTTTGACAAAAAA
GTTATTTTAAGTAATCTGAATAAGCCTCATGCATTACTGTGGGGGCCGGATAATCAGATTTGGCTGA
CAGAGCGCGCAACAGGTAAGATTCTGCGAGTGAATCCGGAGTCGGGTTCTGTGAAAACAGTTTTT
CAAGTGCCGGAGATTGTGAATGATGCAGATGGGCAGAATGGTTTACTGGGCTTTGCATTCCATCC
GGATTTTAAAAATATTCCGTATATCTACATTTCTGGTACATTTAAAAATCCTAAAAGTACAGATAAAGA
ATTACCGAATCAGACGATTATTCGTCGTTATACATATAATAAATCTACAGATACTCTCGAGAAGCCGG
TGGATCTGTTAGCAGGGCTGCCTTCTTCGAAAGACCATCAAAGTGGTCGTCTCGTCATTGGTCCG
GATCAGAAGATTTATTATACTATTGGTGACCAGGGGCGTAACCAATTAGCATATCTGTTCTTACCGA
ATCAGGCACAGCATACTCCGACGCAGCAGGAATTAAATGGCAAAGACTATCACACATATATGGGTA
AAGTGCTCCGCCTGAATTTAGATGGGTCTATTCCGAAGGATAATCCGTCTTTTAACGGGGTGGTTT
CTCATATTTATACATTAGGGCATCGTAATCCTCAAGGCCTGGCATTCACGCCGAATGGTAAATTATTA
CAAAGTGAACAGGGCCCGAACAGTGACGATGAAATTAACCTCATTGTCAAAGGTGGCAATTATGG
TTGGCCGAATGTGGCAGGTTATAAAGATGATTCTGGCTATGCATATGCAAATTATTCTGCAGCAGCT
AATAAGTCTATTAAGGATCTGGCACAGAATGGGGTGAAAGTGGCTGCAGGGGTTCCGGTGACTAA
AGAAAGTGAATGGACGGGTAAAAACTTTGTCCCGCCGCTGAAAACGCTGTATACAGTTCAGGATA
CATACAACTATAACGATCCGACGTGCGGGGAGATGACATATATTTGCTGGCCGACAGTTGCTCCTT
CTAGTGCTTATGTCTATAAGGGCGGTAAAAAAGCAATTACGGGTTGGGAAAATACACTGTTAGTTC
CGAGTCTGAAACGCGGTGTTATTTTCCGTATTAAGCTGGATCCGACGTATTCTACGACGTATGATG
ACGCAGTGCCTATGTTTAAGTCTAACAACCGTTATCGTGATGTGATTGCATCTCCGGATGGGAATG
TCCTGTATGTGCTGACGGACACGGCTGGGAATGTTCAGAAAGATGATGGCTCTGTGACAAATACA
CTGGAAAATCCGGGGAGTCTCATTAAGTTTACATATAAGGCAAAGCACCATCATCACCACCATTAA

Alcohol DH from *P. putida* (PedH F412V/W561A)

PDB: 6ZCV

Molecular weight [Da]: 63097.37
ε_{280} [$M^{-1}cm^{-1}$]: 141415

Protein Sequence

MAVSNEEILQDPKNPQQIVTNGLGVQGQRYSPLDLLNVNNVKELRPVWAFSFGGEKQRGQQAQPLI
KDGVMYLTGSYSRVFAVDARTGKKLWQYDARLPDDIRPCCDVINRGVALYGNLVFFGTLDAKLVALNK
DTGKVVWSKKVADHKEGYSISAAPMIVNGKLITGVAGGEFGVVGKIQAYNPENGELLWMRPTVEGHM
GYVYKDGKAIENGISGGEAGKTWPGDLWKTGGAAPWLGGYYDPETNLILFGTGNPAPWNSHLRPGD
NLYSSSRLALNPDDGTIKWHFQSTPHDGWDFDGVNELISFNYKDGGKEVKAAATADRNGFFYVLDRT
NGKFIRGFPFVDKITWATGLDKDGRPIYNDASRPGAPGSEAKGSSVFVAPAVLGAKNWMPMAYNKDT
GLFYVPSNEWGMDIWNEGIAYKKGAAFLGAGFTIKPLNEDYIGVLRAIDPVSGKEVWRHKNYAPLWG
GVLTTKGNLVFTGTPEGFLQAFNAKTGDKVWEFQTGSGVLGSPVTWEMDGEQYVSVVSGWGGAVP
LAGGEVAKRVKDFNQGGMLWTFKLPKQLQQTASVKPLEHHHHHH

DNA Sequence

ATGGCAGTGTCTAACGAGGAGATTCTGCAAGATCCAAAGAATCCACAACAAATTGTAACGAACGG
ATTAGGAGTACAAGGTCAACGTTATTCGCCTTTGGATTTGCTGAACGTTAACAATGTGAAAGAATTG
CGTCCTGTATGGGCTTTCTCGTTTGGTGGCGAGAAACAACGTGGTCAACAAGCGCAACCATTAAT
TAAAGATGGCGTTATGTATCTCACGGGAAGCTATAGCCGCGTATTTGCGGTTGACGCACGTACTGG

TAAGAAGTTGTGGCAGTATGACGCCCGTCTCCCCGACGATATTCGTCCATGTTGTGATGTTATTAAT
CGTGGGGTGGCCCTTTATGGTAATTTAGTATTCTTTGGAACTCTCGATGCGAAACTTGTAGCGCTT
AATAAAGATACGGGTAAAGTAGTTTGGTCAAAGAAAGTAGCGGATCATAAAGAGGGATATTCGATTA
GTGCTGCCCCCATGATTGTGAACGGAAAATTGATTACCGGTGTAGCAGGTGGTGAATTTGGGGTT
GTCGGGAAAATTCAAGCATATAATCCCGAAAATGGTGAGTTATTGTGGATGCGTCCTACAGTCGAG
GGCCACATGGGTTACGTATATAAAGACGGTAAAGCTATTGAAAATGGCATCAGTGGCGGCGAAGC
CGGGAAAACGTGGCCAGGGGATCTCTGGAAAACTGGTGGTGCGGCCCCCTGGTTAGGCGGCTA
TTATGATCCGGAGACGAATTTGATTTTATTCGGGACGGGGAATCCTGCACCTTGGAATAGCCATTT
GCGTCCAGGCGATAATCTTTATAGCTCGTCACGTCTTGCCCTTAATCCAGATGATGGTACGATTAAA
TGGCATTTTCAATCGACACCCCACGATGGTTGGGATTTTGATGGTGTGAATGAACTCATTAGTTTTA
ATTATAAAGATGGTGGAAAAGAAGTGAAAGCAGCAGCTACCGCGGATCGTAATGGCTTCTTCTATG
TATTAGATCGTACGAATGGTAAATTTATTCGTGGTTTTCCTTTTGTAGATAAAATTACTTGGGCGACC
GGTCTGGATAAAGATGGGCGCCCCATTTATAATGATGCAAGTCGTCCTGGGGCCCCGGGGTCGG
AAGCGAAAGGGTCATCCGTATTTGTGGCCCCAGCGGTACTGGGTGCGAAGAATTGGATGCCAAT
GGCGTATAATAAAGATACGGGCTTGTTTTATGTACCAAGTAATGAATGGGGAATGGATATTTGGAAT
GAGGGAATTGCATACAAGAAGGGCGCCGCCTTTCTGGGCGCGGGGTTTACTATTAAACCACTTAA
CGAGGATTATATTGGTGTTCTTCGTGCAATTGATCCCGTTTCCGGGAAAGAGGTCTGGCGTCATAA
GAATTACGCCCCATTATGGGGTGGCGTTCTTACTACGAAAGGGAATTTAGTGTTTACTGGAACTCC
TGAAGGGTTTCTTCAAGCGTTTAATGCCAAAACTGGGGATAAAGTTTGGGAGTTTCAAACTGGTAG
CGGTGTATTGGGGTCACCGGTGACGTGGGAGATGGATGGTGAACAGTATGTCTCCGTGGTGTCG
GGTTGGGGTGGTGCAGTCCCTTTAGCGGGTGGAGAGGTAGCGAAGCGTGTGAAAGATTTTAATC
AAGGTGGAATGTTGTGGACATTTAAACTGCCGAAACAACTGCAACAGACCGCCTCGGTTAAACCG
CTCGAGCACCACCACCACCACCACTGA

S2.3 Protein expression, purification and reconstitution

Aldose sugar DH from *E. coli* (YliI)

Expression: Chemically competent *E. coli* BL21(DE3) were transformed with plasmid encoding the aldose sugar DH (YliI) bearing an N-terminal His$_6$-tag and grown on agar plates containing kanamycin. One colony was inoculated in 50 mL of LB medium containing kanamycin and the cells were cultured overnight at 37 °C, shaking at 180 rpm. A 5 L Erlenmeyer flask containing LB medium (1.5 L) and kanamycin (final concentration of 30 µg/mL) was inoculated with 15 mL of a pre-culture. The culture was incubated at 37 °C (180 rpm, orbit diameter 2.5 cm) until reaching an OD_{600} of 0.6–0.8 and gene expression was induced with IPTG (final concentration 0.5 mM) followed by incubation at 18 °C for additional 16 h. Cells were harvested by centrifugation (20 min, 4100 rcf, 4 °C) and stored at -20 °C as wet cell pellet.

Purification: Frozen cells were thawed on ice and resuspended in Ni-NTA wash buffer (25 mM HEPES pH 7.5, 100 mM NaCl, 30 mM imidazole) to a final concentration of 2 mL/g of wet cells. The resuspended cells were supplemented a spatula tip of lysozyme (Carl Roth GmbH + Co. KG, ≥45000 FIP U/mg) and DNaseI (AppliChem GmbH, ≥ 3000 U/mg). Followed by 30 min incubation on ice, the cells were lysed via sonication using a Branson SFX 500 sonifier (Emerson Electric Co., St. Louis, MO, USA; power-on time: 10 min, pulsed time: 5 s on, 7 s off, 35% power). After centrifugation (40 min, 11,000 rcf, 6 °C), the cleared cell lysate was used for affinity chromatography. The cleared lysate was loaded onto a column packed with 3 mL Ni-NTA agarose resin (Qiagen N.V., Venlo, Netherlands) equilibrated with 3–5 column volumes of Ni-NTA wash buffer. The flow-through of the first loading was collected and loaded onto the column a second time. After washing the column with 10 CV Ni-NTA wash buffer, YliI was eluted in 3 CV Ni-NTA elution buffer (25 mM HEPES pH 7.5, 100 mM NaCl, 300 mM imidazole), and all elution fractions were combined. The combined protein solutions were concentrated using Merck Millipore Amicon Ultra centrifugal

filter devices (Merck KGaA,Darmstadt, Germany; 30 kDa cut-off, 4000 rcf, 4 °C) to a final concentration of 7–8 mg/mL.

Enzyme reconstitution: To reconstitute YliI with PQQ, purified protein was incubated with a 10-fold molar excess of PQQ in 25 mM HEPES, 100 mM NaCl, 1 mM $CaCl_2$, pH 7.5, at 4 °C for 16 h. Unbound PQQ was removed by passing the mixture over a Sephadex G-25 column (PD-10; *Cytiva*, Marlborough, MA, USA). The protein samples were frozen in liquid nitrogen and stored at -70 °C.

Aldose sugar DH from *T. thermophilus*

Expression: Chemically competent *E. coli* BL21(DE3) were transformed with plasmid encoding the aldose sugar DH bearing a C-terminal His_6-tag and grown on agar plates containing kanamycin. One colony was inoculated in 50 mL of LB medium containing kanamycin and the cells were cultured overnight at 37 °C, shaking at 180 rpm. A 5 L Erlenmeyer flask containing LB medium (1.5 L) and kanamycin (final concentration of 30 µg/mL) was inoculated with 15 mL of a pre-culture. The culture was incubated at 37 °C (180 rpm, orbit diameter 2.5 cm) until reaching an OD_{600} of 0.6–0.8 and gene expression was induced with IPTG (final concentration 0.5 mM) followed by incubation at 25 °C for additional 16 h. Cells were harvested by centrifugation (20 min, 4100 rcf, 6 °C) and stored at -20 °C as wet cell pellet.

Purification: Frozen cells were thawed on ice and resuspended in resuspension buffer (50 mM Tris-HCl, pH 7.5, 500 mM NaCl) to a final concentration of 2 mL/g of wet cells. The resuspended cells were supplemented a spatula tip of lysozyme (Carl Roth GmbH + Co. KG, ≥45000 FIP U/mg) and DNaseI (AppliChem GmbH, ≥ 3000 U/mg). Followed by 30 min incubation on ice, the cells were lysed via sonication using a Branson SFX 500 sonifier (Emerson Electric Co., St. Louis, MO, USA; power-on time: 10 min, pulsed time: 5 s on, 7 s off, 35% power) and heated at 70 °C for 10 mins. After that, heat-denaturized proteins and cell debris were removed by centrifugation (40 min, 11,000 rcf, 6 °C), the cleared cell lysate was used for affinity chromatography. The cleared lysate was loaded onto a column packed with 3 mL Ni-NTA agarose resin (Qiagen N.V., Venlo, Netherlands) equilibrated with 3–5 column volumes of Ni-NTA wash buffer buffer (50 mM Tris-HCl, pH 7.5, 500 mM NaCl, 30 mM imidazole). The flow-through of the first loading was collected and loaded onto the column a second time. After washing the column with 10 CV Ni-NTA wash buffer, aldose sugar DH was eluted in 3 CV Ni-NTA elution buffer (50 mM Tris-HCl, pH 7.5, 500 mM NaCl, 300 mM imidazole), and all elution fractions were combined. The combined protein solutions were concentrated using Merck Millipore Amicon Ultra centrifugal filter devices (Merck KGaA,Darmstadt, Germany; 30 kDa cut-off, 4000 rcf, 4 °C) to a final concentration of 7–8 mg/mL. The buffer is exchanged by dialyzing the protein in 1L buffer (50 mM Tris-HCl, pH 7.5, 500 mM NaCl) o/n and again with 1L fresh buffer on next day for 1 h.

Enzyme reconstitution: The purified protein was incubated with a 10-fold molar excess of PQQ in 20 mM Tris-HCl, 100 mM NaCl, 1 mM $CaCl_2$, pH 7.5, at 25 °C for 16 h. Unbound PQQ was removed by passing the mixture over a Sephadex G-25 column (PD-10; *Cytiva*, Marlborough, MA, USA). The protein samples were frozen in liquid nitrogen and stored at -70 °C.

Aldose sugar DH from *A. calcoaceticus*

Expression: Chemically competent *E. coli* BL21(DE3) were transformed with plasmid encoding the aldose sugar DH bearing a C-terminal His_6-tag and grown on agar plates containing kanamycin. One colony was inoculated in 50 mL of LB medium containing kanamycin and the cells were cultured overnight at 37 °C, shaking at 180 rpm. A 5 L Erlenmeyer flask containing LB medium (1.5 L) and kanamycin (final concentration of 30 µg/mL) was inoculated with 15 mL of a pre-culture. The culture was incubated at 37 °C (180 rpm, orbit diameter 2.5 cm) until reaching an OD_{600} of 0.6–0.8 and gene expression was induced with IPTG (final concentration 0.5 mM) followed by incubation at 18 °C for additional 16 h. Cells were harvested by centrifugation (20 min, 4100 rcf, 6 °C) and stored at -20 °C as wet cell pellet.

Purification: Frozen cells were thawed on ice and resuspended in resuspension buffer (20 mM Tris-HCl, pH 7.5, 100 mM NaCl) to a final concentration of 2 mL/g of wet cells. The resuspended cells were supplemented a spatula tip of lysozyme (Carl Roth GmbH + Co. KG, ≥45000 FIP U/mg) and DNaseI (AppliChem GmbH, ≥ 3000 U/mg). Followed by 30 min incubation on ice, the cells were lysed via sonication using a Branson SFX 500 sonifier (Emerson Electric Co., St. Louis, MO, USA; power-on time: 10 min, pulsed time: 5 s on, 7 s off, 35% power). After centrifugation (40 min, 11,000 rcf, 6 °C), the cleared cell lysate was used for affinity chromatography. The cleared lysate was loaded onto a column packed with 3 mL Ni-NTA agarose resin (Qiagen N.V., Venlo, Netherlands) equilibrated with 3–5 column volumes of Ni-NTA wash buffer. The flow-through of the first loading was collected and loaded onto the column a second time. After washing the column with 10 CV Ni-NTA wash buffer (20 mM Tris-HCl, pH 7.5, 100 mM NaCl, 30 mM imidazole), aldose sugar DH was eluted in 3 CV Ni-NTA elution buffer (20 mM Tris-HCl, pH 7.5, 100 mM NaCl, 300 mM imidazole), and all elution fractions were combined. The combined protein solutions were concentrated using Merck Millipore Amicon Ultra centrifugal filter devices (Merck KGaA,Darmstadt, Germany; 30 kDa cut-off, 4000 rcf, 4 °C) to a final concentration of 7–8 mg/mL. The buffer was exchanged by dialyzing the protein in 1L buffer (20 mM Tris-HCl, pH 7.5, 100 mM NaCl) o/n and again with 1L fresh buffer on next day for 1 h.

Reconstitution: The purified protein was incubated with a 10-fold molar excess of PQQ in 20 mM Tris-HCl, 100 mM NaCl, 1 mM $CaCl_2$, pH 7.5, at 4 °C for 12 h. Unbound PQQ was removed by passing the mixture over a Sephadex G-25 column (PD-10; *Cytiva*, Marlborough, MA, USA). The protein samples were frozen in liquid nitrogen and stored at -70 °C.

Alcohol DH from *P. putida* (PedH F412V/W561A)

Expression and purification: Chemically competent *E. coli* BL21(DE3) were transformed with plasmid encoding the alcohol DH bearing a C-terminal His_6-tag and grown on agar plates containing kanamycin. The induction, expression, and purification protocols are identical to those of aldose sugar DH from *E. coli* (YliI).

Enzyme reconstitution: The overnight reconstitution of PedH with PQQ and La(III) led to the precipitation of the protein. For this purpose, PedH was reconstituted in situ by addition of 5-fold molar excess of PQQ and 3-fold molar excess of $LaCl_3$ in the buffer used.

S3 Native activity assays of PQQ enzymes

Aldose sugar DH from *E. coli* (YliI) and aldose sugar DH from *A. calcoaceticus*

To determine the native enzymatic activity of the two dehydrogenases, a coupled colorimetric assay using 2,6-dichloro-phenolindophenol (DCPIP) was performed. The assay buffer consisted of 100 µM DCPIP and 1 mM phenazine ethosulfate (PES) in ddH2O. The assay setup and protocol were adapted from Jahn *et al*.[1]
For the spectroscopic measurement, 300 µL assay buffer and 90 µL of a 2 M glucose solution were added to a Quartz cuvette (0.7 mL, d = 1 cm, Portman Instruments AG), followed by the addition of 10 µL of a 20 µM stock of dehydrogenase. The contents were quickly mixed and the absorption at 600 nm was measured over 450 seconds to verify the enzymatic activity.

Aldose sugar DH from *T. thermophilus*

Here, assay was performed as mentioned above and at 40 °C.

Alcohol DH from *P. putida* (PedH F412V/W561A)

The assay was performed essentially as above using ethanol as the substrate at 5% (v/v) final concentration. PedH was in situ reconstituted with a 5-fold molar excess of PQQ and a 3-fold molar excess of $LaCl_3$ prior to starting the reaction.

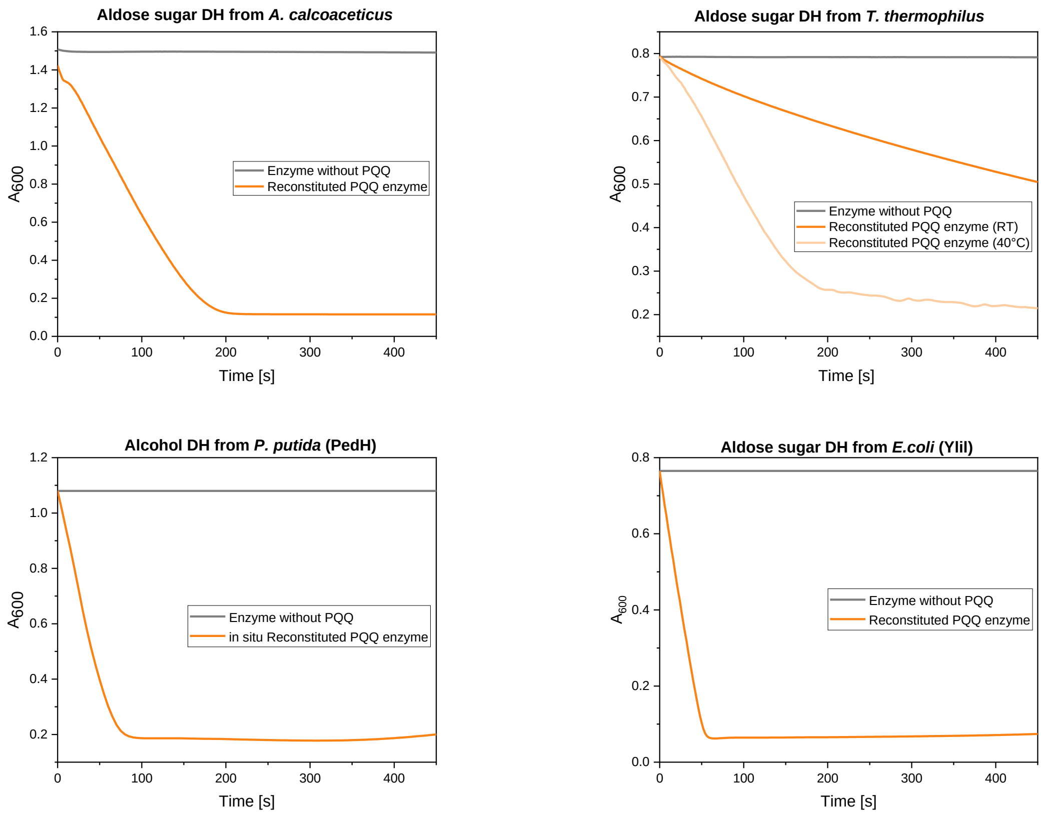

Figure S2: *DCPIP assay for the determination of the native enzymatic activity of all PQQ enzymes.*

S4 Protein mass spectrometry

Enzyme	MW [Da] (without Met1)	Observed Mass
Aldose sugar DH from *E. coli* (YliI)	39763.78 (39632.59)	39633
Aldose sugar DH from *T. thermophilus*	39648.09 (39516.90)	38290.5 [a]
Aldose sugar DH from *A. calcoaceticus*	51073.41	51072
Alcohol DH from *P. putida* (PedH F412V/W561A)	63097.37 (62966.17)	62965, 62997

[a] For aldose sugar DH from *T. thermophilus*, we observed a mass associated with the removal of first 11 amino acids, which is most likely an unassigned leader peptide that is cleaved.

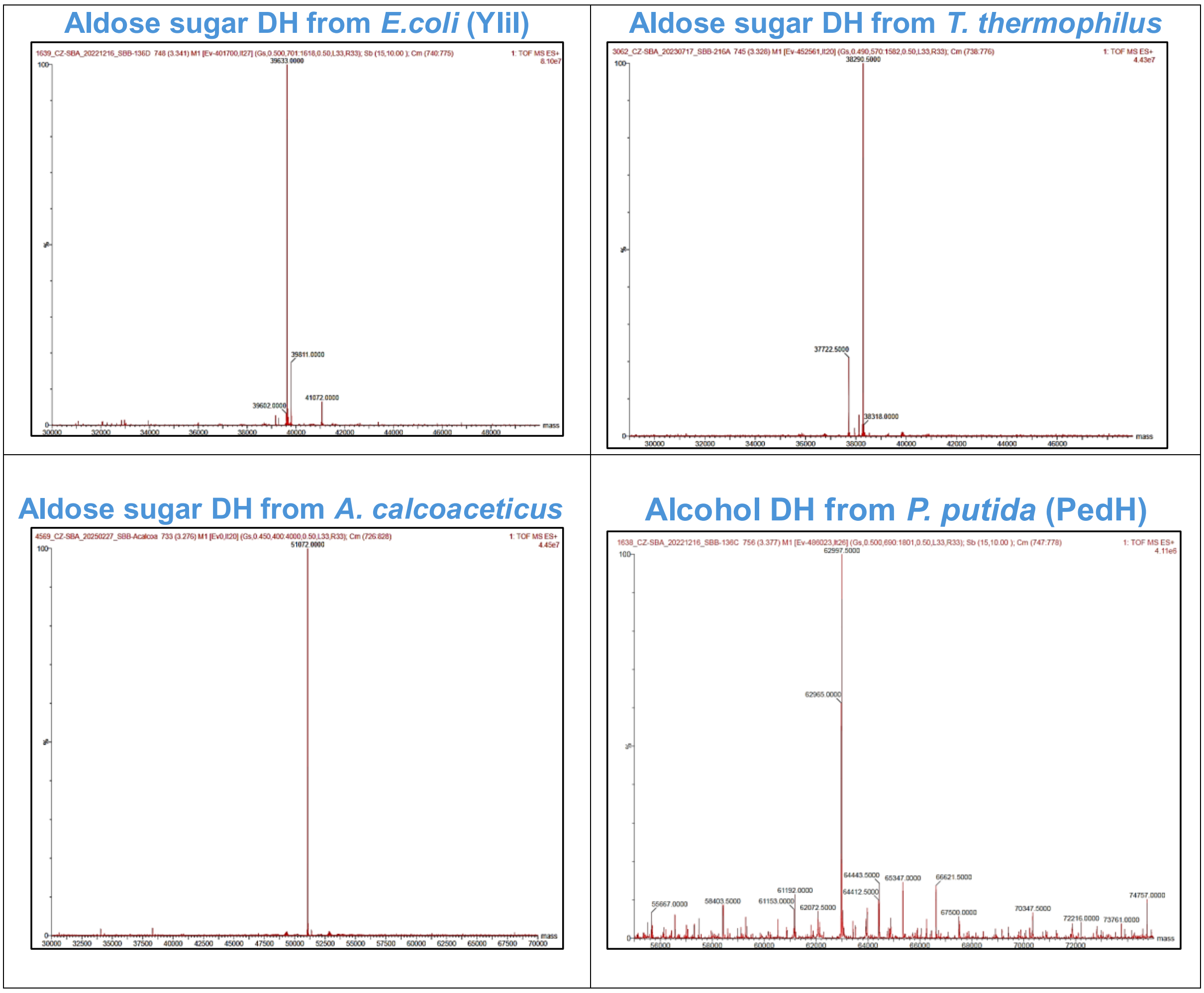

Figure S3: *Mass spectrometry analysis of PQQ enzymes.*

S5 Chemical syntheses

S5.1 General procedure (GP A) for the synthesis of $PQQMe_3$

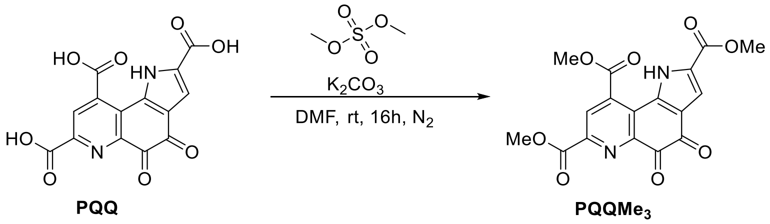

The procedure was adapted according to the literature procedure with slight modification.[2] In an oven-dried Schlenk flask equipped with magnetic stirrer bar, PQQ (500 mg, 1.51 mmol) was dissolved in dry DMF (20 mL) under N_2 atmosphere. K_2CO_3 (5.23 g, 37.85 mmol) was added to the solution under the flow of N_2. This is then followed by the addition of Me_2SO_4 (15.5 mL, 163.5 mmol) and the mixture was allowed to stir overnight at room temperature under N_2 atmosphere. The next day, 8 mL HCl (2M) was added to remove residual K_2CO_3 and quench the excess of Me_2SO_4. The mixture was then allowed to stir for an additional 5 hours. The precipitate was then filtered, washed with water (50 mL), and dried under high vacuum to afford pure $PQQMe_3$ as a bright orange powder (87%, 490 mg).

Trimethyl 4,5-dioxo-4,5-dihydro-1H-pyrrolo[2,3-f]quinoline-2,7,9-tricarboxylate, $PQQMe_3$

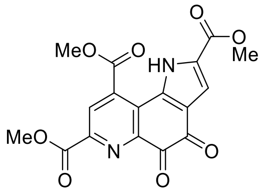

^{1}H NMR (400 MHz, DMSO-d_6): δ (ppm) = 12.52 (s, 1H), 8.57 (s, 1H), 7.30 (d, J = 2.2 Hz, 1H), 4.05 (s, 3H), 3.97 (s, 3H), 3.90 (s, 3H).

^{13}C NMR (126 MHz, DMSO-d_6): δ (ppm) = 177.0, 173.3, 166.6, 163.8, 159.9, 149.0, 145.7, 134.2, 133.5, 128.5, 126.6, 126.5, 125.0, 113.9, 54.2, 53.0, 52.4.

S5.2 General procedure (GP B) for the synthesis of the indole substrate (1)

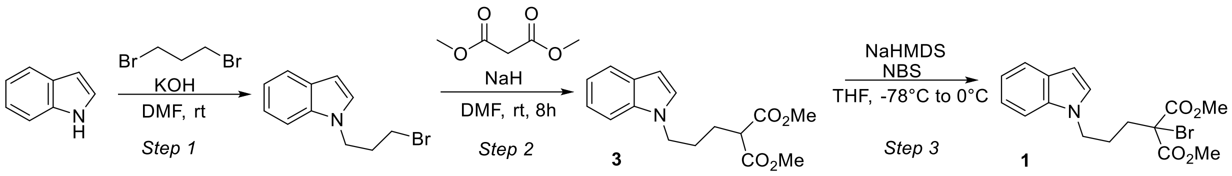

Step1

Step 1 was done according to the literature procedure with slight modification.[3] To a stirred solution of 12 g (60 mmol) of 1,3-dibromopropane in DMF (100 mL) were added 2.34 g (20 mmol) of indole and 1.14 g (20 mmol) of ground KOH powder. The reaction mixture was allowed to stir overnight. Subsequently, 100 mL of water was added, and the product was extracted with Et_2O (3 X 50 mL). The combined organic extracts were washed with water (100 mL) to remove DMF, followed by an additional wash with brine (100 mL). The organic layer was dried over Na_2SO_4 and then concentrated in vacuo. The residue was purified *via* silica gel column chromatography and eluted with petroleum ether: Et_2O – 10:1 to afford the corresponding product as an oil (71%, 3.4 g).

Step2

Step 2 was done according to the literature procedure.[4] A flame dried round bottom flask equipped with magnetic stirrer bar was charged with NaH (60% w/w dispersion in mineral oil, 1.21 g, 50.4 mmol). The flask was fitted with a rubber septum and N_2 balloon and followed by the addition of DMF (50 mL). Dimethyl malonate (63.0 mmol) was then added, and the mixture was allowed to stir for 30 min at room temperature. After 30 min have elapsed, a solution of 3.0 g (12.6 mmol) 1-(3-bromopropyl)-1*H*-indole in DMF (5 mL) was added dropwise and was allowed to stir overnight at room temperature. The reaction mixture was cooled to 0°C and the reaction was quenched by the dropwise addition of water (20 mL). The mixture was extracted with Et_2O (3 X 50 mL). The combined organic extracts were washed with water (100 mL) to remove DMF, followed by an additional wash with brine (100 mL). The organic layer was dried over Na_2SO_4 and then concentrated in vacuo. The residue was purified *via* silica gel column chromatography and eluted with petroleum ether: EtOAc – 4:1 to afford the corresponding product **3** as a yellow oil (69%, 2.50 g).

Dimethyl 2-(3-(1*H*-indol-1-yl)propyl)malonate, 3

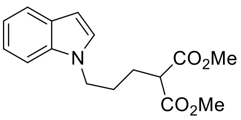

^{1}H NMR (400 MHz, $CDCl_3$): δ (ppm) **=** 7.63 (d, J = 8.0 Hz, 1H), 7.32 (d, J = 8.5 Hz, 1H), 7.21 (ddd, J = 8.5, 7.0, 1.0 Hz, 1H), 7.13 – 7.07 (m, 2H), 6.50 (d, J = 3.0 Hz, 1H), 4.14 (t, J = 6.5 Hz, 2H), 3.71 (s, 6H), 3.34 (t, J = 7.0 Hz, 1H), 1.99 – 1.84 (m, 4H).

^{13}C NMR (101 MHz, $CDCl_3$): δ (ppm) **=** 169.4, 135.8, 128.7, 127.6, 121.5, 121.0, 119.3, 109.2, 101.3, 52.5, 51.1, 45.8, 27.8, 26.2.

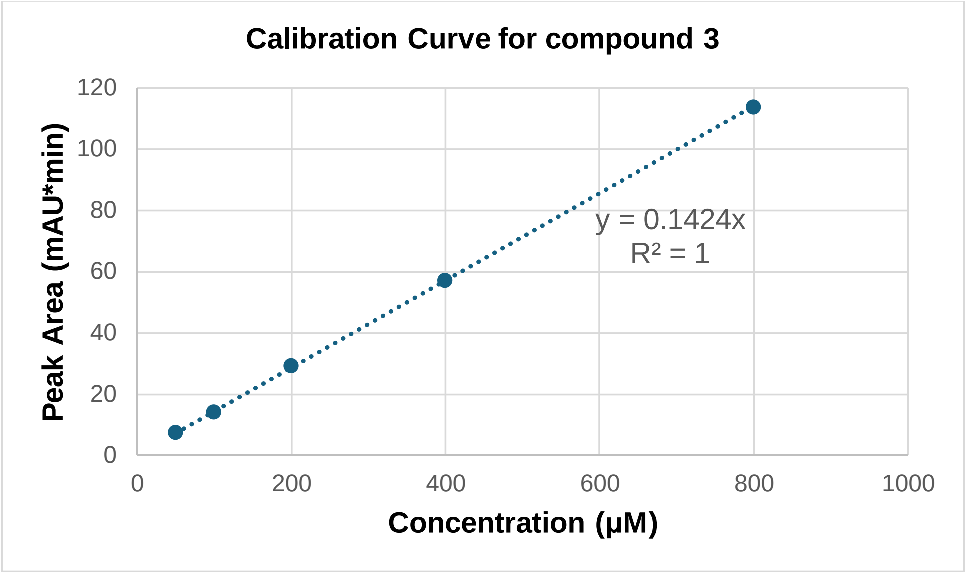

Step 3

Step 3 was done according to the literature procedure with slight modification.[5] A solution of 2 g (6.91 mmol) malonate 3 in anhydrous THF (50 mL) was added to an oven dried round bottom flask equipped with magnetic stirrer bar. The flask was fitted with a rubber septum and N_2 balloon and cooled to -78 °C. Subsequently, a 2.5 M solution of NaHMDS (3.0 mL, 7.60 mmol) in THF was added dropwise to the flask and the mixture was allowed to stir for 15 min. It was then followed by the addition of 1.35 g (7.60 mmol) *N*-Bromosuccinimide (NBS) and the mixture was allowed to gradually warm to room temperature over 4 h. The reaction was then quenched with the addition of water and the product was extracted with Et_2O (3 X 50 mL). The combined organic extracts were additionally washed with brine (100 mL). The organic layer was dried over Na_2SO_4 and then concentrated in vacuo. The residue was purified *via* silica gel column chromatography and eluted with petroleum ether: EtOAc – 93:7 to afford the corresponding product **1** as a yellow oil (75%, 1.90 g).

Dimethyl 2-(3-(1H-indol-1-yl)propyl)-2-bromomalonate, 1

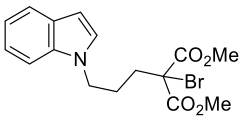

^{1}H NMR (500 MHz, $CDCl_3$): δ (ppm) **=** 7.63 (d, *J* = 7.8 Hz, 1H), 7.32 (dd, *J* = 8.1, 1.0 Hz, 1H), 7.23 – 7.19 (m, 1H), 7.13 – 7.08 (m, 2H), 6.50 (dd, *J* = 3.1, 0.9 Hz, 1H), 4.18 (t, *J* = 7.0 Hz, 2H), 3.72 (s, 6H), 2.33 – 2.25 (m, 2H), 2.07 – 1.96 (m, 2H).

^{13}C NMR (101 MHz, $CDCl_3$): δ (ppm) **=** 167.0, 135.8, 128.7, 127.5, 121.6, 121.0, 119.4, 109.2, 101.4, 61.8, 53.9, 45.6, 35.6, 26.2.

S5.3 General procedure (GP C) for the synthesis of the cyclized product standard (2) of indole substrate

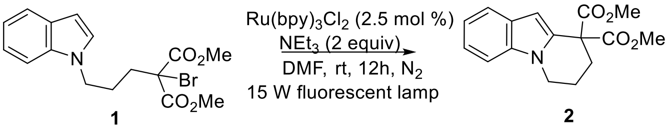

This procedure was adapted from the literature procedure.[5] An oven dried Schlenk flask (25 mL) equipped with a magnetic stirrer bar and septum was charged with tris(2,2'-bipyridyl)ruthenium(II) chloride hexahydrate (1.5 mg, 2.0 μmol), bromomalonate **1** (0.20 mmol), Et_3N (56 μL, 0.40 mmol) and 4 mL dry DMF. The mixture was then degassed by 3 cycles of freeze-pump-thaw method. The reaction mixture was irradiated with a 15 W fluorescent lamp for 12 hours. The reaction was quenched with water (50 mL) and extracted with Et_2O (25 mL) three times. The combined organic extracts were then washed with water (25 mL) to remove DMF, then washed once more with brine (25 mL). Combined organic extracts were dried over Na_2SO_4, then evaporated in *vacuo*. Crude mixture was then loaded onto silica column and purified with petroleum ether: EtOAc – 19:1 to afford pure product.

Dimethyl 7,8-dihydropyrido[1,2-a]indole-9,9(6H)-dicarboxylate, 2

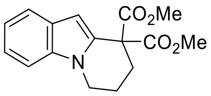

^{1}H NMR (400 MHz, $CDCl_3$): δ (ppm) **=** 7.59 (dd, *J* = 7.8, 1.0 Hz, 1H), 7.28 (d, *J* = 7.8 Hz, 1H), 7.20 (dt, *J* = 7.8, 0.9 Hz, 1H), 7.11 (dt, *J* = 7.8, 0.9 Hz, 1H), 6.59 (s, 1H), 4.08 (t, *J* = 6.2 Hz, 2H), 3.80 (s, 6H), 2.57 – 2.49 (m, 2H), 2.18 – 2.06 (m, 2H).

^{13}C NMR (126 MHz, $CDCl_3$): δ (ppm) **=** 170.4, 136.3, 130.8, 127.4, 121.7, 120.7, 120.1, 109.1, 102.1, 56.1, 53.2, 41.9, 28.7, 19.8.

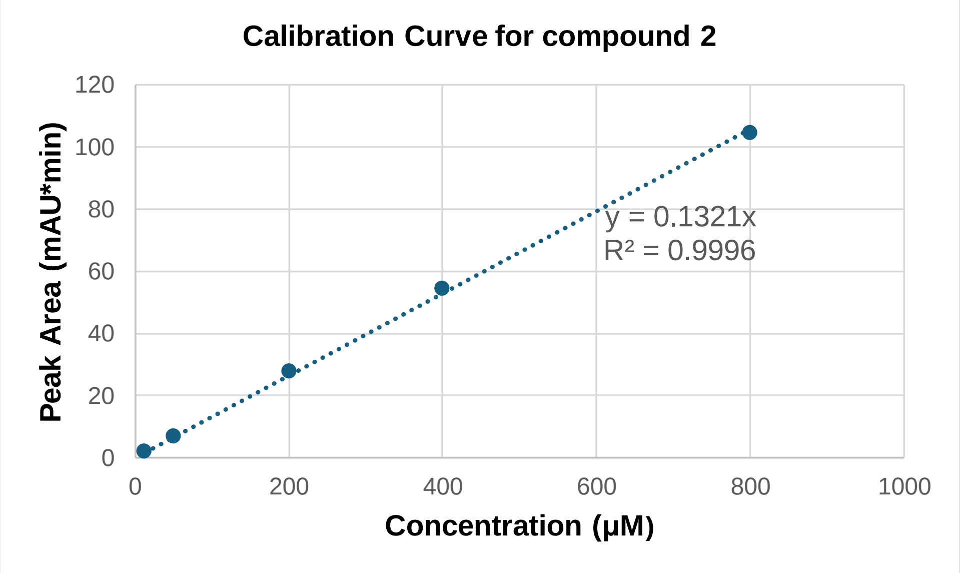

S5.4 General procedure (GP D) for the synthesis of α-chloroamides

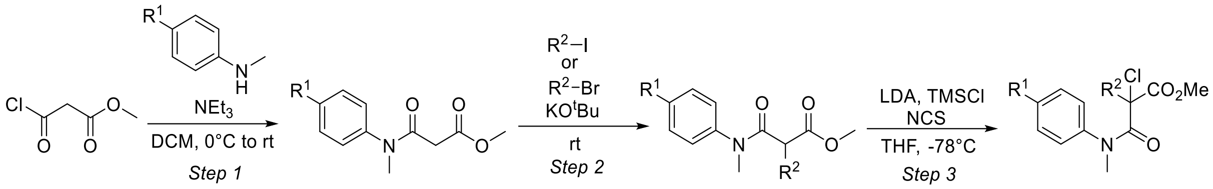

Step1

Step 1 was done according to the literature procedure.[6] A solution of N–alkylaniline (20.0 mmol, 1.0 equiv) and Et_3N (40 mmol, 2.0 equiv) in anhydrous CH_2Cl_2 (60 mL) was cooled to 0 °C. Methyl malonyl chloride (24 mmol, 1.2 equiv) was added dropwise. The mixture was then allowed to warm to room temperature and stirred for 2 h. The solvent was removed under reduced pressure. Crude was used without any further purification.

Step2

Step 2 was done according to the literature procedure.[7] A solution of β-amidoester (10 mmol, 1.0 equiv) in anhydrous DMF (50 mL) was prepared in an oven dried round bottom flask under N_2 atmosphere. To the solution, potassium *tert*-butoxide (12 mmol, 1.2 equiv) was added slowly and the resulting mixture was allowed to stir for 15 mins. It is then followed by the addition of alkyl iodide or alkyl bromide (10.5 mmol, 1.05 equiv). The reaction was allowed to stir overnight at room temperature. The reaction was quenched with water (50 mL) and followed by extraction with EtOAc (25 mL) three times. The combined organic extracts were then washed with water (50 mL) to remove DMF, then washed once more with brine (50 mL). Combined organic extracts were dried over Na_2SO_4, then evaporated in *vacuo*. Crude mixture was then loaded onto silica column and purified with petroleum ether: EtOAc – 4:1 to afford pure product.

Methyl 2-(methyl(phenyl)carbamoyl)butanoate, 6

Synthesized from methyl3-((4-methoxyphenyl)(methyl)amino)-3-oxopropanoate and ethyl iodide .

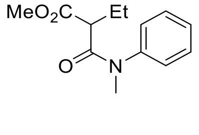

^{1}H NMR: (500 MHz, $CDCl_3$): δ (ppm) **=** 7.45-7.42 (t, J = 6.6 Hz, 2H), 7.38-7.35 (t, 1H), 7.24-7.20 (d, 2H), 3.65 (s, 3H), 3.30 (s, 3H), 3.27-3.24 (dd, 1H), 1.95-1.76 (m, 2H), 0.81 (t, J = 7.4 Hz, 3H).

^{13}C NMR (101 MHz, $CDCl_3$): δ (ppm) **=** 170.5, 169.0, 143.5, 129.8, 128.1, 127.6, 52.1, 50.5, 37.6, 22.8, 11.9.

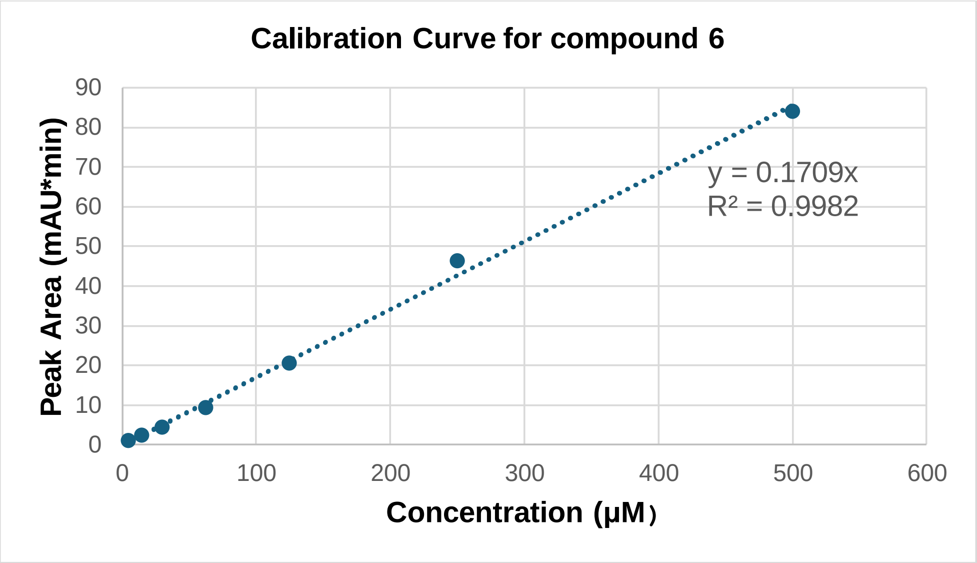

Methyl 2-((4-methoxyphenyl)(methyl)carbamoyl)butanoate, 17

Synthesized from methyl 3-((4-methoxyphenyl)(methyl)amino)-3-oxopropanoate and ethyl iodide.

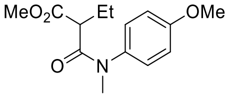

^{1}H NMR (400 MHz, $CDCl_3$): δ (ppm) **=** 7.17 – 7.11 (d, 2H), 6.95 – 6.89 (d, 2H), 3.83 (s, 3H), 3.66 (s, 3H), 3.29-3.26 (dd, 1H), 3.28 (s, 3H), 1.98 – 1.79 (m, 2H), 0.83 (t, J = 7.4 Hz, 3H).

^{13}C NMR (101 MHz, $CDCl_3$): δ (ppm) **=** 170.5, 169.4, 159.1, 136.3, 128.7, 114.9, 55.5, 52.1, 50.4, 37.7, 22.8, 11.9.

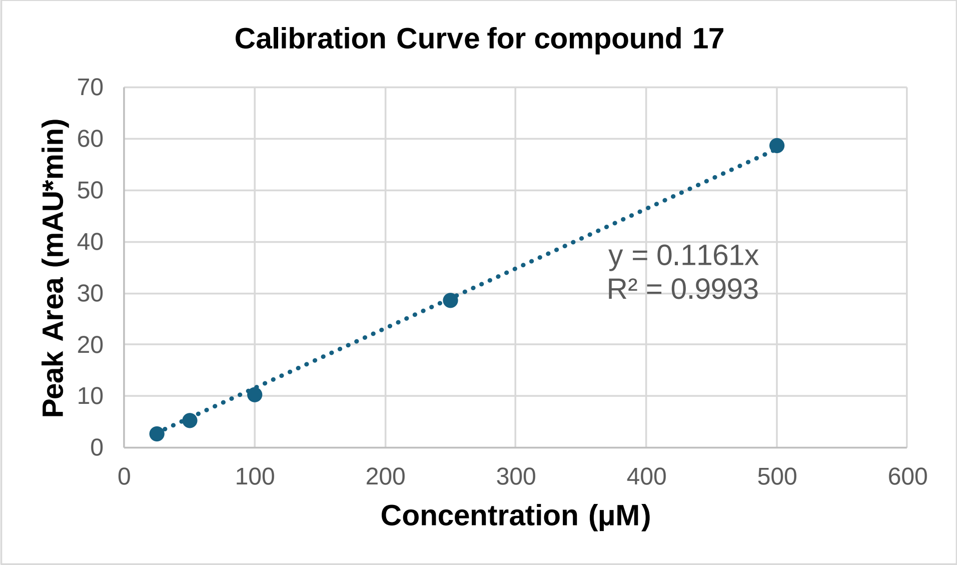

Methyl 2-(methyl(m-tolyl)carbamoyl)butanoate, 18

Synthesized from methyl 3-(methyl(m-tolyl)amino)-3-oxopropanoate and ethyl iodide.

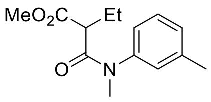

^{1}H NMR (400 MHz, $CDCl_3$): δ (ppm) **=** 7.29 (t, *J* = 7.8 Hz, 1H), 7.16 (d, *J* = 7.8 Hz, 1H), 7.03 (s, 1H), 7.03-7.01(d, 1H), 3.65 (s, 3H), 3.30 (s, 3H), 3.29-3.26 (dd, 1H), 2.38 (s, 3H), 1.97-1.81 (m, 2H), 0.82 (t, *J* = 7.4 Hz, 3H).

^{13}C NMR (101 MHz, $CDCl_3$): δ (ppm) **=** 170.6, 169.1, 143.4, 140.0, 129.6, 128.9, 128.2, 124.58, 52.2, 50.4, 37.6, 22.9, 21.3, 11.9.

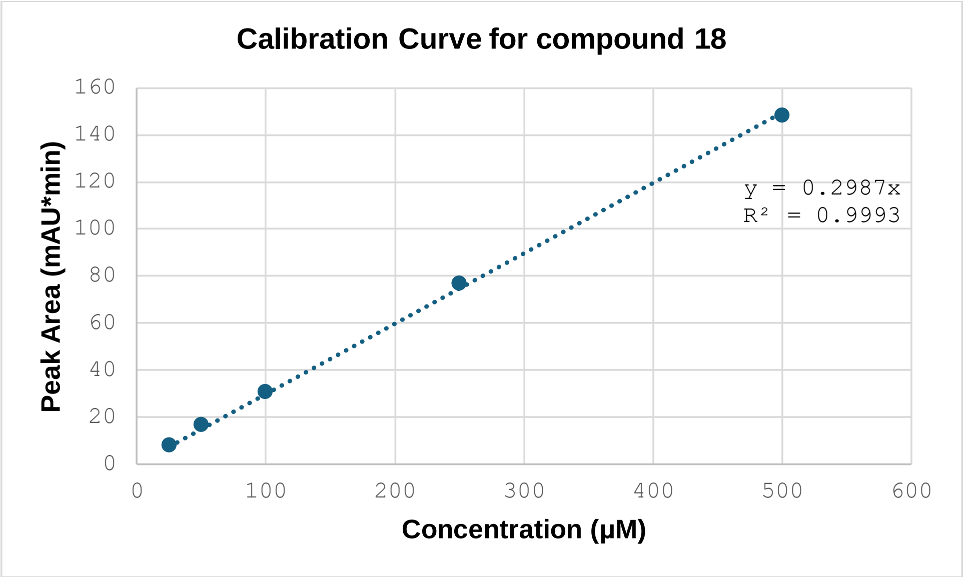

Methyl 2-methyl-3-(methyl(phenyl)amino)-3-oxopropanoate, 19

Synthesized from methyl 3-(methyl(phenyl)amino)-3-oxopropanoate and methyl iodide.

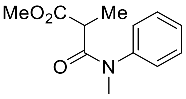

^{1}H NMR (500 MHz, $CDCl_3$): δ (ppm) **=** 7.45-7.42 (t, 2H), 7.39-7.32 (t, 1H), 7.25-.7.19 (d, 2H), 3.65 (s, 3H), 3.40 (q, *J* = 7.1 Hz, 1H), 3.29 (s, 3H), 1.29 (d, *J* = 7.0 Hz, 3H).

^{13}C NMR (101 MHz, $CDCl_3$): δ (ppm) **=** 171.1, 170.0, 143.6, 129.9, 128.2, 127.4, 52.2, 43.4, 37.6, 14.2.

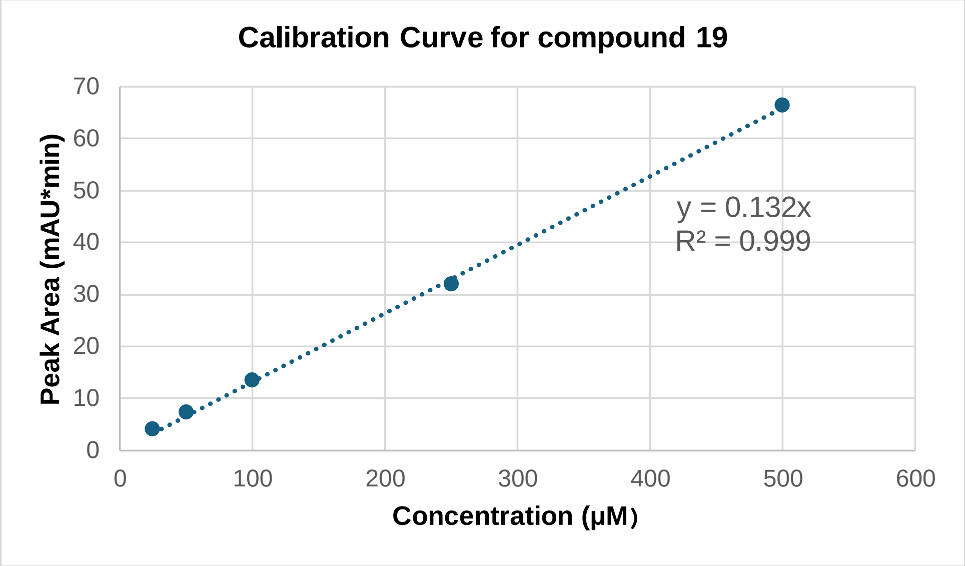

Methyl 2-benzyl-3-(methyl(phenyl)amino)-3-oxopropanoate, 20

Synthesized from methyl 3-(methyl(phenyl)amino)-3-oxopropanoate and benzyl bromide.

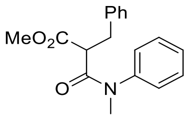

^{1}**H NMR** (400 MHz, $CDCl_3$): δ (ppm) **=** 7.33 – 7.20 (m, 6H), 7.09 – 6.96 (m, 2H), 6.65 (br, 2H), 3.71 (s, 3H), 3.57 (dd, J = 10.1, 5.1 Hz, 1H), 3.27 – 3.14 (m, 4H), 3.09 (dd, J = 13.4, 5.1 Hz, 1H).

^{13}**C NMR** (101 MHz, $CDCl_3$): δ (ppm) **=** 169.8, 168.4, 143.2, 138.4, 129.5, 129.2, 128.3, 127.99, 127.5, 126.5, 52.3, 50.9, 37.4, 35.3.

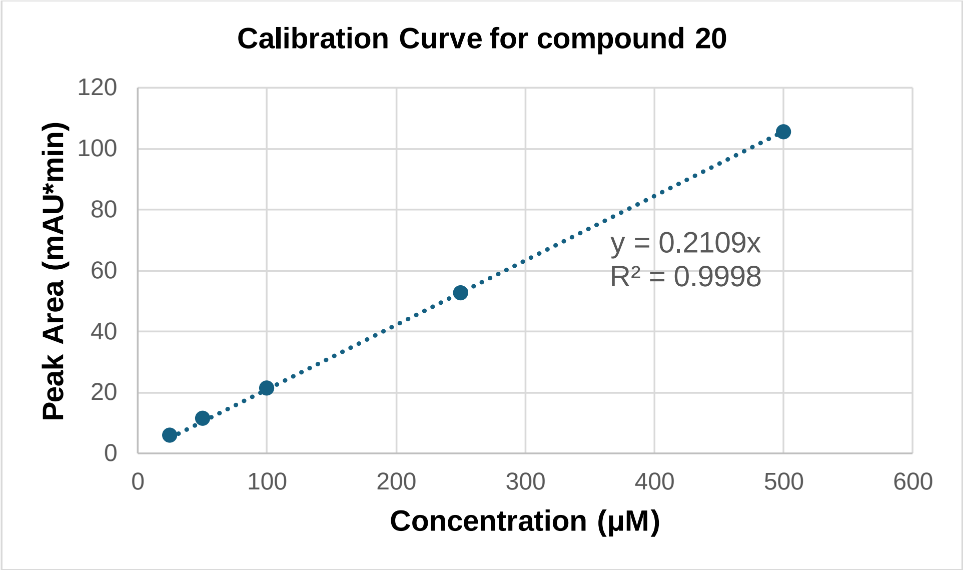

Step 3

Step 3 was done according to the literature procedure with slight modification.[7] An oven dried flask equipped with magnetic stirrer bar and septum was charged with 10 mL anhydrous THF and diisopropylamine (7.35 mmol, 1.05 equiv) under N_2 atmosphere, then cooled to 0 °C. To the above solution, n-BuLi (2.8 mL of a 2.5 M solution in hexanes, 7.0 mmol, 1.0 equiv) was gradually added and stirred at 0 °C for 30 min. The solution was then cooled to -78 °C and β-amidoester (7.0 mmol, 1.0 equiv) was added dropwise, and the reaction mixture was allowed to stir for 30 min. After which chlorotrimethylsilane (7.0 mmol, 1.0 equiv) was added and the solution was allowed to stir at room temperature for 4 hours. The reaction mixture was again cooled to -78 °C which is then followed by the addition of N-chlorosuccinimide (1.5 equiv) dissolved in anhydrous THF. The reaction mixture was allowed to stir at -78 °C for one hour, warmed back to room temperature and stirred for two hours. The reaction mixture was then quenched with water (20 mL), extracted three times with EtOAc (25 mL), and washed with brine and dried over Na_2SO_4. The solvent was evaporated in vacuo. Crude mixture was then loaded onto silica column and purified with petroleum ether: EtOAc – 9:1 to afford pure product.

Methyl 2-chloro-2-(methyl(phenyl)carbamoyl)butanoate, 4

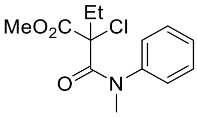

^{1}H NMR (400 MHz, C_6D_6): δ (ppm) **=** 7.07 (s, 2H), 6.92 (dt, J = 25.0, 7.6 Hz, 3H), 3.02 (s, 6H), 2.63 (dq, J = 14.9, 7.5 Hz, 1H), 2.52 (dq, J = 14.6, 7.3 Hz, 1H), 0.91 (t, J = 7.3 Hz, 3H).

^{13}C NMR (126 MHz, $CDCl_3$): δ (ppm) **=** 167.7, 165.8, 141.1, 129.9, 129.0, 71.4, 53.1, 40.6, 33.7, 8.2.

Methyl 2-chloro-2-((4-methoxyphenyl)(methyl)carbamoyl)butanoate, 7

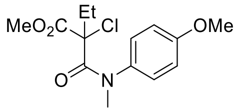

^{1}H NMR (500 MHz, C_6D_6): δ (ppm) **=** 7.15 (s, 2H), 6.56 (d, J = 8.5 Hz, 2H), 3.20 (s, 3H), 3.08 (s, 3H), 3.00 (s, 2H), 2.66 (p, J = 7.6 Hz, 1H), 2.53 (dq, J = 14.6, 7.3 Hz, 1H), 0.91 (t, J = 7.3 Hz, 3H).

^{13}C NMR (126 MHz, $CDCl_3$): δ (ppm) **=** 167.6, 166.2, 159.5, 133.5, 130.8, 128.02, 114.5, 71.4, 55.4, 53.1, 40.7, 33.7, 8.1.

Methyl 2-chloro-2-(methyl(m-tolyl)carbamoyl)butanoate, 8

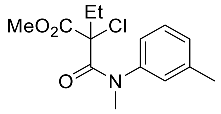

^{1}H NMR (500 MHz, C_6D_6): δ (ppm) **=** 7.04 – 6.86 (m, 3H), 6.80 – 6.73 (m, 1H), 3.06 (s,5H), 2.64 (q, J = 7.4 Hz, 1H), 2.53 (dq, J = 14.6, 7.3 Hz, 1H), 1.98 (s, 3H), 0.92 (t, J =7.4 Hz, 3H).

^{13}C NMR (126 MHz, $CDCl_3$): δ (ppm) **=** 167.6, 165.8, 140.9, 138.9, 130.3, 129.5, 128.8, 126.81, 71.5, 53.0, 40.6, 33.7, 21.2, 8.2.

Methyl 2-chloro-2-methyl-3-(methyl(phenyl)amino)-3-oxopropanoate, 9

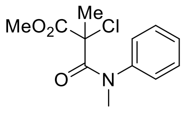

^{1}H NMR (500 MHz, C_6D_6): δ (ppm) **=** 7.04 (d, J = 7.3 Hz, 2H), 6.98 – 6.87 (m, 3H), 3.02 (d, J = 7.3 Hz, 6H), 2.03 (s, 3H).

^{13}C NMR (126 MHz, $CDCl_3$): δ (ppm) **=** 168.8, 165.8, 141.2, 129.8, 129.1, 66.0, 53.3, 40.6, 28.9.

Methyl 2-benzyl-2-chloro-3-(methyl(phenyl)amino)-3-oxopropanoate, 10

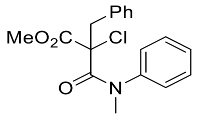

^{1}H NMR (400 MHz, $CDCl_3$): δ (ppm) **=** 7.33 (dt, *J* = 14.2, 7.1 Hz, 5H), 7.21 (d, *J* = 25.0 Hz, 5H), 3.62 (s, 2H), 3.37-3.11 (m, 6H).

^{13}C NMR (126 MHz, $CDCl_3$): δ (ppm) **=** 167.4, 165.9, 141.0, 134.1, 130.8, 129.0, 128.0, 127.9, 127.7, 127.5, 70.3, 52.9, 46.1, 40.8.

S5.5 General procedure (GP E) for the synthesis of methyl 3-(methyl(phenyl)amino)-3-oxo-2-phenylpropanoate

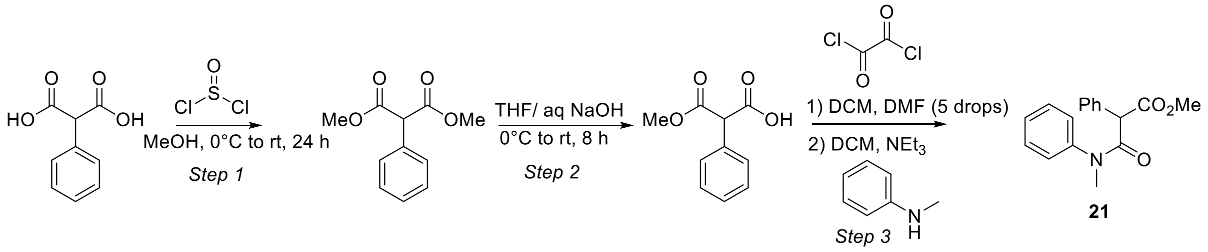

Step 1

Step 1 was done according to the literature procedure.[8] To a solution of phenylmalonic acid (5.40g, 30.0 mmol) in methanol (60 mL) was added thionyl chloride (5.45 mL, 75.0 mmol) at 0 °C. The mixture is then allowed to stir at room temperature for 24 hours. Once the reaction is complete as judged by TLC analysis, the solvent was removed under vacuum. The residue was then washed with hexane and dried under vacuum. The product was used for the next step without any further purification.

Step 2

Step 2 was done according to the literature procedure.[9] A round bottom flask equipped with magnetic stirrer bar was charged with Dimethyl malonate (12 mmol) dissolved in 20 mL of THF, and 200 mL of water. The reaction mixture was then placed in an ice-water bath and cooled to 0° C. Then, 40 mL of 0.5 M NaOH was gradually added with stirring until thin-layer chromatography confirmed the complete consumption of the starting diester. The reaction was allowed to stir for 60 minutes, and the reaction mixture was acidified with 1 M HCl at 0° C. It was then saturated with NaCl, extracted three times with EtOAc (25 mL each), dried over Na_2SO_4, and evaporated in vacuo. The product was used for the next step without any further purification.

Step 3

Step 3 was done according to the literature procedure.[10] The acid from the previous step (10 mmol) was dissolved in DCM (100 mL) and DMF (8 drops) were added. This is followed by the dropwise addition of oxalyl chloride (12 mmol, 1.2 equiv) at 0 °C. The reaction mixture was allowed to stir at 0 °C for 10 min at room temperature for 2 h. The resulting solution was then gradually added to a solution of N-substituted aniline (12 mmol, 1.2 equiv) and Et_3N (12 mmol, 1.2 equiv) in DCM (100 mL) at 0 °C. The mixture is stirred for 1 hour, warmed to room temperature and stirred for another 2 hours. The solvent was removed under vacuum which is then followed by the addition of water (50 mL). It is extracted three times with EtOAc (50 mL each), dried over Na_2SO_4, and evaporated in vacuo. The crude product is then purified by silica gel chromatography using petroleum ether: Et_2O – 10:1 to afford pure product.

Methyl 3-(methyl(phenyl)amino)-3-oxo-2-phenylpropanoate, 21

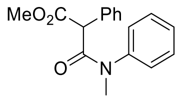

^{1}H NMR (400 MHz, $CDCl_3$): δ (ppm) **=** 7.41 – 7.29 (m, 3H), 7.24 – 7.17 (m, 3H), 7.11 – 6.98 (m, 4H), 4.57 (s, 1H), 3.65 (s, 3H), 3.22 (s, 3H).

^{13}C NMR (101 MHz, $CDCl_3$): δ (ppm) **=** 169.3, 167.8, 143.2, 133.5, 129.8, 129.4, 128.38, 128.32, 127.8, 55.7, 52.5, 37.8.

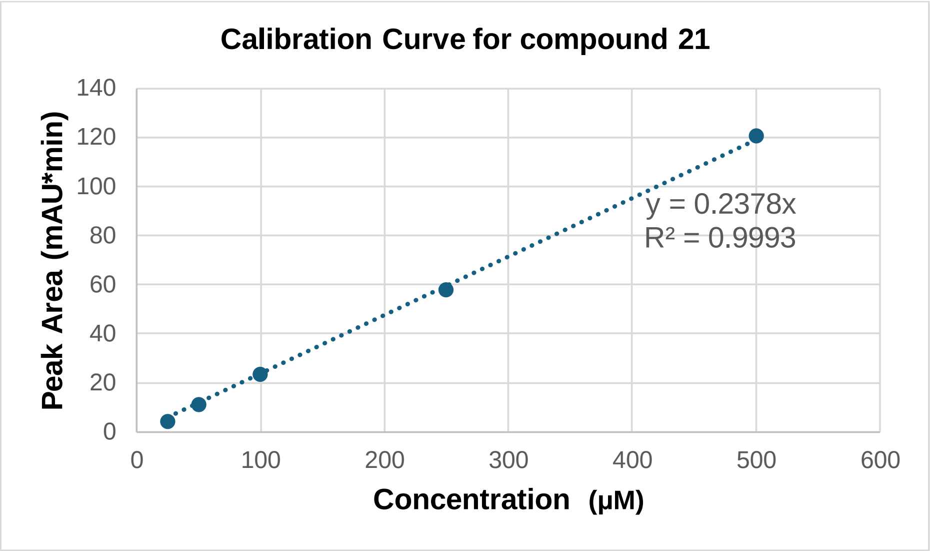

S5.6 Synthesis of methyl 2-chloro-3-(methyl(phenyl)amino)-3-oxo-2-phenylpropanoate, 11

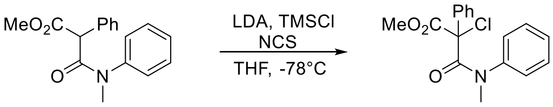

21 **11**

Compound **11** was synthesized by following Step *3* of General procedure (GP D) as mentioned above.

Methyl 2-chloro-3-(methyl(phenyl)amino)-3-oxo-2-phenylpropanoate, 11

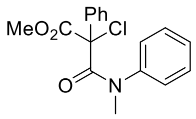

^{1}H NMR (500 MHz, C_6D_6): δ (ppm) **=** 7.73 (d, *J* = 7.5 Hz, 2H), 7.13 – 6.41 (m, 8H), 3.32 (s, 3H), 2.94 (br, 3H).

^{13}C NMR (126 MHz, $CDCl_3$): δ (ppm) **=** 167.7, 166.6, 143.9, 142.1, 135.9, 129.0, 128.3, 128.1, 128.0, 126.5, 74.0, 54.2, 41.5.

S5.7 General procedure (GP F) for the synthesis of racemic standards of cyclization products

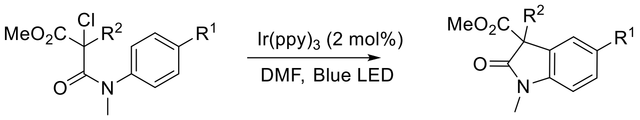

4, 7-11 **5, 12-16**

The synthesis was done according to the literature procedure with some modifications.[11] An oven dried 25 mL Schlenk flask equipped with magnetic stirrer bar and septum was charged with α-chloro-β-amidoester (0.4 mmol, 1.0 equiv) and Ir(ppy)$_3$ (5.2 mg, 0.008 mmol, 0.02 equiv). The flask was evacuated and backfilled with N_2 for three times and then anhydrous DMF (10 mL) added via syringe. The reaction mixture was degassed again. Finally, the puncture hole in the septum was sealed with parafilm. The reaction mixture was then irradiated with A160WE TUNA BLUE 40 W Kessil lamp for 24 hours with fan cooling. It was then followed by the addition of water (10 mL), three times extraction with EtOAc (25 mL each). The combined organic extracts were washed with water (10 mL) to remove residual DMF, then washed with brine (10 mL), dried over Na_2SO_4, and evaporated in vacuo. The crude product was then purified by silica gel chromatography using petroleum ether: Et_2O – 3:1 to afford pure product.

Methyl 3-ethyl-1-methyl-2-oxoindoline-3-carboxylate, 5

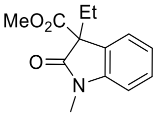

^{1}H NMR (500 MHz, $CDCl_3$): δ (ppm) **=** 7.31 (td, J = 7.8, 1.2 Hz, 1H), 7.27 (d, J = 1.3 Hz, 1H), 7.11 (td, J = 7.6, 1.0 Hz, 1H), 6.85 (d, J = 7.8 Hz, 1H), 3.64 (s, 3H), 3.23 (s, 3H),2.40 – 2.22 (m, 2H), 0.63 (t, J = 7.5 Hz, 3H).

^{13}C NMR (101 MHz, $CDCl_3$): δ (ppm) **=** 174.1, 170.0, 144.2, 129.0, 127.7, 123.4, 122.8, 108.2, 59.9, 52.8, 27.5, 26.4, 7.9.

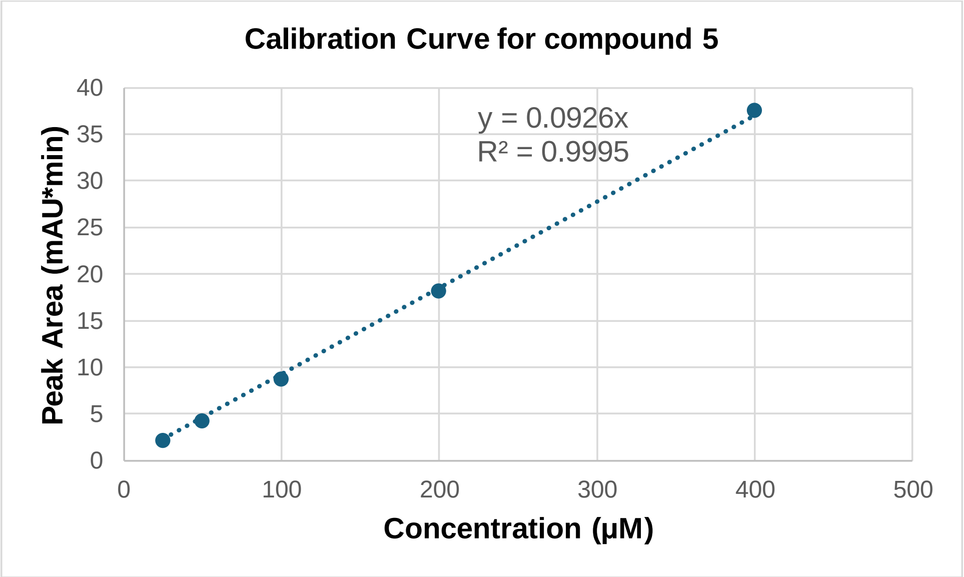

Methyl 3-ethyl-5-methoxy-1-methyl-2-oxoindoline-3-carboxylate, 12

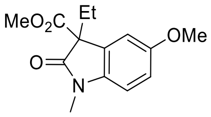

^{1}H NMR (500 MHz, $CDCl_3$): δ (ppm) **=** 6.86 – 6.81 (m, 2H), 6.75 (d, *J* = 8.3 Hz, 1H), 3.77 (s, 3H), 3.63 (s, 3H), 3.21 (s, 3H), 2.34 – 2.24 (dq, *J* = 14.7, 7.4, 6.8 Hz, 1H), 2.25 – 2.15 (m, 1H), 0.61 (t, *J* = 7.4 Hz, 3H).

^{13}C NMR (126 MHz, $CDCl_3$): δ (ppm) **=** 173.9, 170.1, 156.2, 137.7, 129.0, 113.3, 110.7, 108.7, 60.3, 55.8, 53.0, 27.5, 26.5, 8.0.

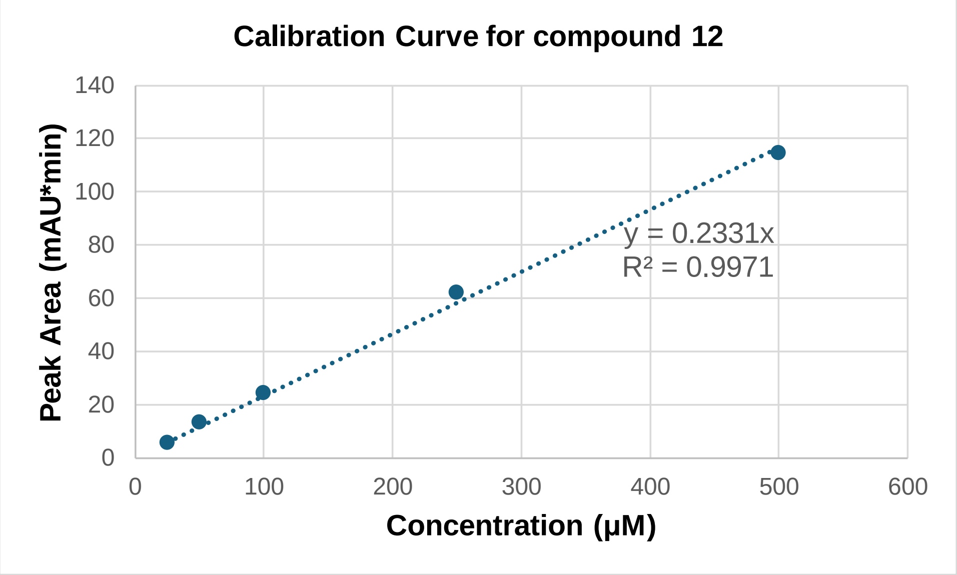

Mixture of methyl 3-ethyl-1,4-dimethyl-2-oxoindoline-3-carboxylate and methyl 3-ethyl-1,6-dimethyl-2-oxoindoline-3-carboxylate, 13

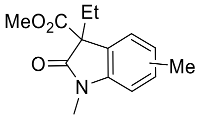

Compound **13** was obtained by chemical synthesis as a mixture of regioisomers methyl 3-ethyl-1,4-dimethyl-2-oxoindoline-3-carboxylate and methyl 3-ethyl-1,6-dimethyl-2-oxoindoline-3-carboxylate in ratio 2.7:1 respectively.

Methyl 3-ethyl-1,4-dimethyl-2-oxoindoline-3-carboxylate

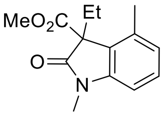

^{1}H NMR (500 MHz, $CDCl_3$): 7.21 (t, J = 7.8 Hz, 1H), 6.85 (d, J = 7.8 Hz, 1H), 6.70 (d, J = 7.7 Hz, 1H), 3.63 (s, 3H), 3.23 (s, 3H), 2.49 – 2.31 (m, 2H), 0.51 (t, J = 7.5Hz, 3H).

Methyl 3-ethyl-1,6-dimethyl-2-oxoindoline-3-carboxylate

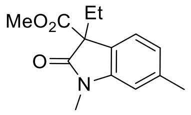

^{1}H NMR (500 MHz, $CDCl_3$): 7.12 (d, J = 7.6 Hz, 1H), 6.89 (d, J = 7.5 Hz,1H), 6.68 (d, J = 1.5 Hz, 1H), 3.64 (s, 3H), 3.23 (s, 3H), 2.39 (s, 3H), 2.34 – 2.17 (m, 2H), 0.63 (t, J = 7.4 Hz, 3H).

Calibration curve for mixture of regioisomers, 13

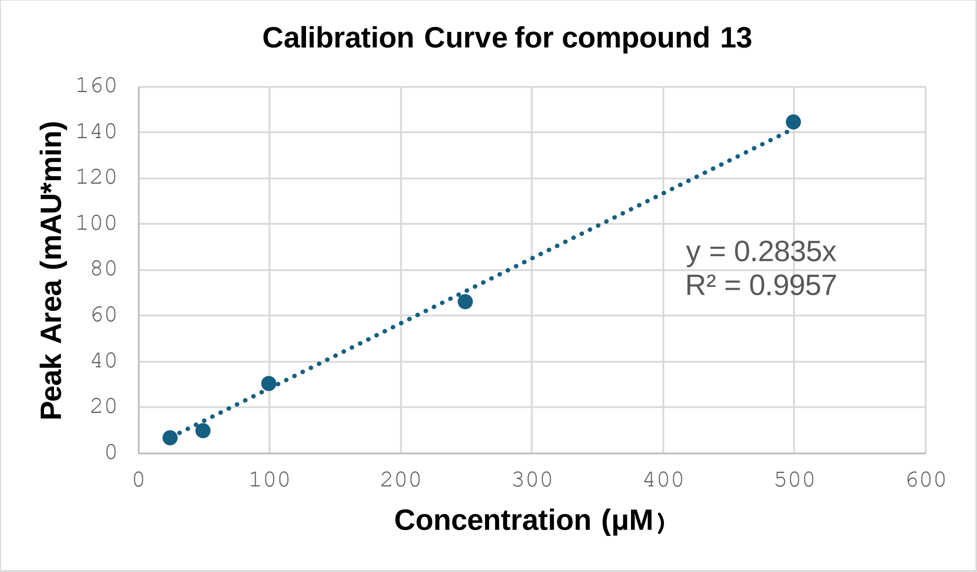

Methyl 1,3-dimethyl-2-oxoindoline-3-carboxylate, 14

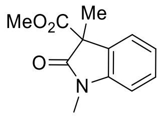

^{1}H NMR (400 MHz, $CDCl_3$): δ (ppm) **=** 7.35 (td, *J* = 7.8, 1.2 Hz, 1H), 7.31 – 7.25 (m, 1H), 7.09 (td, *J* = 7.6, 0.8 Hz, 1H), 6.89 (d, *J* = 7.8 Hz, 1H), 3.67 (s, 3H), 3.28 (s, 3H), 1.69 (s, 3H).

^{13}C NMR (101 MHz, $CDCl_3$): δ (ppm) **=** 175.1, 170.2, 143.6, 130.0, 129.0, 123.0, 122.9, 108.44, 54.9, 52.9, 26.5, 20.2.

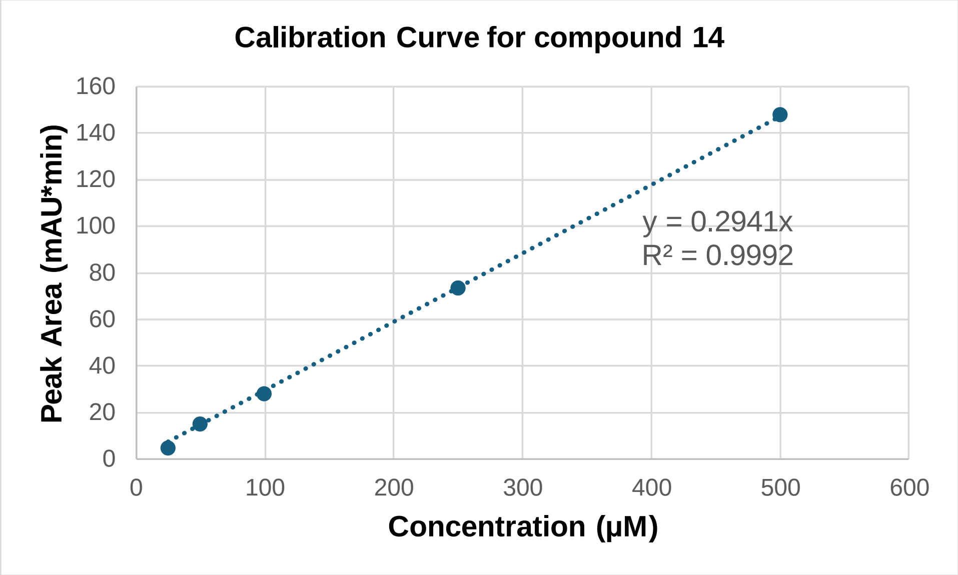

Methyl 3-benzyl-1-methyl-2-oxoindoline-3-carboxylate, 15

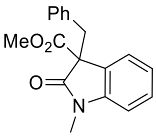

^{1}H NMR (400 MHz, $CDCl_3$): δ (ppm) **=** 7.35 – 7.30 (m, 1H), 7.22 (td, *J* = 7.8, 1.2 Hz, 1H), 7.08 (dd, *J* = 7.6, 0.9 Hz, 1H), 7.05 – 6.97 (m, 3H), 6.87 – 6.80 (m, 2H), 6.58 (d, *J* = 7.8 Hz, 1H), 3.71 (s, 3H), 3.55 (s, 2H), 2.95 (s, 3H).

^{13}C NMR (101 MHz, $CDCl_3$): δ (ppm) **=** 173.43, 169.78, 144.05, 134.34, 129.95, 129.08, 127.55, 127.31, 126.75, 123.95, 122.51, 108.16, 60.75, 53.04, 40.09, 26.12.

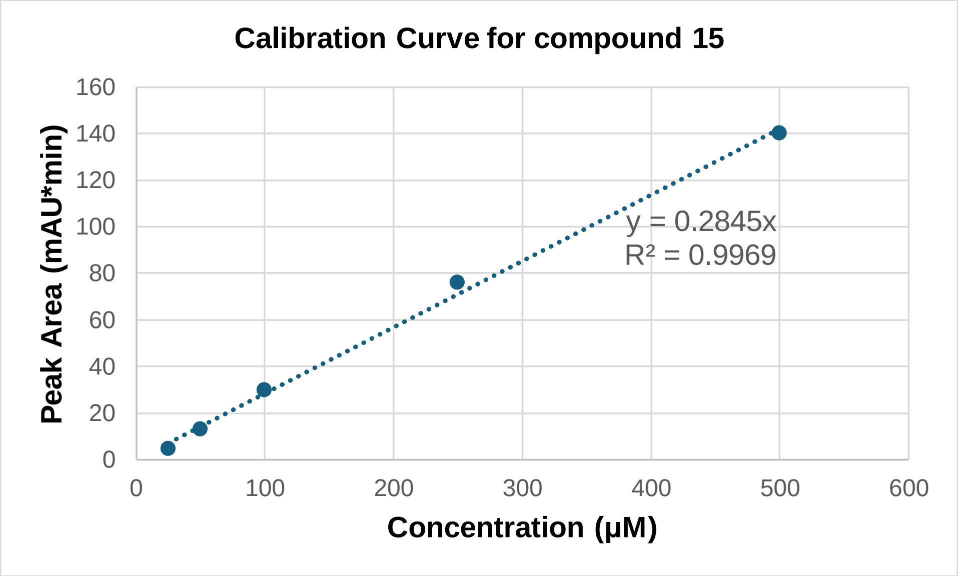

Methyl 1-methyl-2-oxo-3-phenylindoline-3-carboxylate, 16

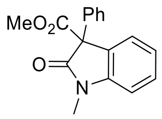

^{1}H NMR (400 MHz, $CDCl_3$): δ (ppm) **=** 7.41 – 7.31 (m, 2H), 7.25 (d, *J* = 6.2 Hz, 5H), 7.09 (td, *J* = 7.6, 0.9 Hz, 1H), 6.85 (d, *J* = 7.8 Hz, 1H), 3.68 (s, 3H), 3.16 (s, 3H).

^{13}C NMR (126 MHz, $CDCl_3$): δ (ppm) **=** 172.7, 169.7, 144.3, 135.7, 129.7, 128.5, 128.3, 127.8, 126.8, 126.0, 123.0, 108.7, 63.9, 53.4, 26.8.

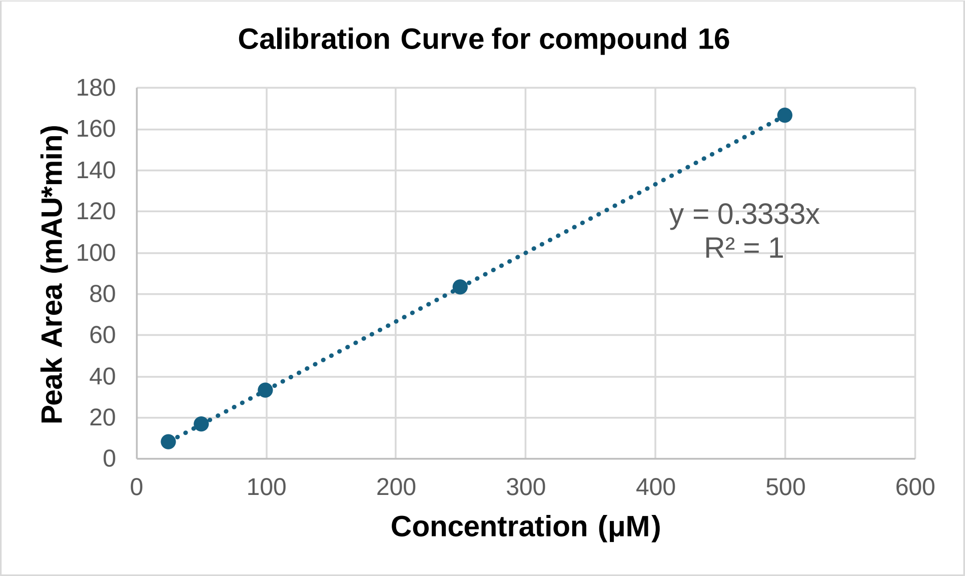

S6 Cyclic voltammetry (CV) of $PQQMe_3$

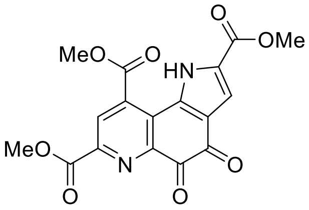

A CV of $PQQMe_3$ was first recorded over the whole potential range (Figure S4A). Four reduction waves (waves 1 to 4) and three oxidation waves (waves 5 to 7) were observed.

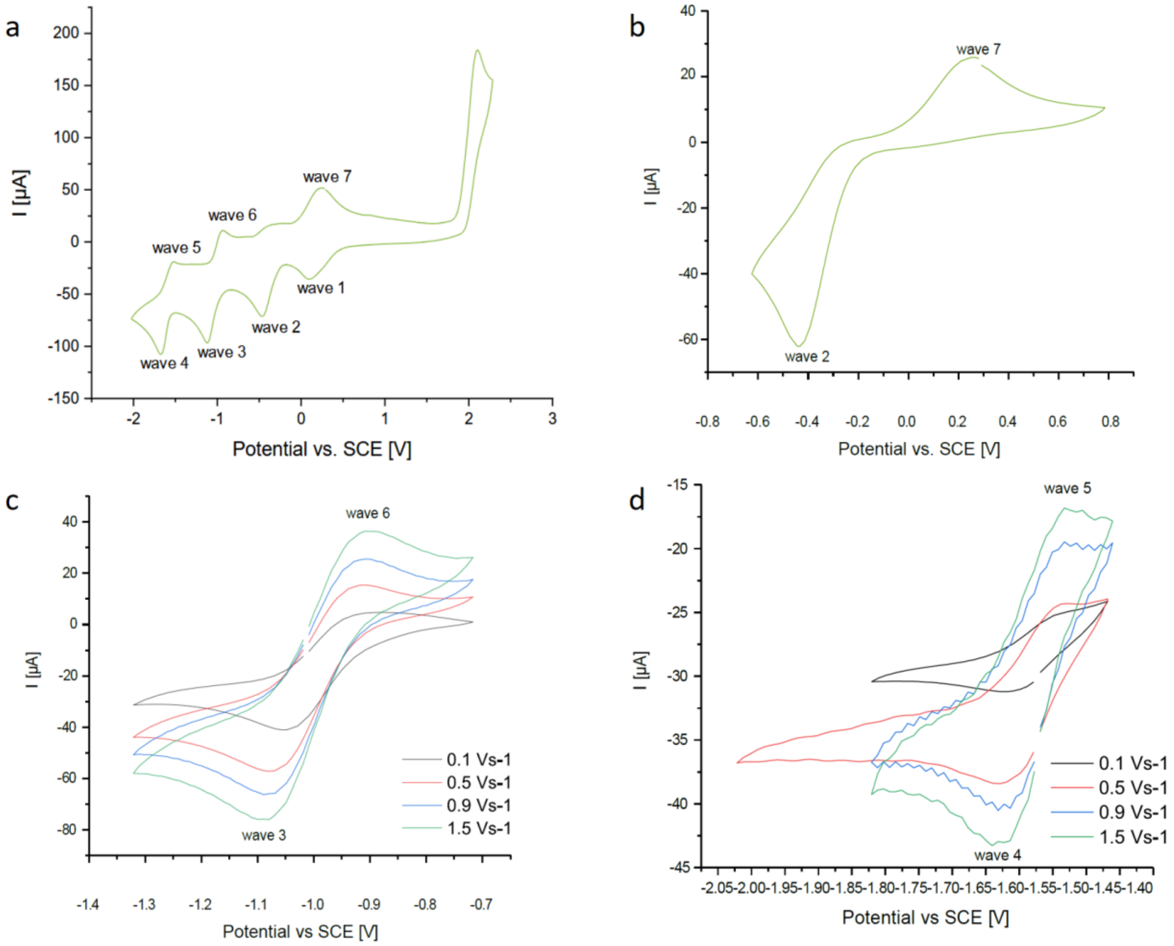

***Figure S4**: Cyclic voltammetry data of $PQQMe_3$*

We assigned wave 2 to the irreversible one-electron reduction of $PQQMe_3$ to radical anion $PQQMe_3^{\bullet-}$ with a peak potential of E_P = –0.43 V *vs.* SCE, which is in accordance with a study by *Fukuzumi* (E_P = –0.75 V *vs.* Fc^+/Fc; converted to –0.37 V *vs.* SCE).[12] No coupled reversible oxidation was observed for wave 2 (Figure S4B), and instead, oxidation occurred at wave 7 (E_P = 0.24 V *vs.* SCE). This observation indicates that $PQQMe_3^{\bullet-}$ is protonated to $PQQMe_3H^{\bullet}$ from either residual water in the solvent or another molecule of $PQQMe_3$. This analysis is supported by the *Fukuzumi* study, which reports disproportionation of $PQQMe_3H^{\bullet}$ to $PQQMe_3$ and $PQQMe_3H_2$ followed by oxidation of $PQQMe_3H_2$ at E_P = –0.18 V *vs.* Fc^+/Fc; converted to 0.20 V *vs.* SCE. At

more negative redox potentials, we observed reduction at wave 3 (E_P = –1.17 V *vs.* SCE) and oxidation at wave 6 (E_P = –1.00 V *vs.* SCE), which we also attempted to isolate (Figure S4C). The *Fukuzumi* study describes a total of four waves in this region, which are likely superimposed in our case. The reported redox processes include the irreversible reduction of $PQQMe_3^{\bullet-}$ to $PQQMe_3^{2-}$ at E_P = –1.25 V *vs.* Fc^+/Fc; converted to –0.87 V *vs.* SCE, a reversible couple at $E_{1/2}$ = –1.34 V *vs.* Fc^+/Fc; converted to –0.96 V *vs.* SCE assigned to the reduction of deprotonated $PQQMe_3$ ($PQQMe_3^-$) to radical anion $PQQMe_3^{\bullet 2-}$, and the irreversible oxidation of $PQQMe_3^{2-}$ to $PQQMe_3^{\bullet-}$ at E_P = –1.06 V *vs.* Fc^+/Fc; converted to –0.68 V *vs.* SCE (as a very small wave).[12]

At more negative potential, the scan-rate dependent measurements of wave 4 and wave 5 suggest a partially reversible couple (Figure S4D). Due to decomposition/ deposition over time, it was not possible to obtain clear voltammograms. The nature of this redox process was not studied further.

S7 Fluorescence spectroscopy

S7.1 PQQMe3 in organic solvent

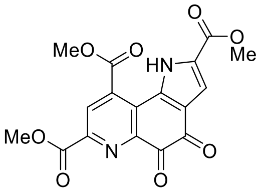

The absorption, emission and excitation spectra of PQQMe3 were measured. Here, two distinct fluorescent species were observed, emission bands can be seen in the right panel – one intense band in the 450 nm region, and a very weak band around 650 nm. Comparison of the excitation spectra (left panel) recorded in these emission bands with the UV-vis absorption shows that there are two distinct species in solution. The species emitting at 650 nm is the one dominating the UV-vis (i.e. it is at very much higher concentration), as evidenced by the essentially perfect agreement between excitation (red) and absorption (black) spectra.

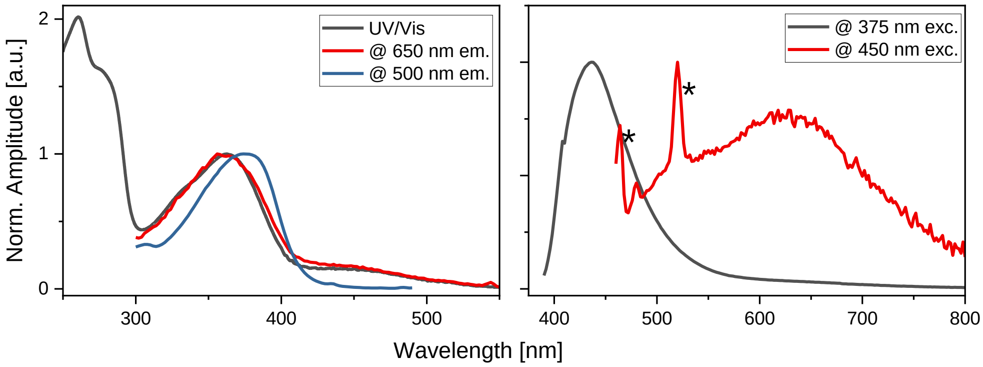

Figure S5*: Absorption and fluorescence spectra of PQQMe3 in DCM.*

S7.2 Free PQQ in aqueous solution

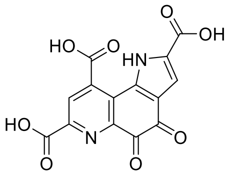

PQQ demonstrates pronounced absorption in the ultraviolet (UV) region, with wavelengths shorter than 400 nm and a less intense absorption band extending into the visible region reaching up to 600 nm. Fluorescence was measured upon excitation at different wavelengths. When excited at 375 nm, an emission was observed with a maximum of about 500 nm. No emission was observed when excited at higher wavelengths such as 450 nm.

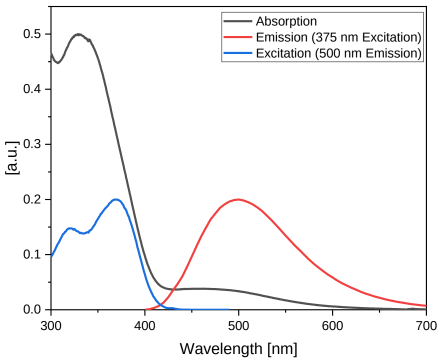

***Figure S6**: Absorption and fluorescence spectra of free PQQ.*

Next the dynamics of the fluorescence decay were investigated by time-correlated single-photon counting (TCSPC). The pulsed diode laser used for excitation had a wavelength of 375 nm to excite the main UV band. Excitation of the UV band (375 nm) yielded a single exponential decay with a lifetime of 1.21±0.01 ns, confirming population of a single emitting species.

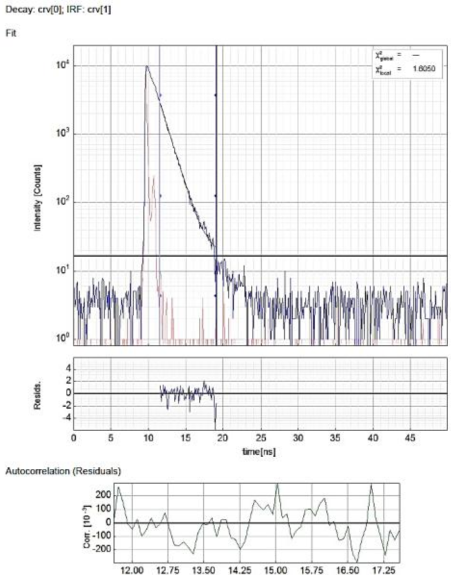

Parameter	Value	Δ	δ
A_1[kCnts/Chnl]	2.729	±0.023	0.8%
τ_1[ns]	1.2083	±0.0086	0.7%
I_1[kCnts]	33.76	±0.18	0.5%
$Bkgr_{Dec}$[kCnts]	0.0164	±0.0018	11%

S7.3 Aldose sugar DH from *E. coli* (YliI)

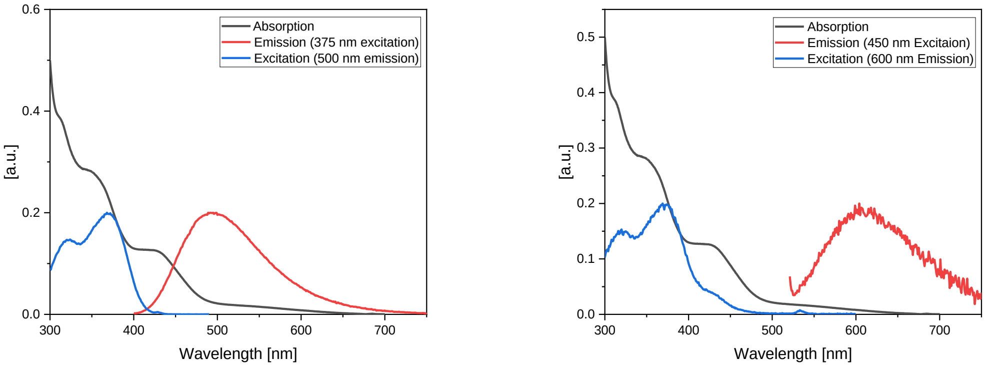

***Figure S7**: Absorption and fluorescence spectra of PQQ bound to the enzyme.*

The absorption, emission and excitation spectra of PQQ bound to aldose sugar DH from *E. coli* (YliI) were measured. Here, two distinct fluorescent species were observed, one with a maximum emission at 500 nm and the other one at 600 nm. The latter is present exclusively for protein-bound PQQ and is only accessible upon the excitation of a new absorption band around 420-450 nm.

Here, we determined lifetimes from TCSPC measurements upon excitation at 375 nm (intense emission) and 450 nm (weak emission). For both excitation wavelengths a double exponential decay was observed.

The excitation at 375 nm yields a double exponential decay with a lifetime of 1.186±0.020 ns (major) and 4.3±1.2 (minor).

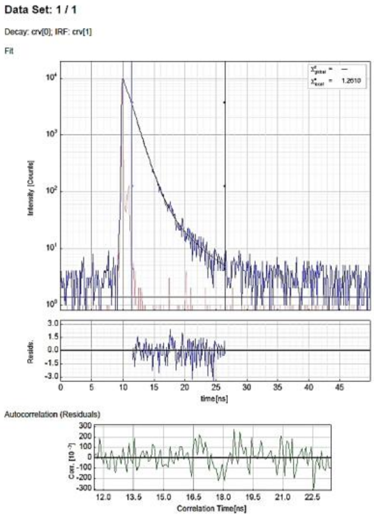

Parameter	Value	Δ	δ
A_1[kCnts/Chnl]	3.032	±0.034	1.1%
τ_1[ns]	1.186	±0.020	1.7%
I_1[kCnts]	36.79	±0.96	2.6%
A_2[kCnts/Chnl]	0.113	±0.039	35%
τ_2[ns]	4.3	±1.2	26%
I_2[kCnts]	4.94	±0.68	14%
$Bkgr_{Dec}$[kCnts]	0.0014	±0.0027	196%

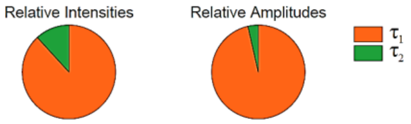

Excitation at 450 nm yields a double exponential decay with a lifetime of 0.79±0.24 ns (major) and 5.72±0.21 (minor).

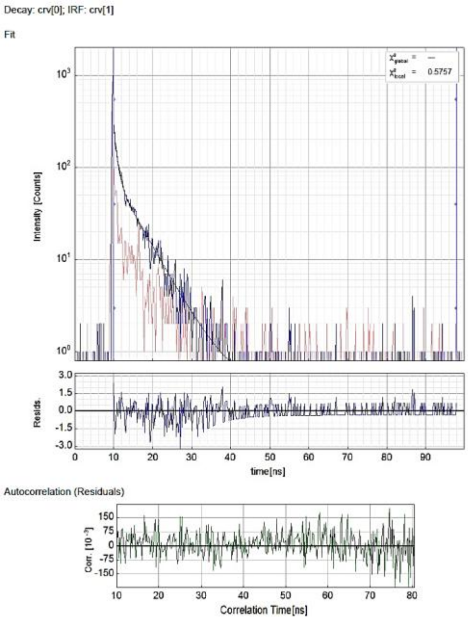

Parameter	Value	Δ	δ
A_1[kCnts/Chnl]	0.0784	±0.0046	5.9%
τ_1[ns]	5.72	±0.21	3.6%
I_1[kCnts]	2.292	±0.069	3.0%
A_2[kCnts/Chnl]	0.202	±0.048	24%
τ_2[ns]	0.79	±0.24	30%
I_2[kCnts]	0.809	±0.070	8.5%
$Bkgr_{Dec}$[kCnts]	0.0004	±0.0001	18%

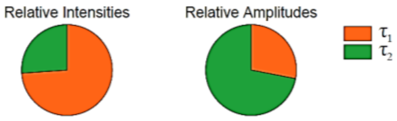

S8 Photocatalytic cyclizations with $PQQMe_3$ in organic solvent

S8.1 Standard procedure

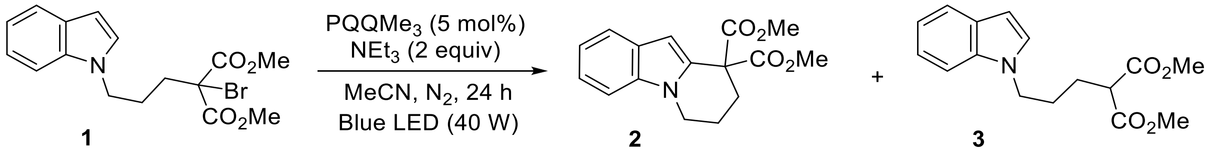

Under anaerobic conditions, an oven-dried reaction tube was equipped with a rubber septum and magnetic stir bar and was charged with $PQQMe_3$ (0.05 equiv), the corresponding halide (**1**, 0.10 mmol, 1.0 equiv), and Et_3N (0.20 mmol, 2.0 equiv) (2.0 mL) were added. The reaction tube was evacuated and backfilled with N_2 for three times and then 2.0 mL of dry MeCN was added via syringe. Then the reaction mixture was degassed again. Finally, the puncture hole in the septum was sealed with parafilm. The mixture was then placed at a distance of ~10 cm from a 427 nm Kessil lamp (PR160L-427nm, 40 W) for 24 hrs with fan cooling. After the completion of the reaction, the solvent was removed under reduced pressure. The residue is purified by chromatography on silica gel (19:1, hexane/EtOAc) to give **2**.

S8.2 Reaction optimization

Table S1. Solvent screening

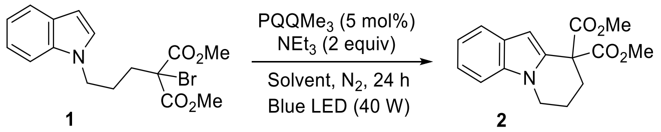

Entry[a]	Solvent	% Yield 2[b]
1	DCM	45
2	MeCN	*63*
3	MeOH	*22*
4	THF	55
5	DMF	40

[a] *Reaction condition*: Photocatalyst (5 mol%), **1** (1 equiv) and NEt_3 (2 equiv) in 2 mL solvent under N_2 atmosphere irradiated with a 40W 427 nm Kessil for 24 h. [b] Isolated yield.

Table S2. Reaction time screening

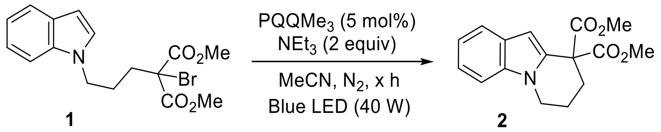

Entry[a]	Time (h)	% Yield 2[b]
1	2	13
2	6	*25*
3	12	*48*
4	16	63
5	24	79

[a] *Reaction condition*: Photocatalyst (5 mol%), **1** (1 equiv) and NEt_3 (2 equiv) in 2 mL MeCN under N_2 atmosphere irradiated with 40W 427 nm Kessil for x h. [b] Isolated yield.

Table S3. Catalyst loading

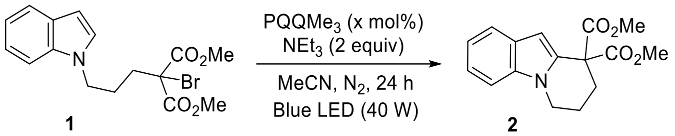

Entry[a]	Catalyst (mol%)	% Yield 2[b]
1	1	24
2	5	*79*
3	10	81

[a] *Reaction condition*: Photocatalyst (x mol%), **1** (1 equiv) and NEt_3 (2 equiv) in 2 mL MeCN under N_2 atmosphere irradiated with 40W 427 nm Kessil for 24 h. [b] Isolated yield.

Table S4. Sacrificial electron donor screening

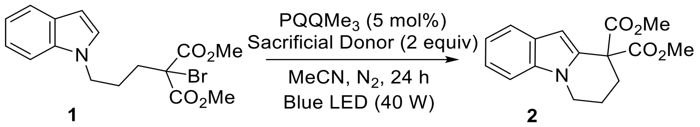

1 2

Entry[a]		$E_{1/2}$ (V vs. SCE)	% Yield 2[b]
1	NEt_3	0.83 V	79
2	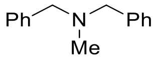	0.93 V	78
3	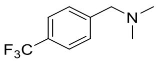	1.05 V	*81*
4	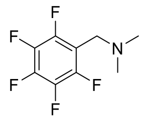	1.20 V	82
5	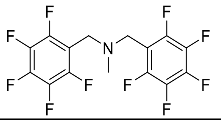	1.59 V	23

[a] *Reaction condition*: Photocatalyst (5 mol%), **1** (1 equiv) and sacrificial donor (2 equiv) in 2 mL MeCN under N_2 atmosphere irradiated with 40W 427 nm Kessil for 24 h. [b] Isolated yield.

Table S5. Wavelength screening

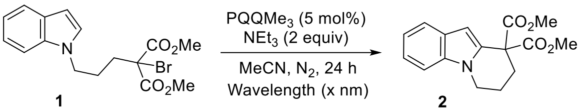

1 2

Entry[a]	Wavelength	% Yield 2[b]
1	Kessil, 427 nm, 40W	79
2	365 nm UV lamp	12
3	Blue LED (Kessil, TUNA BLUE, 40W)	37

[a] *Reaction condition*: Photocatalyst (5 mol%), **1** (1 equiv) and NEt_3 (2 equiv) in 2 mL MeCN under N_2 atmosphere irradiated with light source for 24 h. [b] Isolated yield.

S9 Mechanistic investigations with $PQQMe_3$

S9.1 Radical trapping experiments

Table S6. Radical trapping with TEMPO and 1,1-diphenylethylene (DPE)

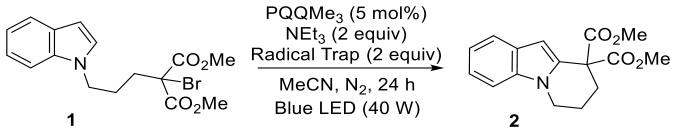

Entry	Additive	% Yield 2
1	TEMPO	0 (→ adduct observed)
2	DPE	<5 (→ adduct observed)

All the reactions have been performed according to the general procedure, additionally 2.0 equiv of radical trapping agent (TEMPO or 1,1-diphenylethylene (DPE)) was used. The reaction mixtures were analyzed by ^{1}H NMR and the adducts were identified with LC-MS.

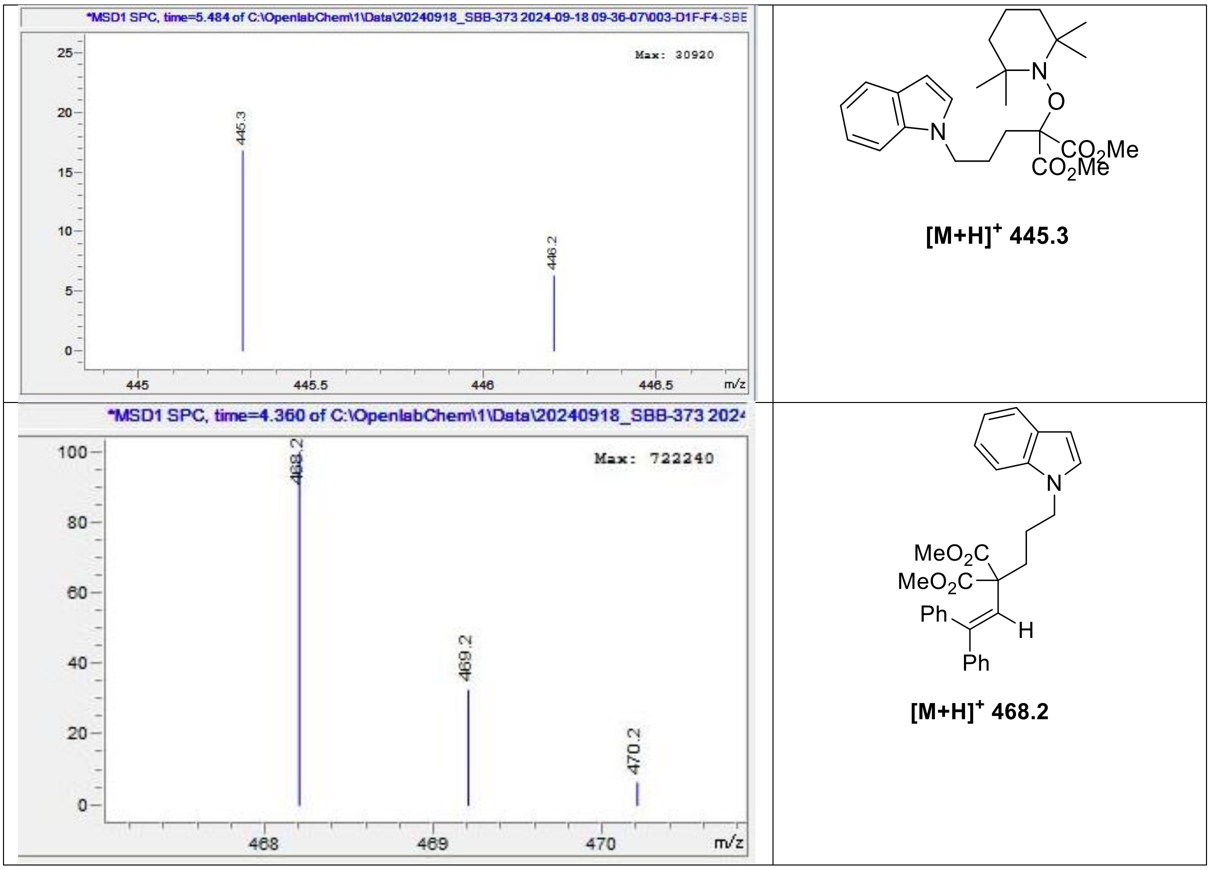

Figure S8: *Radical intermediate trapping with TEMPO and DPE detected by LC-MS.*

S9.2 Triplet quenching experiments

Table S7. Triplet quenching with butylated hydroxytoluene (BHT) and pyridazine

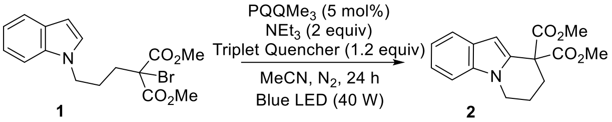

Entry	Additive	% Yield 2
1	BHT	0
2	pyridazine	<5

All the reactions have been performed according to the general procedure, additionally 1.2 equiv of triplet quencher (butylated hydroxytoluene (BHT) or pyridazine) was used. The reaction mixtures were analyzed by ^{1}H NMR.

S9.3 Light on/off experiment

The reaction was setup according to the general procedure in 0.6 mmol scale, mesitylene was used as an internal standard under N_2 atmosphere. The reaction mixture was sequentially stirred under visible light irradiation for 2 hours and dark for 2 hours. Every 2 hours, a 50 µl aliquot was taken from the reaction tube and analyzed by ^{1}H NMR and isolated yields were measured. After a total reaction time of 10 hours, the determined yields were plotted against the reaction time.

S9.4 Stern-Volmer fluorescence quenching studies with $PQQMe_3$

All the measurements were carried out mixing 20 µM solution of $PQQMe_3$ in dry degassed acetonitrile and increasing amount of quencher Et_3N in a 4 mL Schlenk cuvette. After each addition, emission spectra were recorded. Plots were drawn according to the Stern-Volmer equation. I_0 is the intensity without quencher, and I is the intensity with quencher. Change in fluorescence intensities are clear but we cannot distinguish clearly if the fluorescence quenching is due to shortened excited state lifetimes or due to photochemical reduction of $PQQMe_3$ happening simultaneously.

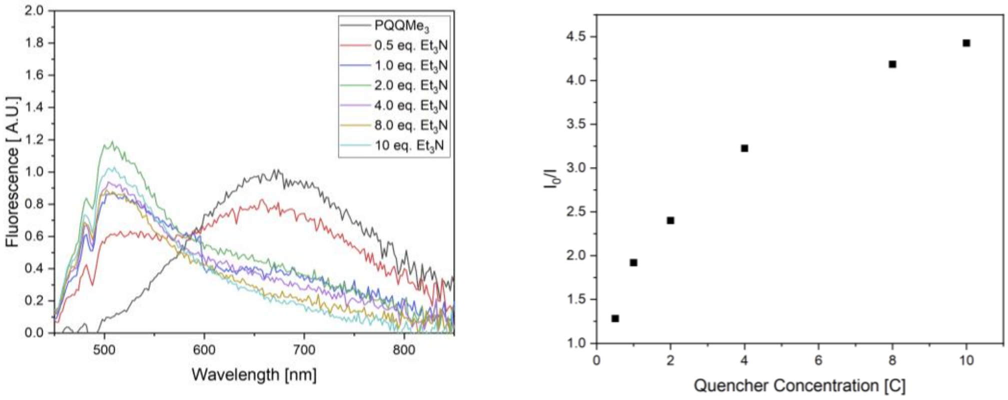

Figure S9: *Fluorescence quenching of $PQQMe_3$ (left) and Stern-Volmer plot (right).*

To further investigate the observed effects, the fluorescence lifetimes of $PQQMe_3$ (20 µM) in DCM solution and on addition of 400 µM NEt_3 after excitation at 375 nm and 450 nm were measured. The lifetime measured in the weak emission band around 650 nm (in black) is comparable or shorter than the instrument response (~100 ps, in blue). However, there were no significant changes in the singlet excited state lifetimes upon addition of NEt_3.

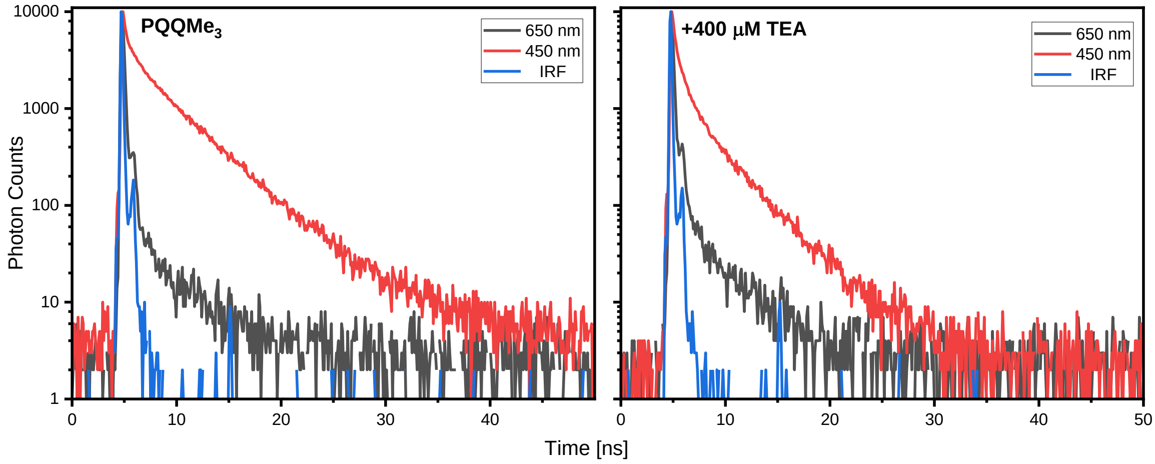

Figure S10: *Changes in fluorescence lifetimes of* $PQQMe_3$ *upon addition of* NEt_3*.*

S9.5 Photolysis experiment with $PQQMe_3$

A solution of $PQQMe_3$ (20 µM) and NEt_3 (20 µM) was prepared in DCM. The spectral changes due to the photoreduction of $PQQMe_3$ on addition of quencher NEt_3 in DCM under irradiation with LED Luxeon Z 420 nm with a wavelength range of 420-425 nm were measured on 20 s time intervals. The respective spectra are shown in the main text in Figure 2A.

S10 Photoenzymatic cyclizations with PQQ enzymes

S10.1 Redox-neutral cyclization of N-(bromoalkyl)-substituted indole

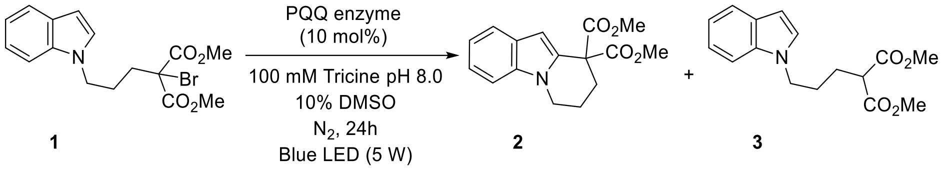

In the glove box, a GC vial was charged with tricine buffer (100 mM, pH 8.0) and PQQ enzyme (50 µM, 0.1 equiv) to reach a total volume of 225 µL. To this mixture was added N-(bromoalkyl)-substituted indole, **1** (500 µM, 1.0 equiv) in 25 µL DMSO to reach a final total volume of 250 µL. The reaction mixture was capped with a septum and sealed with parafilm. The vial was then taken out of the glovebox and was irradiated with the 6-vial photoreactor setup (455-460 nm, 5 W) for 24 hours. When the reaction time was completed, 350 µL MeCN was added to precipitate the protein. After short mixing of the sample, the denatured protein was separated by centrifugation (10 min, 12066 rcf, RT). Then the MeCN from supernatant was removed *in vacuo*. The reaction mixture was extracted with 350 µL EtOAc, evaporated to dryness in vacuo, redissolved in 350 µL MeOH, and injected on the HPLC to determine yield and chemoselectivity.

S10.2 Stereoselective redox-neutral cyclization of *α*-chloroamides

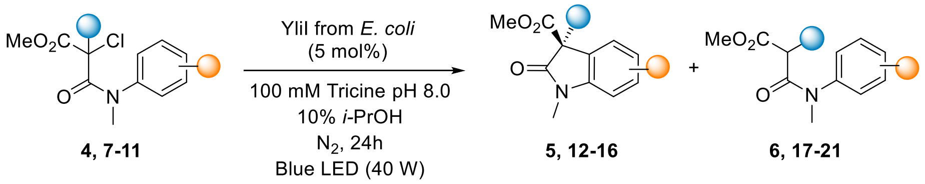

In the glove box, a GC vial was charged with tricine buffer (100 mM, pH 8.0) and PQQ enzyme (25 µM, 0.05 equiv) to reach a total volume of 225 µL. To this mixture was added chloroamide substrate (500 µM, 1.0 equiv) in 25 µL *i*-PrOH to reach a final total volume of 250 µL. The reaction mixture was capped with a septum and sealed with parafilm. The vial was then taken out of the glovebox placed in water bath and was irradiated with a A160WE TUNA BLUE 40 W Kessil lamp for 24 hours with fan cooling. When the reaction time was completed, 250 µL *i*-PrOH was added to precipitate the protein. After short mixing of the sample, the denatured protein was separated by centrifugated (10 min, 12066 rcf, RT). Then the *i*-PrOH from supernatant was removed *in vacuo*. The reaction mixture was extracted with 250 µL EtOAc, evaporated to dryness in vacuo, redissolved in 200-250 µL MeOH, and injected on the HPLC to determine yield and enantioselectivity using a chiral method.

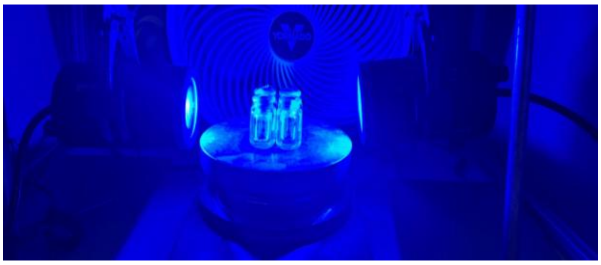

S10.3 Reaction optimization

Table S8. Evaluation of different wavelengths

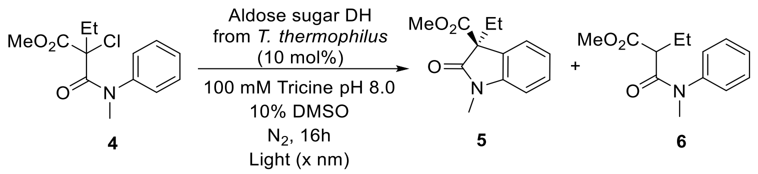

Wavelength	% yield 5	*e.r.*	% yield 6	*e.r.*
Blue LED (455-460 nm, 5W)	36	65:35	10	55:45
Cyan LED (480-485 nm, 3W)	21	72:28	5	70:30
Blue LED (410-420 nm, 3W)	8	65:35	2	-
Violet LED (Kessil, 390 nm, 40W)	68	52:48	15	50:50
Blue LED (Kessil, TUNA BLUE, 40W)	48	65:35	7	55:45

Note: We tested different lamp settings for both the chemical and enzymatic photoredox reactions. For PQQMe$_3$ reactions in organic solvent, we got the highest yields using the 427 nm Kessil lamp (PR160L-427nm, 40 W). In the enzymatic case, we first optimized the reaction conditions in small scale using a 6-vial photoreactor equipped with 455-460 nm 5W LEDs and then performed the final reactions for maximum yield with a Kessil lamp. Here, the model "A160WE TUNA BLUE, 40W" (max. at 465 nm) gave us an optimum of yield and stereoselectivity, most likely due to slightly less photodamage of the protein over time.

Table S9. Evaluation of organic co-solvents

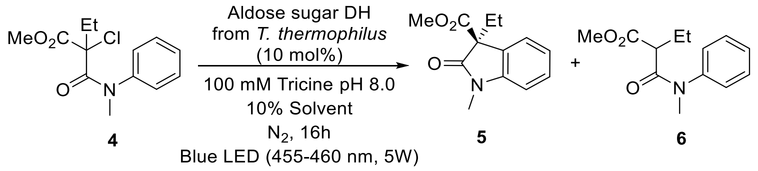

Solvent	% yield 5	*e.r.*	% yield 6	*e.r.*
DMSO	36	65:35	10	55:45
MeCN	26	65:35	10	70:30
i-PrOH	35	72:28	11	74:26

Table S10. Evaluation of substrate concentration

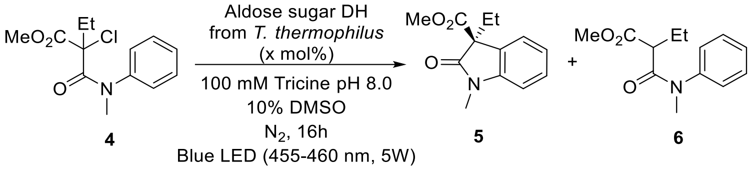

Catalyst Loading	[S]	% yield 5	*e.r.*	% yield 6	*e.r.*
10 mol%	500 µM	36	65:35	10	55:45
5 mol %	500 µM	17	76:24	4	80:20
2 mol%	500 µM	7	76:24	2	86:14
25 µM	1 mM	12	80:20	3	80:20
25 µM	5 mM	7	80:20	2	87:13

Table S11. Evaluation of reaction time

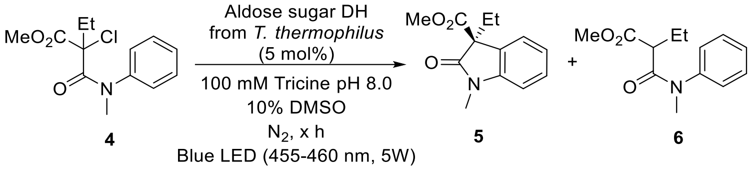

Time (h)	*e.r.* 4	% yield 5	*e.r. 5*	% yield 6	*e.r. 6*
1	50:50	4	88:12	0	-
2	50:50	7	88:12	1	95:5
3	50:50	10	88:12	2	92:8
4	50:50	14	88:12	3	76:23
16	50:50	19	80:20	4	76:23
24	50:50	26	80:20	5	76:23
48	50:50	32	70:30	8	57:43

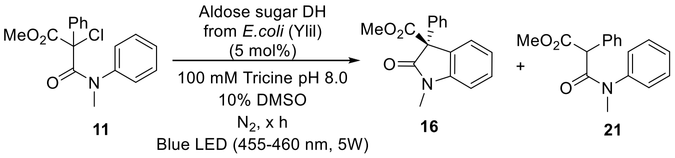

Time (h)	% yield 16	*e.r.* 16	% yield 21	*e.r.* 21
2	-	-	16	50:50
4	1	-	36	50:50
24	4	92:8	94	50:50

Table S12. Evaluation of pH

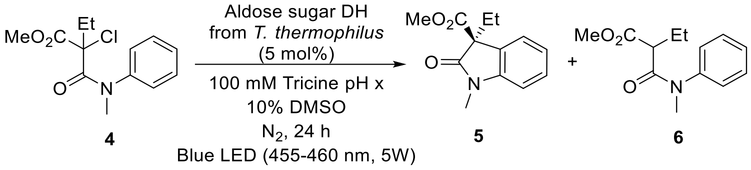

pH	% yield 5	e.r.	% yield 6	e.r.
7	16	80:20	3	70:30
7.5	22	80:20	4	70:30
8	27	80:20	5	70:30
8.5	21	80:20	4	70:30

Table S13. Enzyme screening under optimized conditions

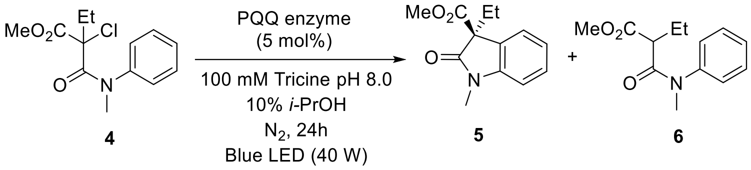

PQQ Enzyme	% yield 5	e.r.	% yield 6
Aldose sugar DH from *E. coli* (Ylil)	69	82:18	5
Aldose sugar DH from *T. thermophilus*	56	75:25	7
Aldose sugar DH from *A. calcoaceticus*	38	57:43	3
Alcohol DH from *P. putida* (PedH)	10	90:10	0

S11 Mechanistic investigations of photoenzymatic cyclizations

S 11.1 Light on/off experiment

In the glove box, a shell vial was charged with tricine buffer (100 mM, pH 8) and aldose sugar DH from *E. coli* (YliI) (25 µM, 0.05 equiv) to reach a total volume of 1.8 mL. To this mixture was added chloroamide substrate (**4**, 500 µM, 1.0 equiv.) in 200 µL *i*-PrOH to reach a final total volume of 2 mL. The reaction mixture was capped with a septum and sealed with parafilm. The vial was then taken out of the glovebox placed in water bath and was irradiated with a A160WE TUNA BLUE 40 W Kessil lamp for 1 hour. A sample of 250 µL was removed from the flask. The remaining reaction mixture was stored for 30 min in the dark and another sample was taken from the solution. This light on/off process was repeated 4 times.

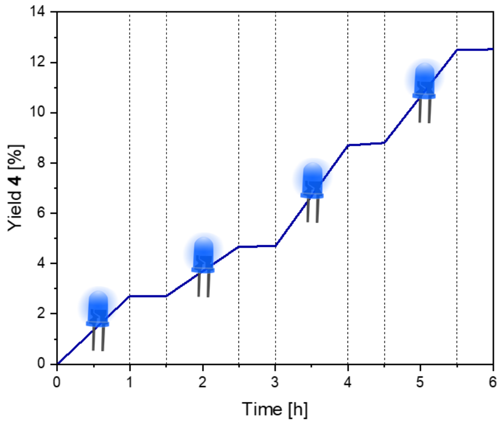

***Figure S11**: Light on/off experiment with aldose sugar DH from E. coli (YliI).*

S 11.2 Pre-irradiation experiment

This reaction was performed in accordance with the general procedure with slight modification. A GC vial was charged with a solution of YliI (25 µM) and tricine buffer (100 mM, pH 8) to a total volume of 225 µL inside the glove box. The sample was then taken out and was irradiated with a A160WE TUNA BLUE 40 W Kessil lamp for 30 min to photoreduce the enzyme and subsequently wrapped in aluminum foil. The vial was then brought into the glovebox. 25 µL of a 5 mM stock of chloroamide **4** in *i*-PrOH was added to vial. The resulting solution, still wrapped in foil, was stored for 2 hours in complete absence of light. Yields were calculated as described above.

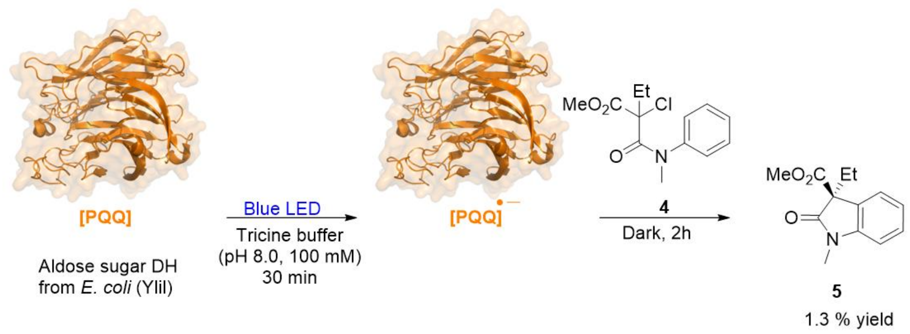

***Figure S12**: Photoreduction of aldose sugar DH from E. coli (YliI) and subsequent dark reaction.*

S 11.3 Triplet quenching

These reactions were performed in accordance with the general procedure with slight modification. A GC vial was charged with a solution of YliI (25 µM), tricine buffer (100 mM, pH 8), and triplet quencher (1.2 equiv) inside the glove box. To this mixture was added chloroamide substrate (**4**, 500 µM, 1.0 equiv.) in 25 µL *i*-PrOH to reach a final total volume of 250 µL. The sample was then taken out and was irradiated with a A160WE TUNA BLUE 40 W Kessil lamp for 24 hours. The yields were calculated as described above.

Table S14. Triplet quenching experiments with BHT and pyridazine

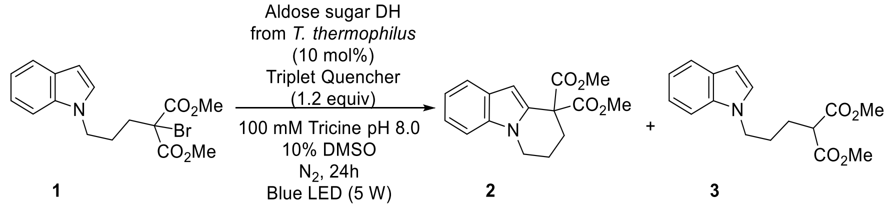

Additive	% yield 2	% yield 3
BHT	19	7
pyridazine	23	6

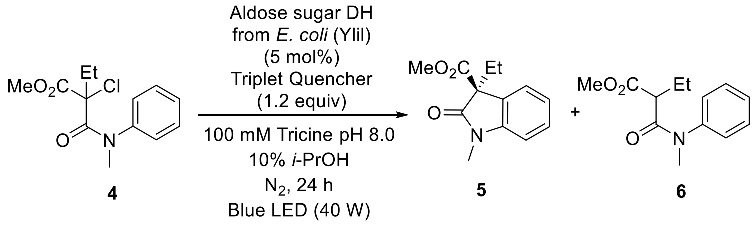

Additive	% yield 5	*e.r.*	% yield 6
BHT	18	80:20	4
pyridazine	21	80:20	5

S11.4 Stern-Volmer fluorescence quenching studies with aldose sugar DH from *E. coli* (YliI)

A sample with final volume of 4 mL with a concentration of 20 µM of aldose sugar DH from *E. coli* (YliI) was prepared in tricine buffer (100 mM, pH 8.0) and emission spectra were measured. Similar to PQQMe3, YliI shows dual emission dependent on excitation wavelength. Relatively strong emission after UV excitation, while a weak emission in the red appears after excitation around 450 nm.

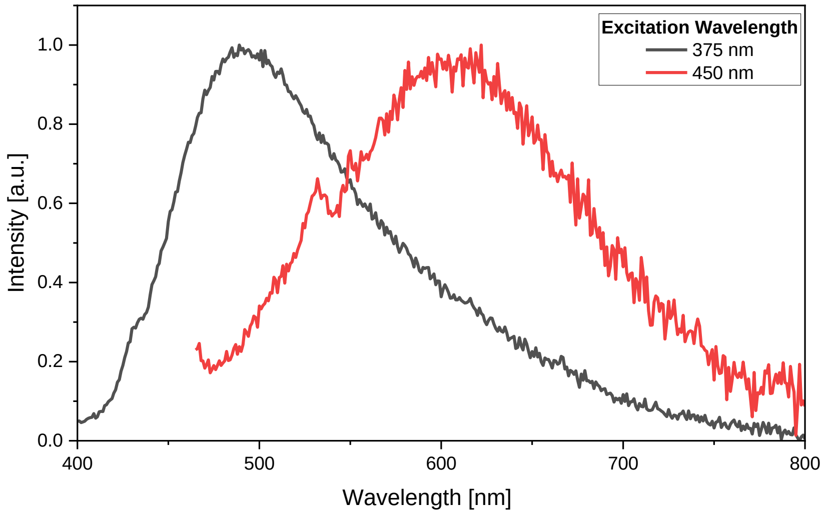

Figure S13: *Normalized emission spectra of aldose sugar DH from E. coli (YliI) in tricine buffer.*

The normalized absorption and excitation spectra of YliI were compared. The 650 nm emission clearly has an absorption spectrum agreeing essentially perfectly with the red shoulder of the absorption spectra. At shorter absorption wavelengths there may be more substantial contributions from the 500 nm emitter.

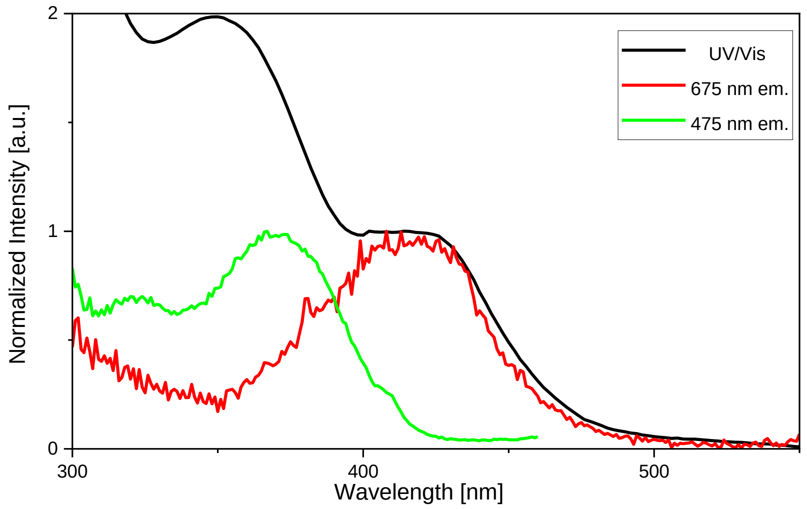

Figure S14: *Comparison of normalized absorption and excitation spectra of YliI.*

To further investigate, 20 μM solutions of YliI were then prepared in tricine buffer (100 mM, pH 8.0) and phosphate buffer KPi (100 mM, pH 8.0) and the emission spectra were measured for both cases. There was no evident difference. The ~10% difference is well within experimental uncertainties here, given the extreme weakness of the emission. In conclusion, the presence of sacrificial electron donor tricine doesn't seem to influence the fluorescence of YliI.

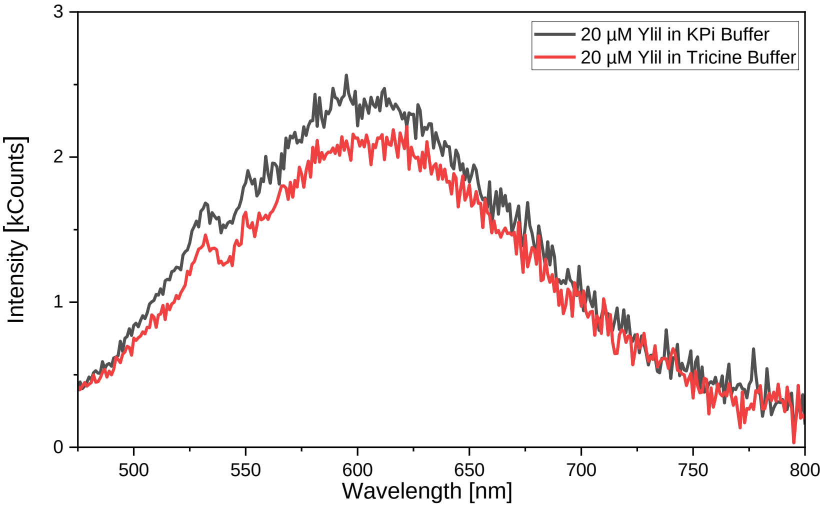

Figure S15: *Comparison of Ylil fluorescence after 450 nm excitation in reducing (tricine) and non-reducing (KPi) buffer solution.*

To observe potential changes in the fluorescence lifetimes of Ylil in the absence and presence of sacrificial electron donor, the lifetimes were measured in KPi and tricine buffer. In both the cases, after UV excitation, the total emission is dominated by the ~500 nm emission band. After 450 nm excitation, the weak and very quickly decaying $PQQMe_3$-like emission of Ylil can be detected in the red. There are no qualitative differences in the lifetimes relative to KPi buffer, as expected given the steady-state fluorescence.

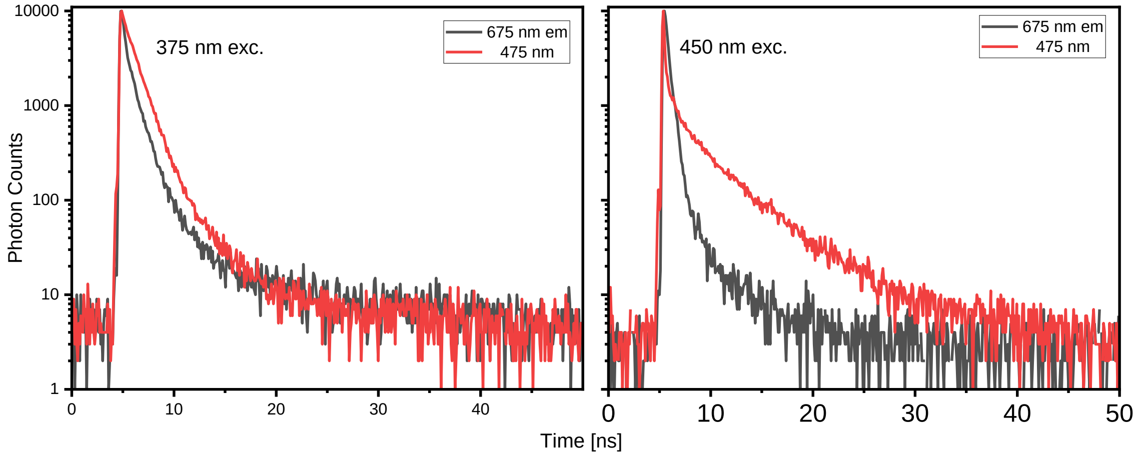

Figure S16: *Fluorescence lifetimes of Ylil in phosphate buffer after 375 nm excitation (left) and 450 nm excitation (right).*

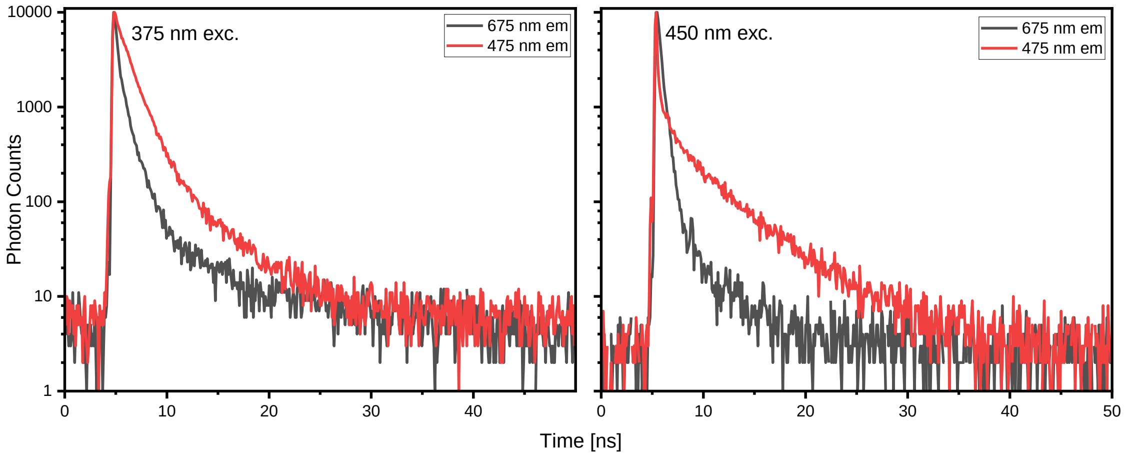

Figure S17: *Fluorescence lifetimes of YliI in tricine buffer after 375 nm excitation (left) and 450 nm excitation (right).*

The emission spectra and fluorescence lifetimes of reduced YliI (YliI in the presence of 10 mM glucose) were measured in the presence and absence of substrate **4**. It was observed that the substrate **4** does not noticeably influence the singlet state of reduced YliI.

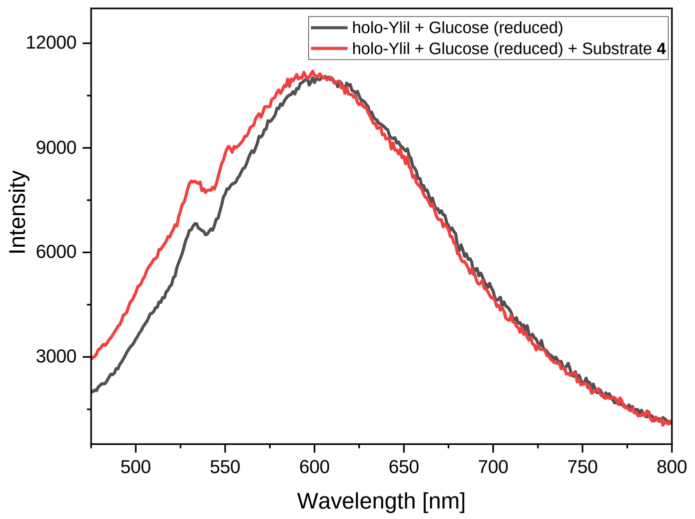

Figure S18: *Fluorescence spectra of reduced YliI in the presence and absence of substrate* ***4****. holo = reconstituted with PQQ and Ca(II)*

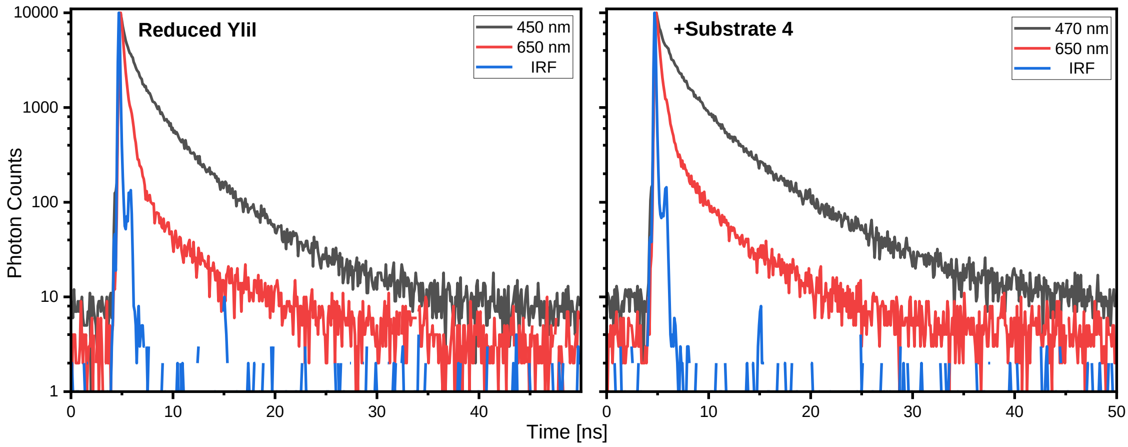

Figure S19: *Fluorescence lifetimes of reduced YliI in the presence and absence of substrate* ***4****.*

S11.5 Photolysis experiment with aldose sugar DH from *E. coli* (YliI)

A solution of aldose sugar DH from *E. coli* (YliI) (20 µM) was prepared in tricine buffer (100 mM, pH 8.0). The spectral changes due to the photoreduction of PQQ in the presence of sacrificial donor tricine under irradiation with LED Avonec 455-460 nm (5 W) were measured over time.

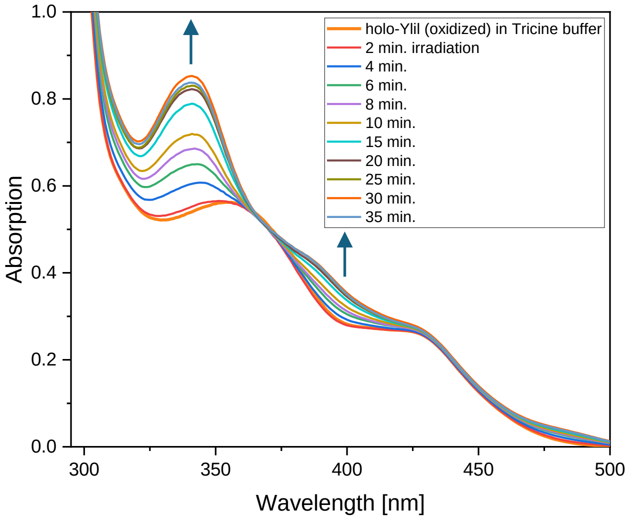

Figure S20: *Photolysis experiment with aldose sugar DH from E. coli (YliI) in tricine buffer. holo = reconstituted with PQQ and Ca(II)*

S11.6 Investigating the presence of potential charge transfer complexes

All samples were prepared in the glove box with an O_2 level lower than 0.1 ppm. Schlenk cuvettes were used to maintain oxygen free conditions during the duration of experiment. To observe a potential charge transfer (CT) complex in solution, a sample with final volume of 4 mL with a concentration of 20 µM of aldose sugar DH from *E. coli* (YliI) was prepared in tricine buffer (100 mM, pH 8.0) and a first absorption spectrum was recorded. The sample was brought back to glove box and 10 mM glucose was added to generate the fully reduced $PQQH_2$ state. Subsequently, 4 mg of substrate **4** (c = 3.7 mM) or substrate **1** (c = 2.7 mM) were added to the sample as a solution of 400 µL in *i*-PrOH. Following each step an absorption spectrum was taken.

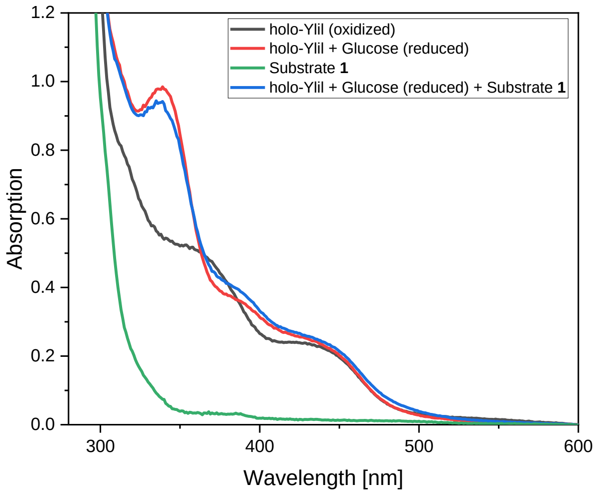

***Figure S21**: Absorption spectra of substrate **1** (green), aldose sugar DH from E. coli (YliI) in the oxidized form (black), reduced form generated with glucose (red), and reduced form in the presence of substrate **1** (blue). holo = reconstituted with PQQ and Ca(II)*

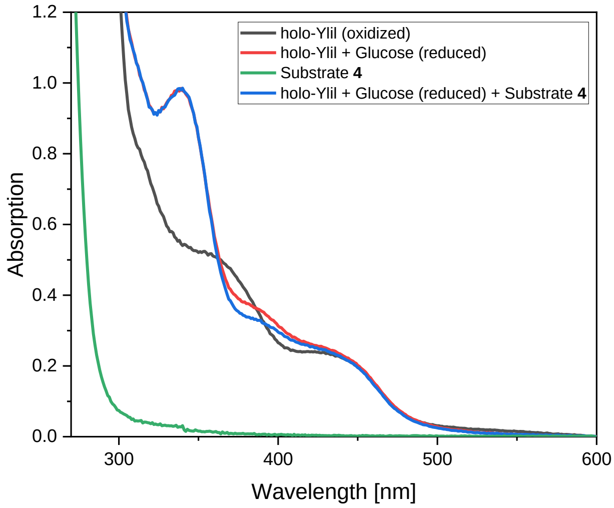

***Figure S22:** Absorption spectra of substrate **4** (green), aldose sugar DH from E. coli (YliI) in the oxidized form (black), reduced form generated with glucose (red), and reduced form in the presence of substrate **4** (blue). holo = reconstituted with PQQ and Ca(II)*

S12 Computational modeling and docking

As the available crystal structure of YliI from *E. coli* (PDB entry: 2G8S) did not contain the PQQ cofactor, the AI-based protein structure prediction tool Boltz-1[13] was used to model the holo-enzyme with Ca(II) and PQQ in the active site. This model was then superimposed back on the crystal structure to transfer the coordinates of metal and redox cofactor. The structures of product enantiomers *(S)*-**5** and *(R)*-**5** were calculated in Gaussian[14] and then subjected to targeted docking using the GNINA program,[15] which utilizes convolutional neural networks (CNN) for scoring. The number of binding modes to generate was set to 20, exhaustiveness to 32, and random seed to 0, all other parameters were set to default values. The flexible docking option was used to keep Tyr241, which is located in a loop near the PQQ cofactor, flexible during docking.

S13 HPLC data

S13.1 Photoenzymatic redox-neutral cyclization of N-(bromoalkyl)-substituted indole 1

Method for the separation of products **2** and **3**:
60% MeCN:Water, Column: Daicel, Chiralcel OD-RH, 150x4,6mm, 5µm

S13.1.1 Standard mixture of compound 2 and 3

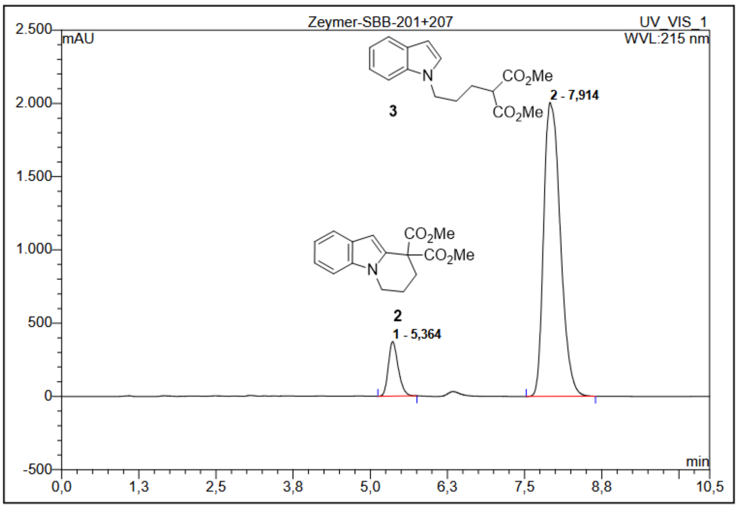

No.	Ret.Time min	Peak Name	Height mAU	Area mAU*min	Rel.Area %	Amount	Type
1	5,36	n.a.	372,651	69,315	9,91	n.a.	BMB
2	7,91	n.a.	2006,748	630,223	90,09	n.a.	BMB
Total:			2379,399	699,538	100,00	0,000	

S13.1.2 Reaction with aldose sugar DH from *E. coli* (YliI)

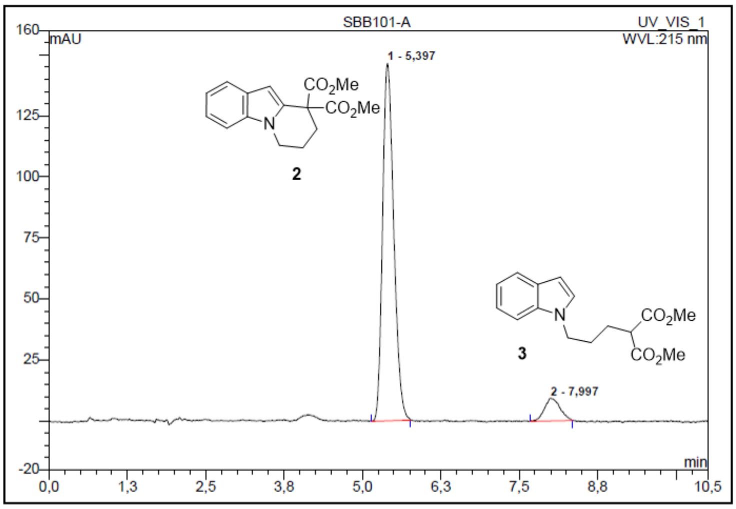

No.	Ret.Time min	Peak Name	Height mAU	Area mAU*min	Rel.Area %	Amount	Type
1	5,40	n.a.	146,494	30,179	91,85	n.a.	BMB
2	8,00	n.a.	9,302	2,679	8,15	n.a.	BMB*
Total:			155,796	32,858	100,00	0,000	

S13.1.3 Reaction with aldose sugar DH from *T. thermophilus*

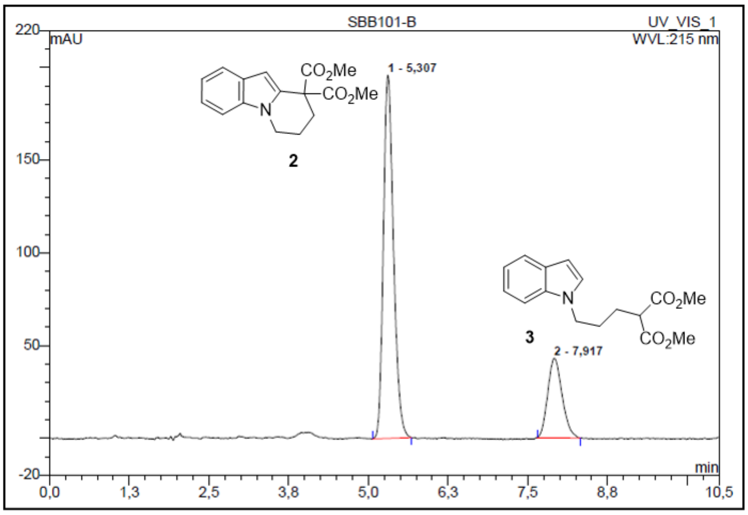

No.	Ret.Time min		Peak Name	Height mAU	Area mAU*min	Rel.Area %	Amount	Type
1	5,31	n.a.		195,971	36,058	76,28	n.a.	BMB
2	7,92	n.a.		42,947	11,213	23,72	n.a.	BMB
Total:				238,918	47,272	100,00	0,000	

S13.1.4 Reaction with aldose sugar DH from *A. calcoaceticus*

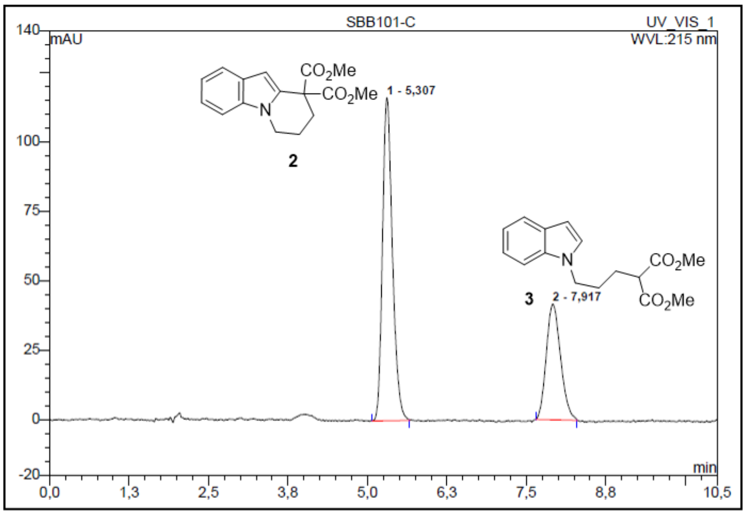

No.	Ret.Time min		Peak Name	Height mAU	Area mAU*min	Rel.Area %	Amount	Type
1	5,31	n.a.		116,207	21,099	66,01	n.a.	BMB
2	7,92	n.a.		41,667	10,866	33,99	n.a.	BMB
Total:				157,873	31,965	100,00	0,000	

S13.1.5 Reaction with alcohol DH from *P. putida* (PedH)

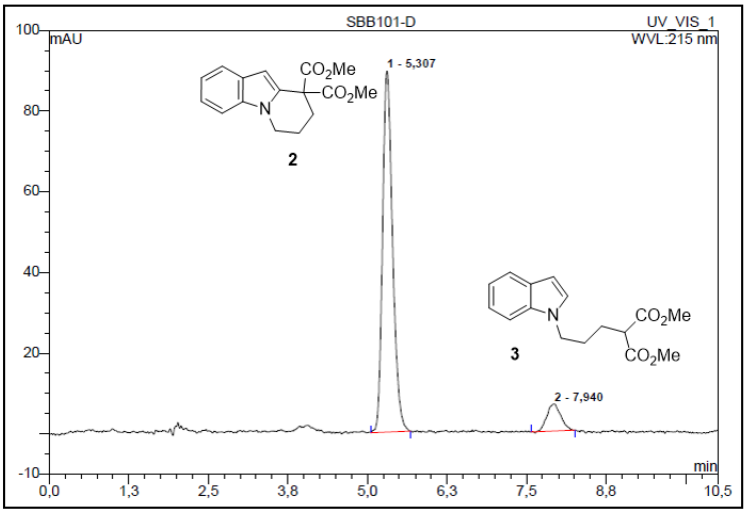

No.	Ret.Time min	Peak Name	Height mAU	Area mAU*min	Rel.Area %	Amount	Type
1	5,31	n.a.	89,466	16,348	90,63	n.a.	BMB
2	7,94	n.a.	6,714	1,691	9,37	n.a.	BMB*
Total:			96,180	18,040	100,00	0,000	

S13.1.6 Dark reaction with aldose sugar DH from *E. coli* (YliI))

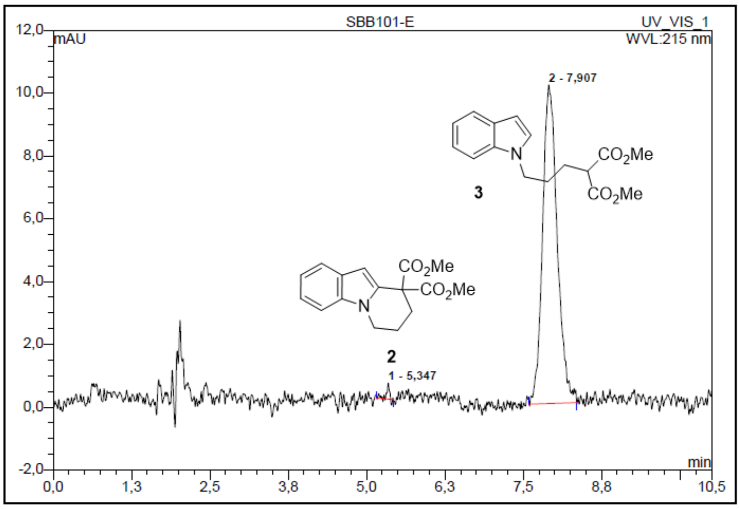

No.	Ret.Time min	Peak Name	Height mAU	Area mAU*min	Rel.Area %	Amount	Type
1	5,35	n.a.	0,511	0,024	0,85	n.a.	BMB*
2	7,91	n.a.	10,146	2,742	99,15	n.a.	BMB*
Total:			10,657	2,766	100,00	0,000	

S13.1.7 No enzyme

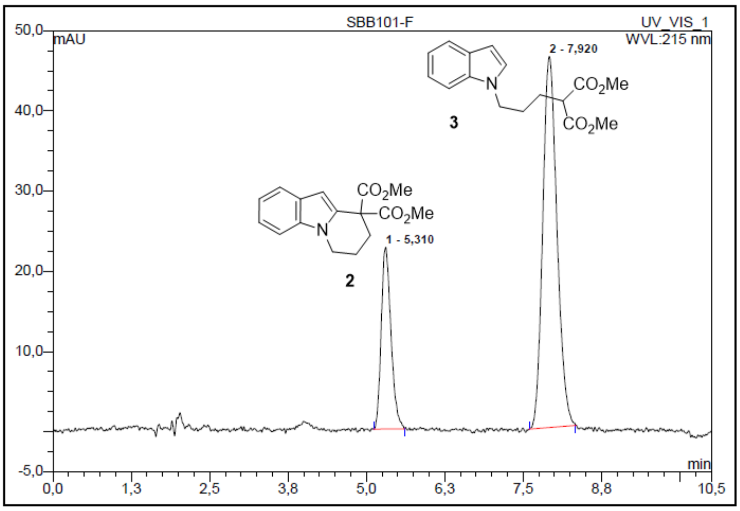

No.	Ret.Time min	Peak Name	Height mAU	Area mAU*min	Rel.Area %	Amount	Type
1	5,31	n.a.	22,648	4,073	25,05	n.a.	BMB*
2	7,92	n.a.	46,267	12,184	74,95	n.a.	BMB
Total:			68,914	16,257	100,00	0,000	

S13.2 Photoenzymatic stereoselective redox-neutral cyclizations of *α*-chloroamides

S13.2.1 Reactions with substrate 4: Chiral separation of enantiomers of products 5 and 6

Method: Column: OJ-RH and column temperature: 20 °C
Eluent A: Water, Eluent B: MeCN
Gradient: 20 % B to 50% B over 30 minutes

Methyl 3-ethyl-1-methyl-2-oxoindoline-3-carboxylate, 5 (Racemate)

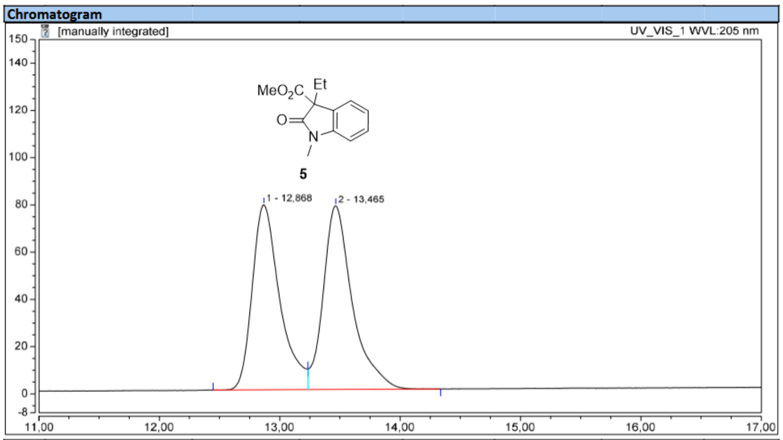

Integration Results

No.	Peak Name	Retention Time min	Area mAU*min	Height mAU	Relative Area %	Relative Height %	Amount n.a.
1		12,868	20,758	78,317	48,81	50,19	n.a.
2		13,465	21,770	77,733	51,19	49,81	n.a.
Total:			42,528	156,050	100,00	100,00	

Methyl 2-(methyl(phenyl)carbamoyl)butanoate, 6 (Racemate)

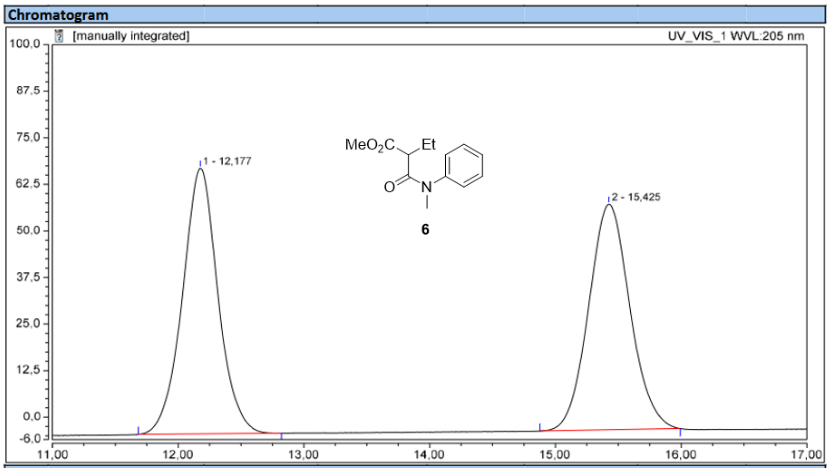

Integration Results

No.	Peak Name	Retention Time min	Area mAU*min	Height mAU	Relative Area %	Relative Height %	Amount n.a.
1		12,177	23,288	71,293	50,29	54,06	n.a.
2		15,425	23,017	60,580	49,71	45,94	n.a.
Total:			46,305	131,873	100,00	100,00	

Reaction with aldose sugar DH from *E. coli* (YliI)

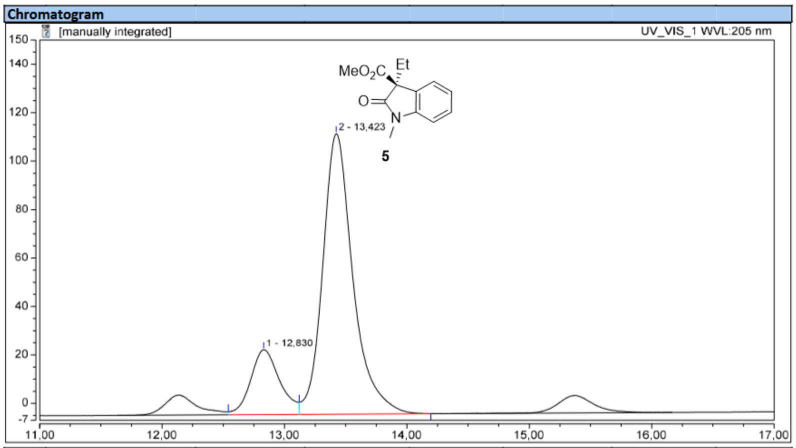

Integration Results

No.	Peak Name	Retention Time min	Area mAU*min	Height mAU	Relative Area %	Relative Height %	Amount n.a.
1		12,830	7,379	26,777	18,22	18,77	n.a.
2		13,423	33,114	115,844	81,78	81,23	n.a.
**Total:**			**40,494**	**142,621**	**100,00**	**100,00**	

Reaction with aldose sugar DH from *T. thermophilus*

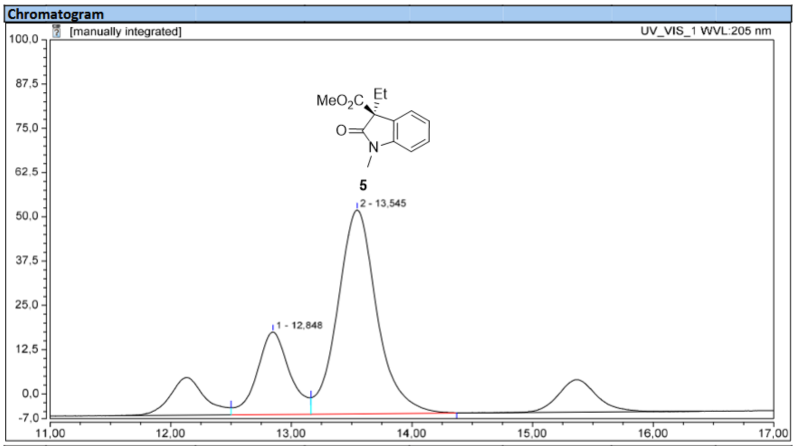

Integration Results

No.	Peak Name	Retention Time min	Area mAU*min	Height mAU	Relative Area %	Relative Height %	Amount n.a.
1		12,848	7,289	23,260	25,21	28,78	n.a.
2		13,545	21,626	57,555	74,79	71,22	n.a.
**Total:**			**28,915**	**80,816**	**100,00**	**100,00**	

Reaction with aldose sugar DH from *A. calcoaceticus*

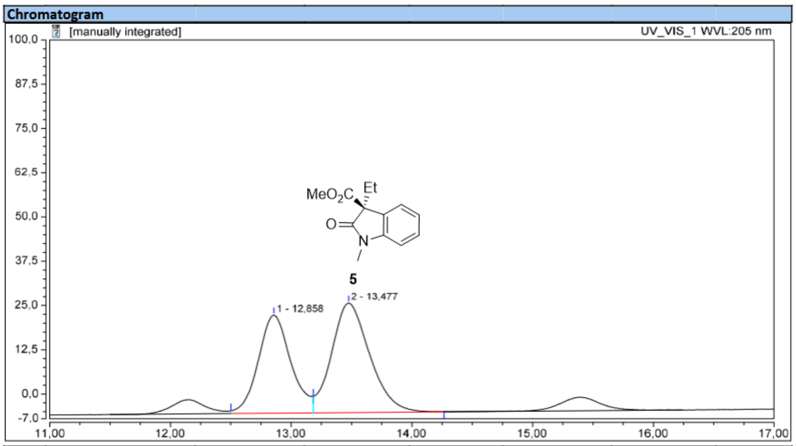

Integration Results

No.	Peak Name	Retention Time min	Area mAU*min	Height mAU	Relative Area %	Relative Height %	Amount n.a.
1		12,858	8,678	27,717	43,34	47,25	n.a.
2		13,477	11,343	30,938	56,66	52,75	n.a.
Total:			20,021	58,655	100,00	100,00	

Reaction with alcohol DH from *P. putida* (PedH)

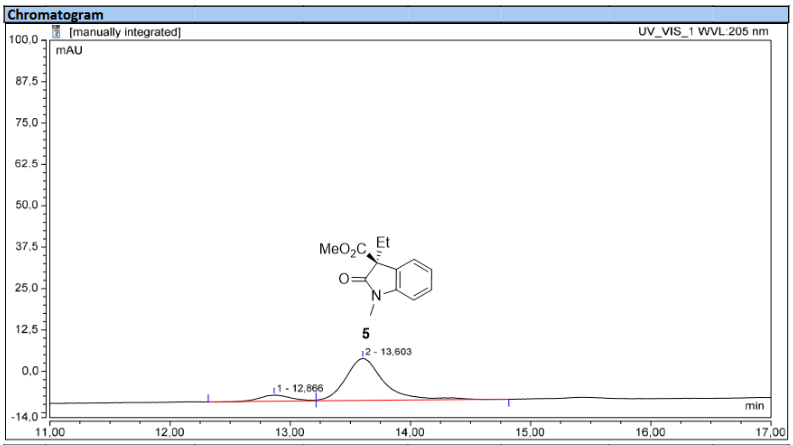

Integration Results

No.	Peak Name	Retention Time min	Area mAU*min	Height mAU	Relative Area %	Relative Height %	Amount n.a.
1		12,866	0,573	1,908	10,90	13,09	n.a.
2		13,603	4,683	12,668	89,10	86,91	n.a.
Total:			5,255	14,576	100,00	100,00	

Assignment of the absolute configuration of oxindole product 5

For the assignment of the absolute configuration, the racemic product standard of **5** and one of the reaction mixtures of substrate **4** with aldose sugar DH from *E. coli* (YliI) were run on the IC column (Method: Isocratic 10% IPA:Heptane, Column: IC, and, Column temperature: 20 °C). The same column was used in a previous study that had assigned the absolute configuration of **5** based on optical rotation compared to a literature reference. [16]

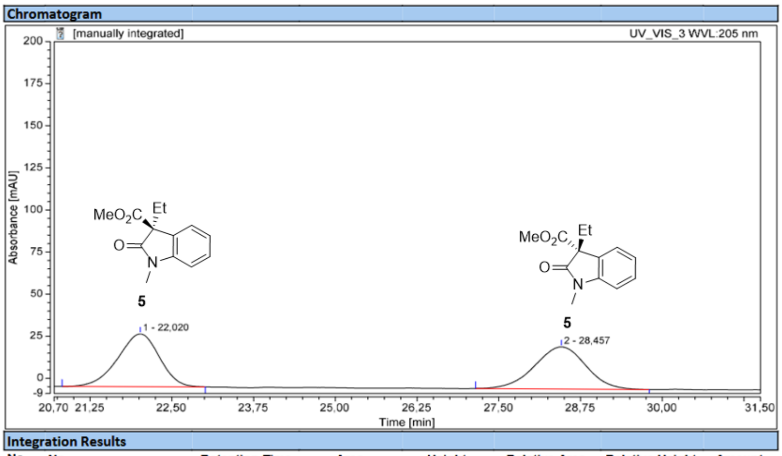

Integration Results

No.	No.	Retention Time min	Area mAU*min	Height mAU	Relative Area %	Relative Height %	Amount n.a.
1	1	22,020	23,142	31,336	50,42	55,66	n.a.
2	2	28,457	22,753	24,965	49,58	44,34	n.a.
**Total:**			**45,895**	**56,301**	**100,00**	**100,00**	

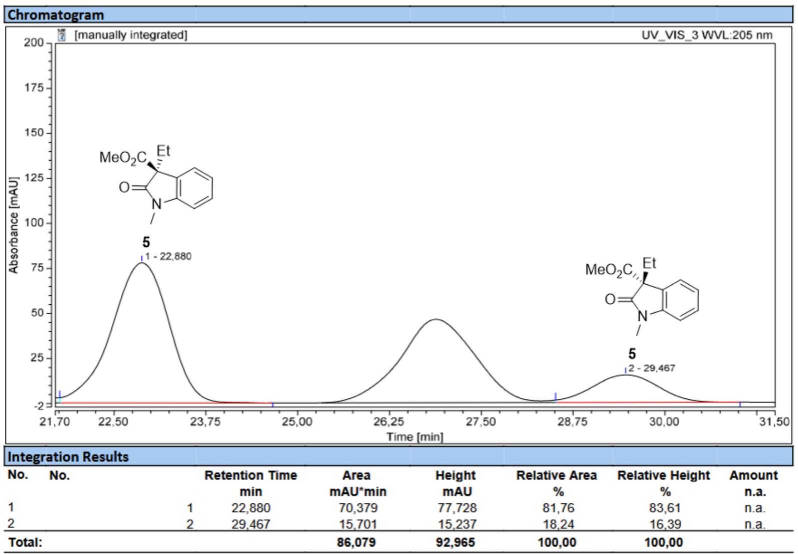

Integration Results

No.	No.	Retention Time min	Area mAU*min	Height mAU	Relative Area %	Relative Height %	Amount n.a.
1	1	22,880	70,379	77,728	81,76	83,61	n.a.
2	2	29,467	15,701	15,237	18,24	16,39	n.a.
**Total:**			**86,079**	**92,965**	**100,00**	**100,00**	

S13.2.2 Reaction with substrate 7: Chiral separation of enantiomers of product 12

Method: Isocratic 10% IPA:Heptane, Column: IC, and, Column temperature: 20 °C

Methyl 3-ethyl-5-methoxy-1-methyl-2-oxoindoline-3-carboxylate, 12 (Racemate)

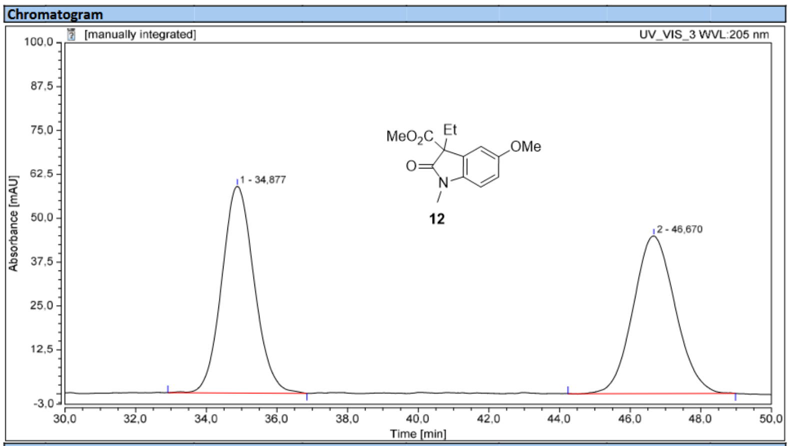

Integration Results

No.	No.	Retention Time min	Area mAU*min	Height mAU	Relative Area %	Relative Height %	Amount n.a.
1	1	34,877	62,582	58,881	50,31	56,74	n.a.
2	2	46,670	61,801	44,889	49,69	43,26	n.a.
Total:			124,382	103,770	100,00	100,00	

Reaction with aldose sugar DH from *E. coli* (YliI)

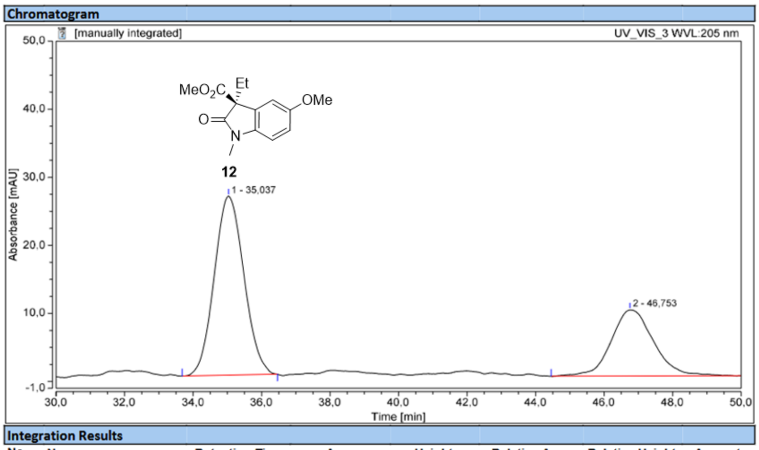

Integration Results

No.	No.	Retention Time min	Area mAU*min	Height mAU	Relative Area %	Relative Height %	Amount n.a.
1	1	35,037	26,402	26,298	65,40	72,98	n.a.
2	2	46,753	13,971	9,739	34,60	27,02	n.a.
Total:			40,373	36,037	100,00	100,00	

S13.2.3 Reaction with meta-substituted substrate 8: Chiral separation of regioisomers and their respective enantiomers of product 13

Method: Isocratic 10% IPA:Heptane, Column: IC, and, Column temperature: 20 °C

*<u>Note:</u> The product standard of **13** was obtained racemically but at 2.7:1 r.r. by chemical synthesis. We could thus assign the regioisomer peaks in the chromatogram from the NMR analysis of that mixture of major and minor product. In contrast, the enzymatic reaction showed no regioselectivity (1:1 r.r.) but pronounced enantioselectivity for both product regioisomers.*

Methyl 3-ethyl-1,4-dimethyl-2-oxoindoline-3-carboxylate (Racemate)

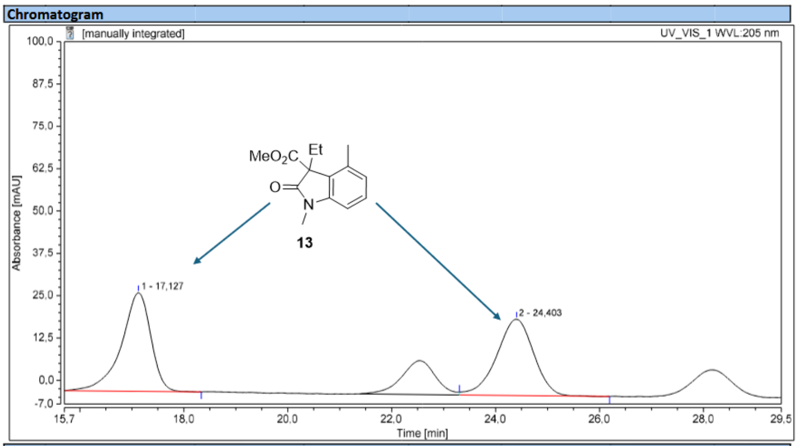

Integration Results

No.	No.	Retention Time min	Area mAU*min	Height mAU	Relative Area %	Relative Height %	Amount n.a.
1	1	17,127	19,156	29,119	50,87	56,38	n.a.
2	2	24,403	18,499	22,526	49,13	43,62	n.a.
Total:			37,655	51,645	100,00	100,00	

Reaction with aldose sugar DH from *E. coli* (YliI)

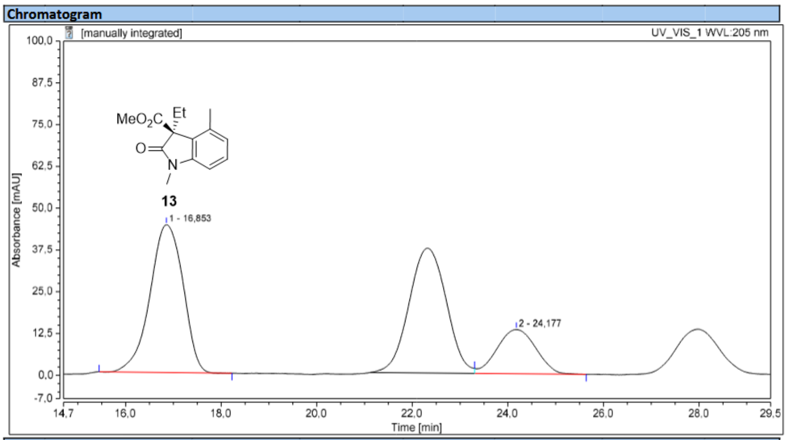

Integration Results

No.	No.	Retention Time min	Area mAU*min	Height mAU	Relative Area %	Relative Height %	Amount n.a.
1	1	16,853	35,501	44,240	74,02	76,99	n.a.
2	2	24,177	12,460	13,224	25,98	23,01	n.a.
Total:			47,960	57,464	100,00	100,00	

Methyl 3-ethyl-1,6-dimethyl-2-oxoindoline-3-carboxylate (Racemate)

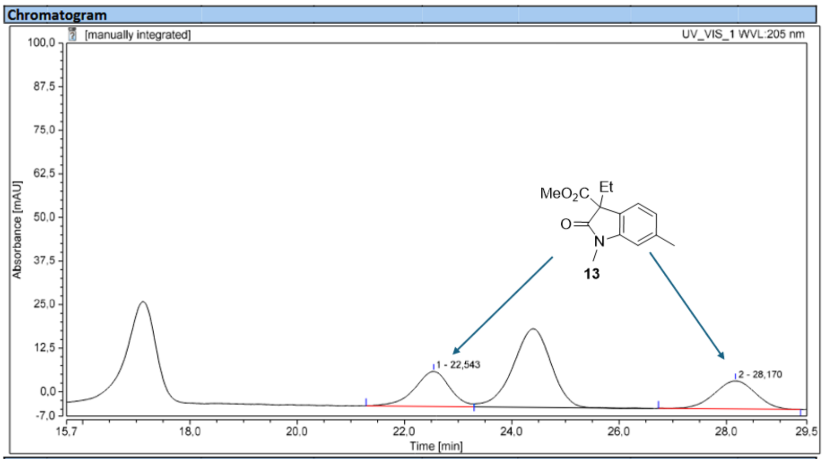

Integration Results

No.	No.	Retention Time min	Area mAU*min	Height mAU	Relative Area %	Relative Height %	Amount n.a.
1	1	22,543	7,642	10,041	51,68	55,61	n.a.
2	2	28,170	7,145	8,016	48,32	44,39	n.a.
**Total:**			**14,787**	**18,057**	**100,00**	**100,00**	

Reaction with aldose sugar DH from *E. coli* (YliI)

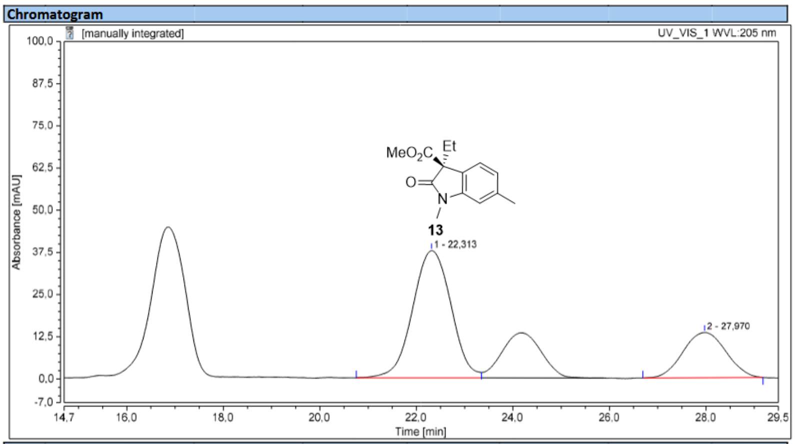

Integration Results

No.	No.	Retention Time min	Area mAU*min	Height mAU	Relative Area %	Relative Height %	Amount n.a.
1	1	22,313	34,613	37,715	71,45	73,77	n.a.
2	2	27,970	13,828	13,409	28,55	26,23	n.a.
**Total:**			**48,440**	**51,124**	**100,00**	**100,00**	

S13.2.4 Reaction with substrate 9: Chiral separation of enantiomers of product 14

Method: Isocratic 10% IPA:Heptane, Column: IC, and, Column temperature: 20 °C

Methyl 1,3-dimethyl-2-oxoindoline-3-carboxylate, 14 (Racemate)

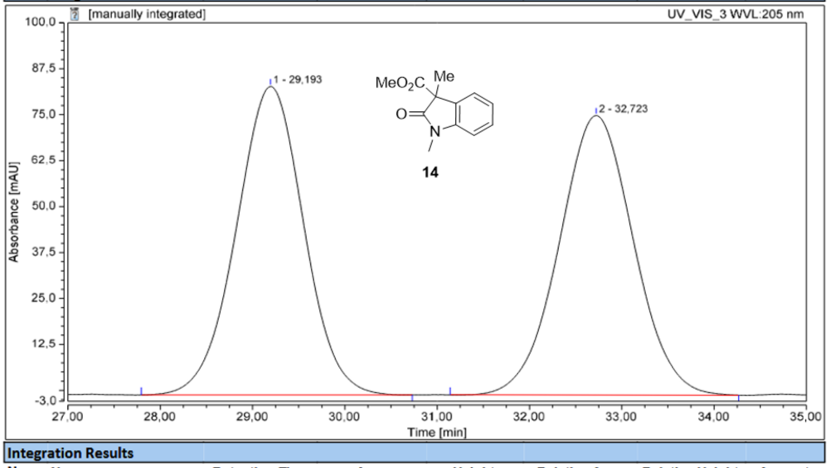

Integration Results

No.	No.	Retention Time min	Area mAU*min	Height mAU	Relative Area %	Relative Height %	Amount n.a.
1	1	29,193	71,065	83,910	50,06	52,47	n.a.
2	2	32,723	70,904	76,013	49,94	47,53	n.a.
Total:			141,969	159,923	100,00	100,00	

Reaction with aldose sugar DH from *E. coli* (YliI)

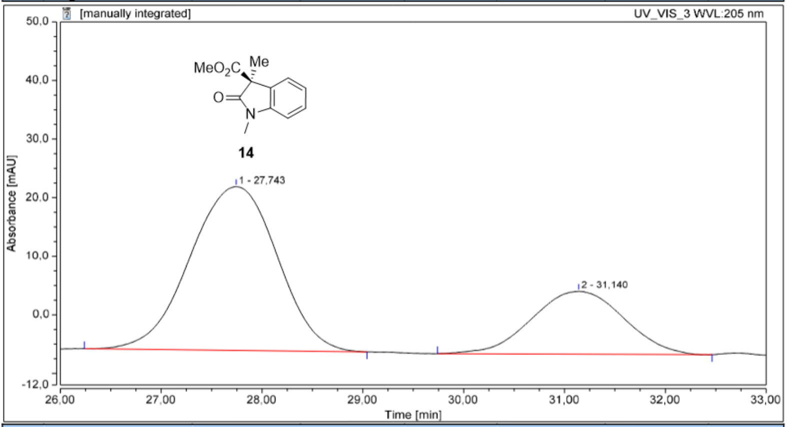

Integration Results

No.	No.	Retention Time min	Area mAU*min	Height mAU	Relative Area %	Relative Height %	Amount n.a.
1	1	27,743	28,064	27,950	71,25	72,29	n.a.
2	2	31,140	11,327	10,715	28,75	27,71	n.a.
Total:			39,391	38,665	100,00	100,00	

S13.2.4 Reaction with substrate 10: Chiral separation of enantiomers of product 15

Method: Isocratic 20% IPA:Heptane, Column: IC, and, Column temperature: 40 °C

Methyl 3-benzyl-1-methyl-2-oxoindoline-3-carboxylate, 15 (Racemate)

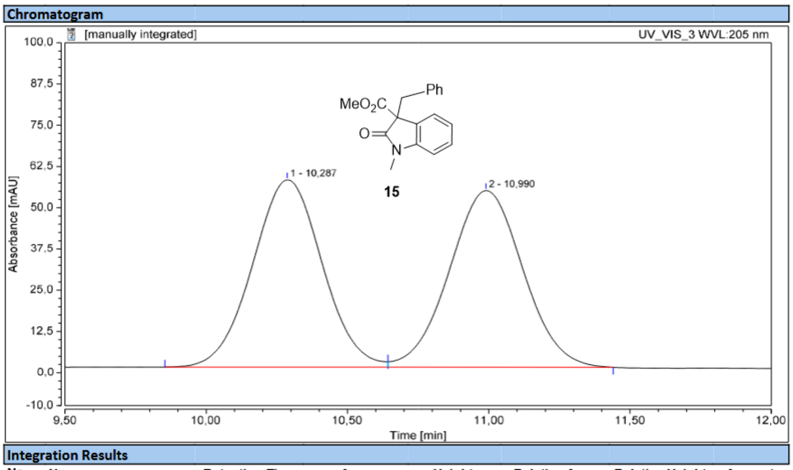

Integration Results

No.	No.	Retention Time min	Area mAU*min	Height mAU	Relative Area %	Relative Height %	Amount
1	1	10,287	16,231	56,762	50,05	51,46	n.a.
2	2	10,990	16,196	53,546	49,95	48,54	n.a.
Total:			32,427	110,309	100,00	100,00	

Reaction with aldose sugar DH from *E. coli* (YliI)

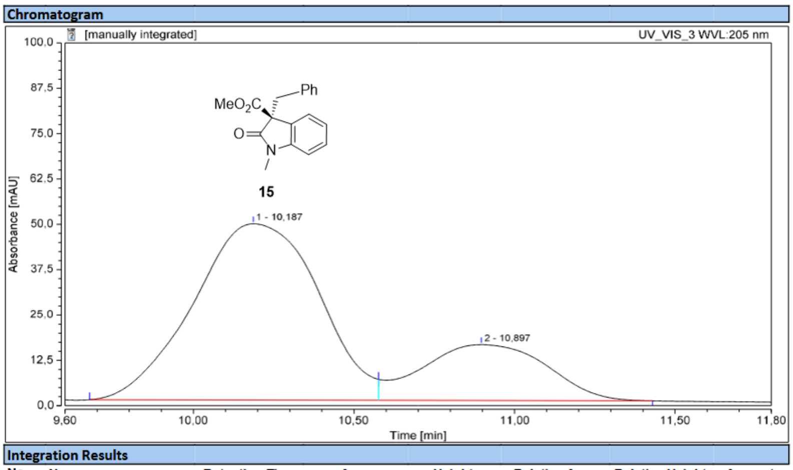

Integration Results

No.	No.	Retention Time min	Area mAU*min	Height mAU	Relative Area %	Relative Height %	Amount
1	1	10,187	21,943	48,594	75,89	75,95	n.a.
2	2	10,897	6,970	15,391	24,11	24,05	n.a.
Total:			28,912	63,985	100,00	100,00	

S13.2.5 Reaction with substrate 11: Chiral separation of enantiomers of products 16 and 21

Method: Isocratic 20% IPA:Heptane, Column: IC, and, Column temperature: 40 °C

Methyl 1-methyl-2-oxo-3-phenylindoline-3-carboxylate, 16 (Racemate)

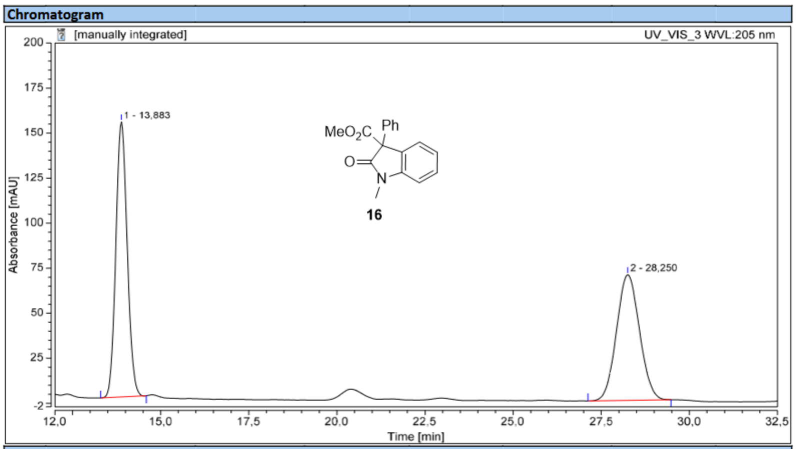

Integration Results

No.	No.	Retention Time min	Area mAU*min	Height mAU	Relative Area %	Relative Height %	Amount n.a.
1	1	13,883	56,936	152,779	51,95	68,63	n.a.
2	2	28,250	52,667	69,840	48,05	31,37	n.a.
**Total:**			**109,604**	**222,619**	**100,00**	**100,00**	

Methyl 3-(methyl(phenyl)amino)-3-oxo-2-phenylpropanoate, 21 (Racemate)

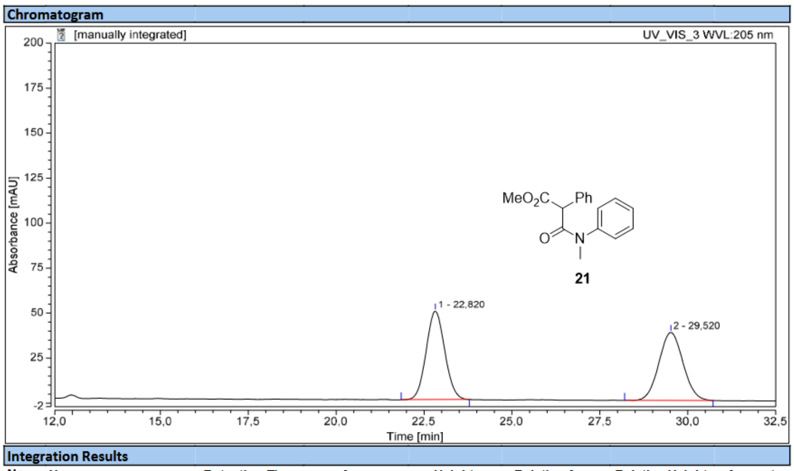

Integration Results

No.	No.	Retention Time min	Area mAU*min	Height mAU	Relative Area %	Relative Height %	Amount n.a.
1	1	22,820	30,216	49,127	50,15	56,53	n.a.
2	2	29,520	30,037	37,778	49,85	43,47	n.a.
**Total:**			**60,254**	**86,905**	**100,00**	**100,00**	

Reaction with Aldose sugar DH from *E. coli* (YliI)

Chromatogram

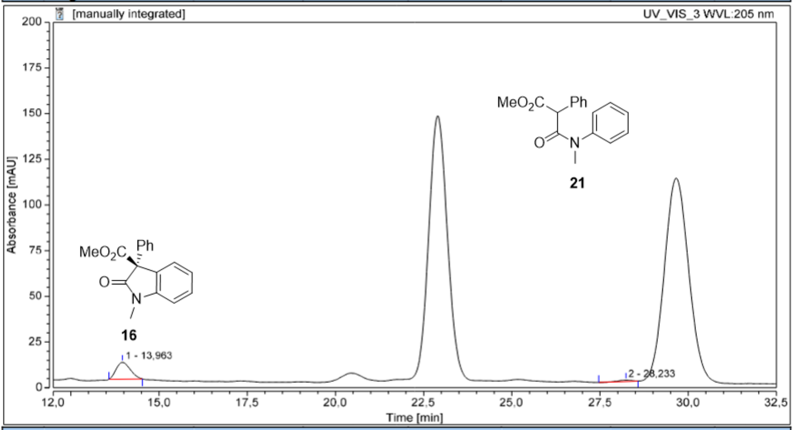

Integration Results

No.	No.	Retention Time min	Area mAU*min	Height mAU	Relative Area %	Relative Height %	Amount n.a.
1	1	13,963	4,248	9,229	92,48	92,71	n.a.
2	2	28,233	0,345	0,726	7,52	7,29	n.a.
**Total:**			**4,593**	**9,955**	**100,00**	**100,00**	

S14 NMR spectra

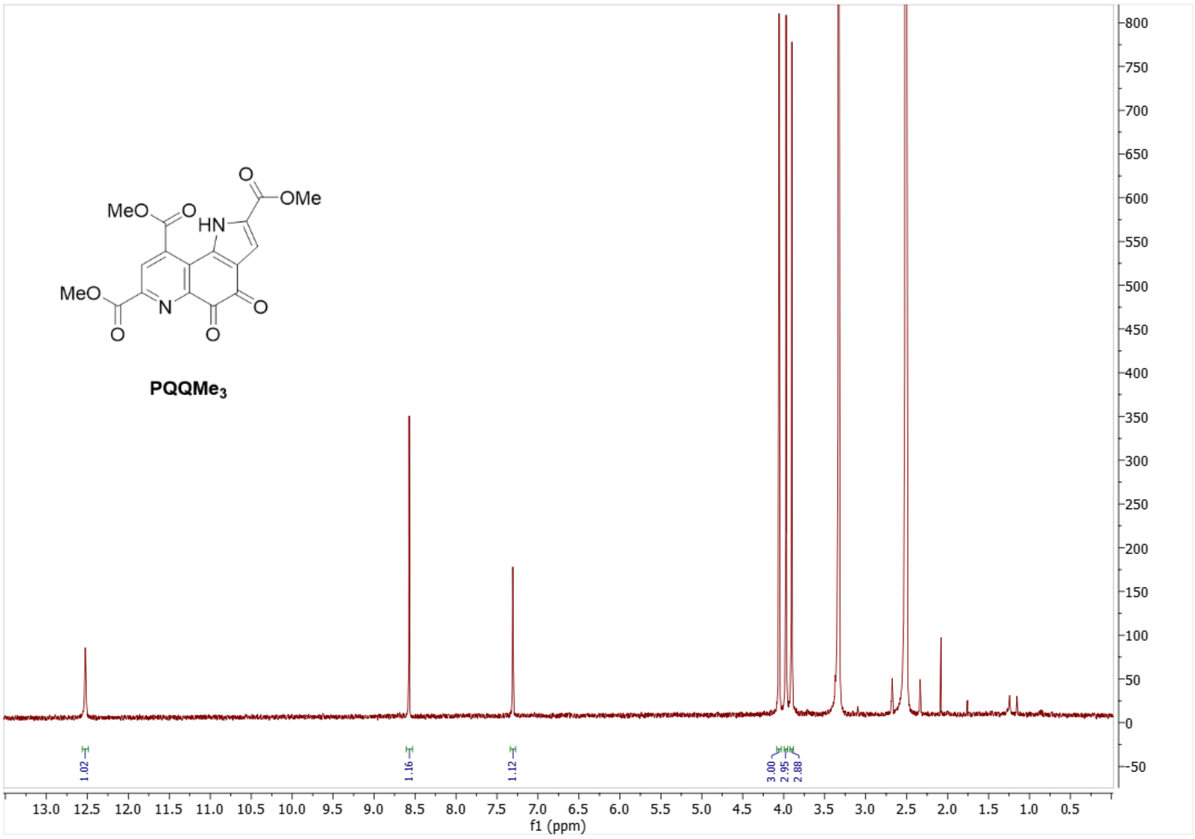

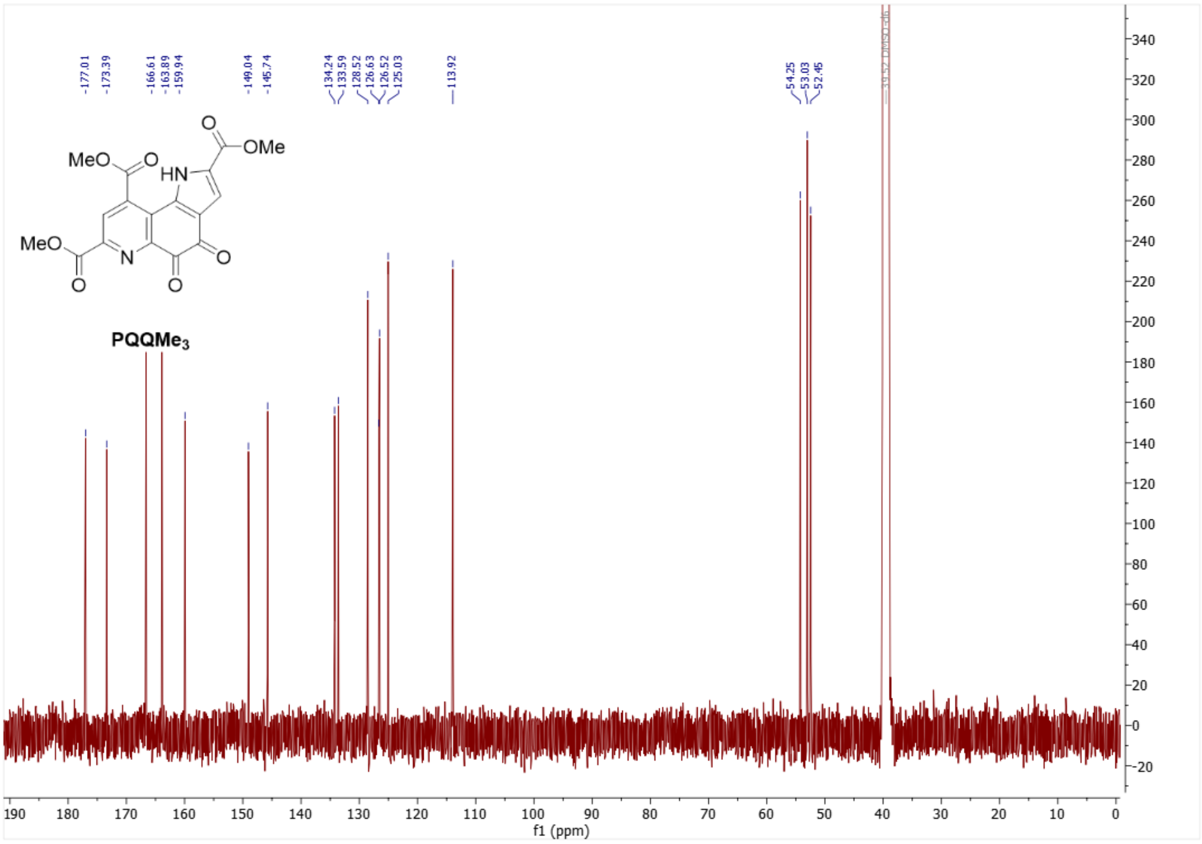

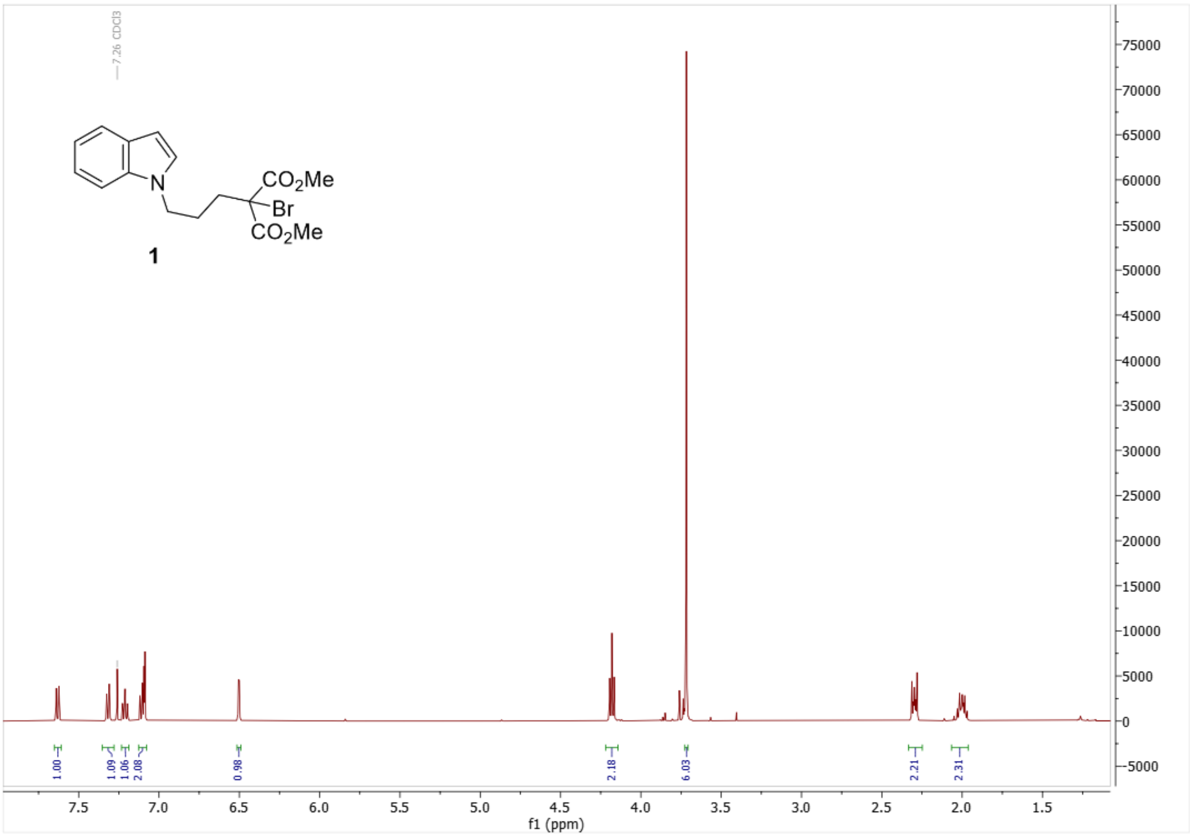
7.26 CDCl3
N
CO2Me
Br
CO2Me
1
1.00
1.09
1.06
2.08
0.98
2.18
6.03
2.21
2.31
75000
70000
65000
60000
55000
50000
45000
40000
35000
30000
25000
20000
15000
10000
5000
0
-5000
7.5
7.0
6.5
6.0
5.5
5.0
4.5
4.0
3.5
3.0
2.5
2.0
1.5
f1 (ppm)

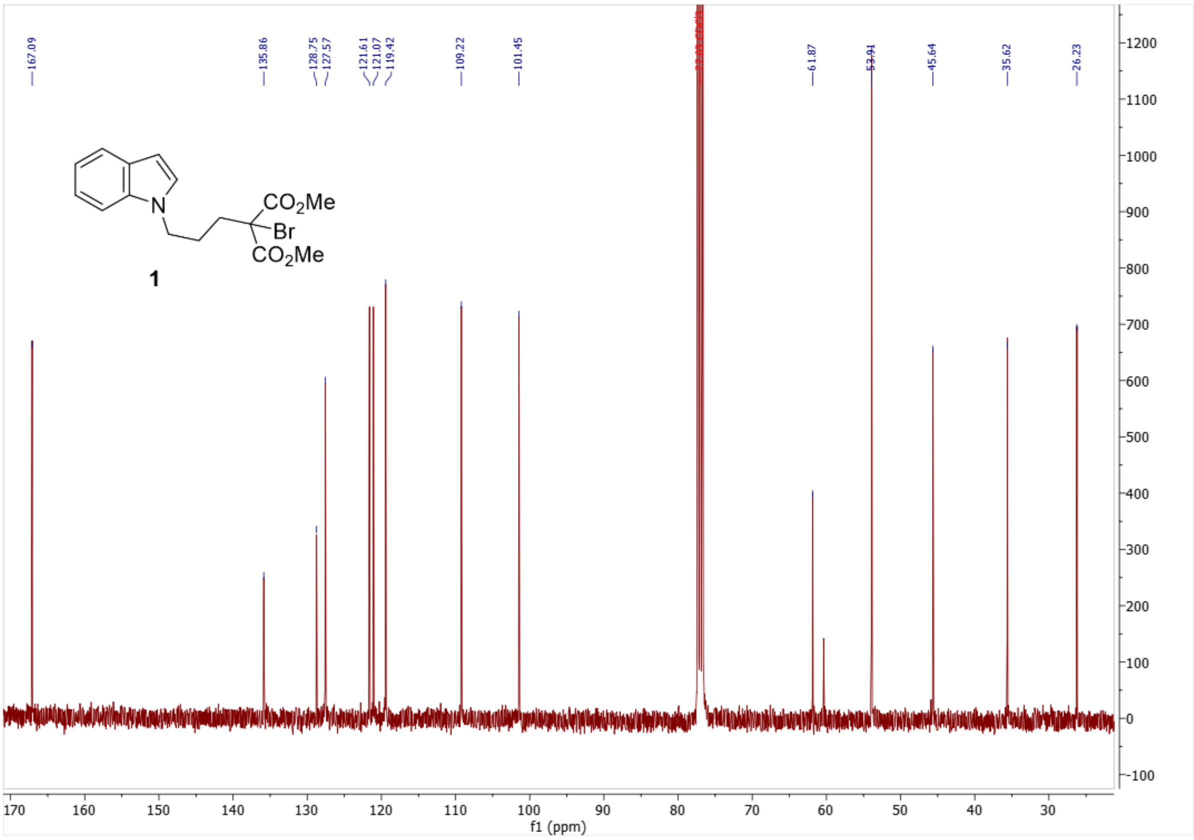
167.09
135.86
128.75
127.57
121.61
121.07
119.42
109.22
101.45
61.87
53.91
45.64
35.62
26.23
N
CO2Me
Br
CO2Me
1
1200
1100
1000
900
800
700
600
500
400
300
200
100
0
-100
170
160
150
140
130
120
110
100
90
80
70
60
50
40
30
f1 (ppm)

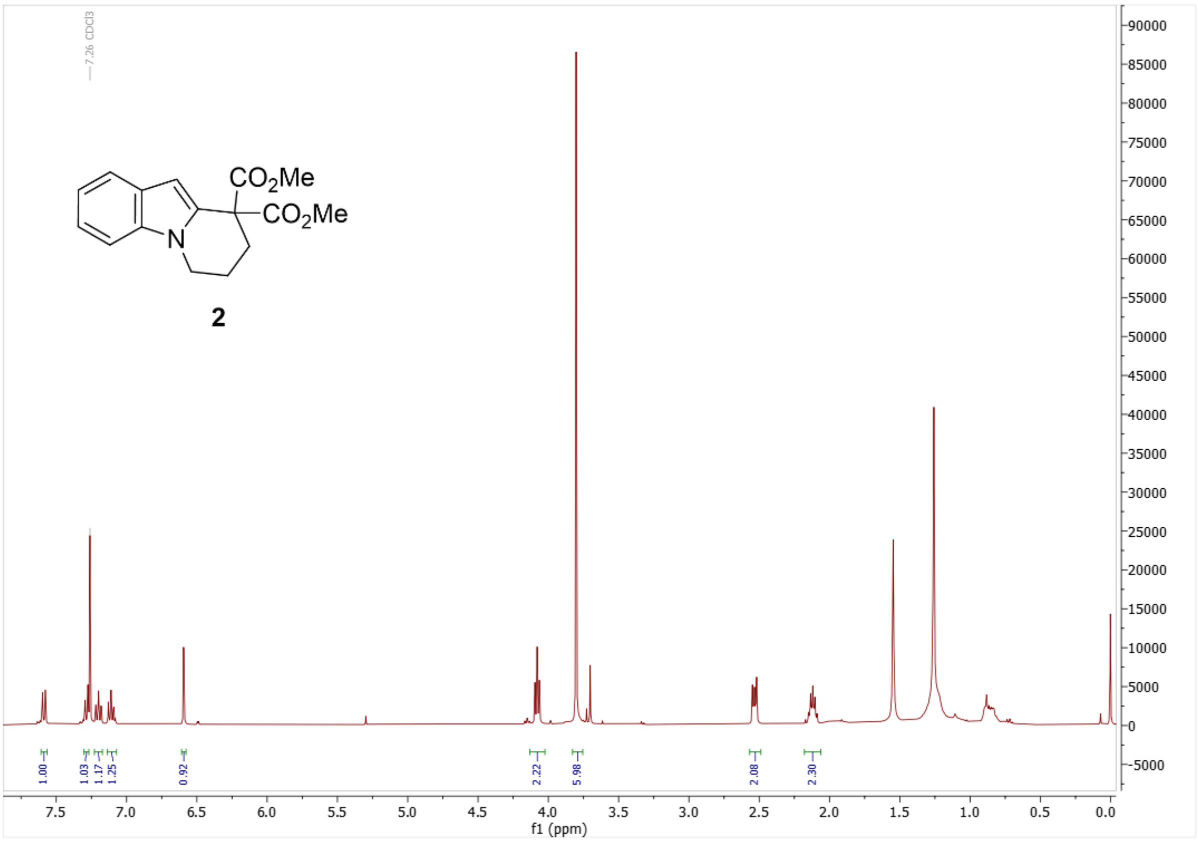
CO2Me
CO2Me
N
2
7.26 CDCl3
1.00
1.03
1.17
1.25
0.92
2.22
5.98
2.08
2.30
f1 (ppm)

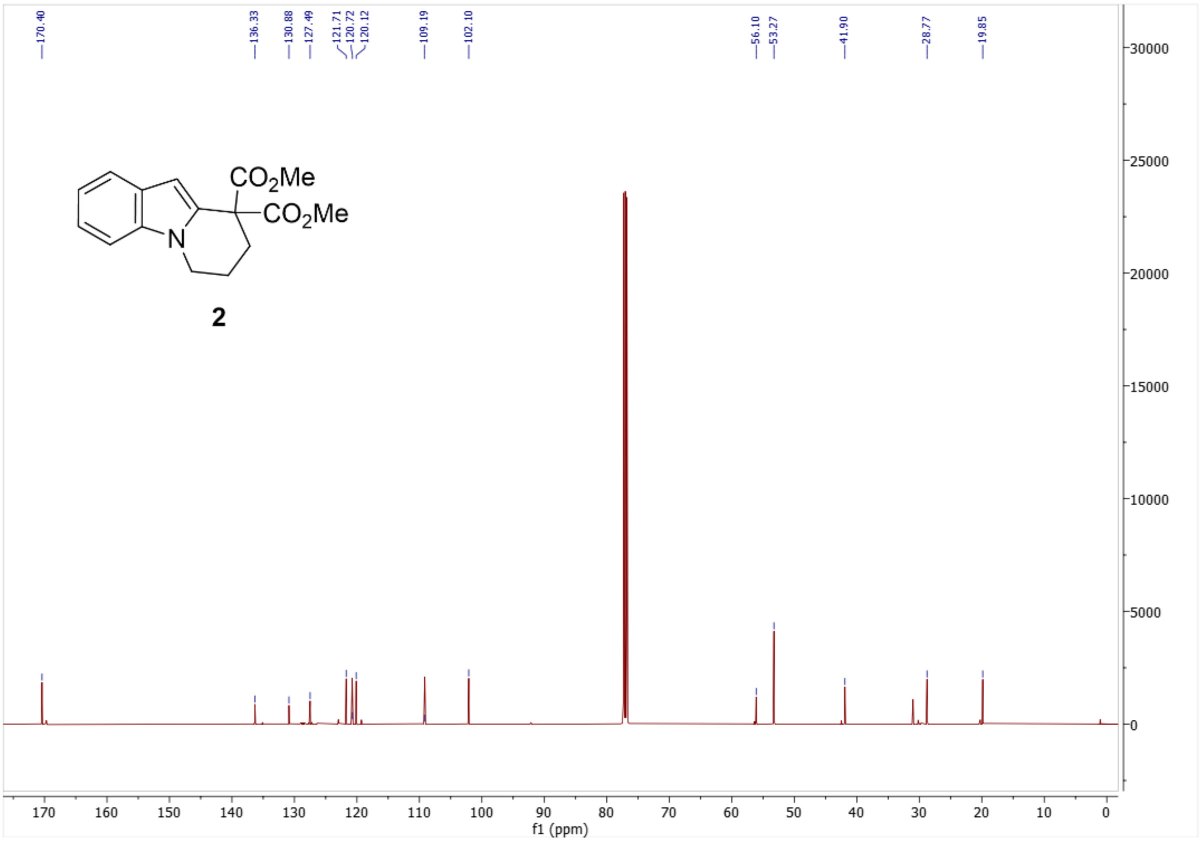
170.40
136.33
130.88
127.49
121.71
120.72
120.12
109.19
102.10
56.10
53.27
41.90
28.77
19.85
CO2Me
CO2Me
N
2
f1 (ppm)

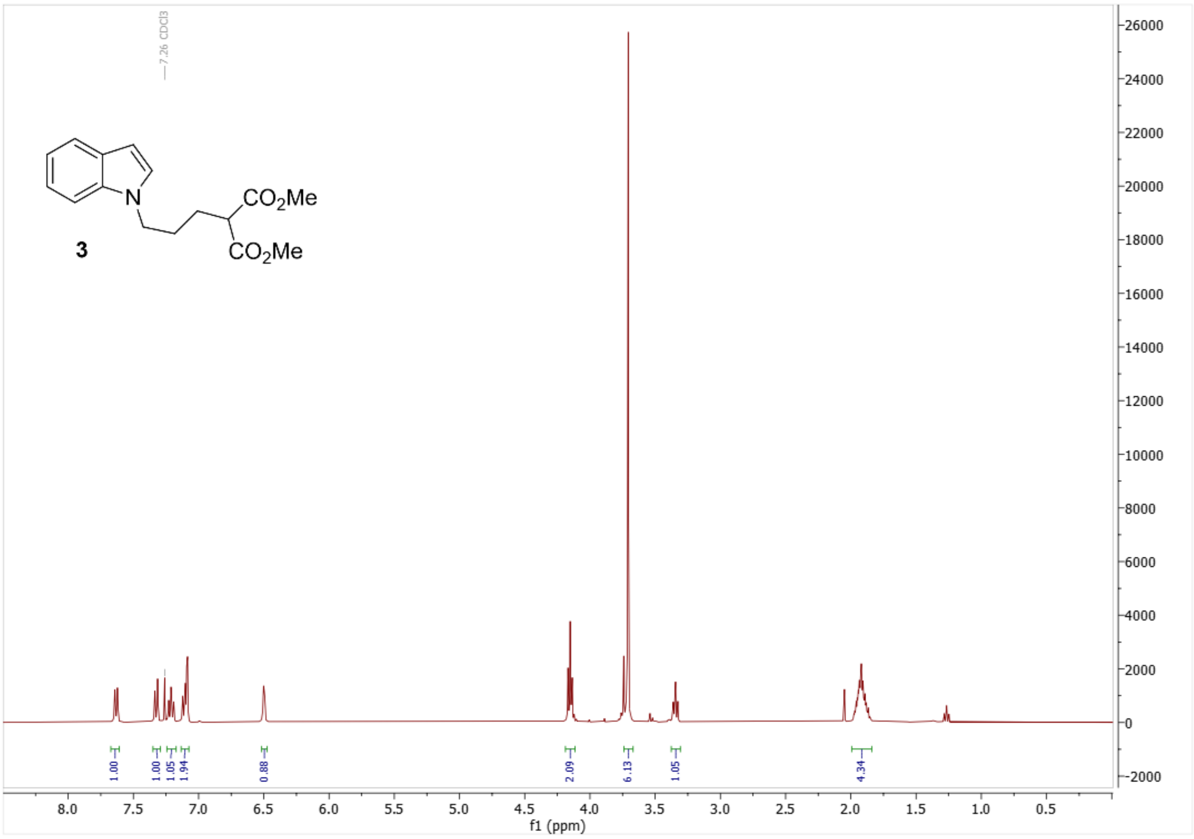

7.26 CDCl3
CO2Me
CO2Me
3
N
f1 (ppm)

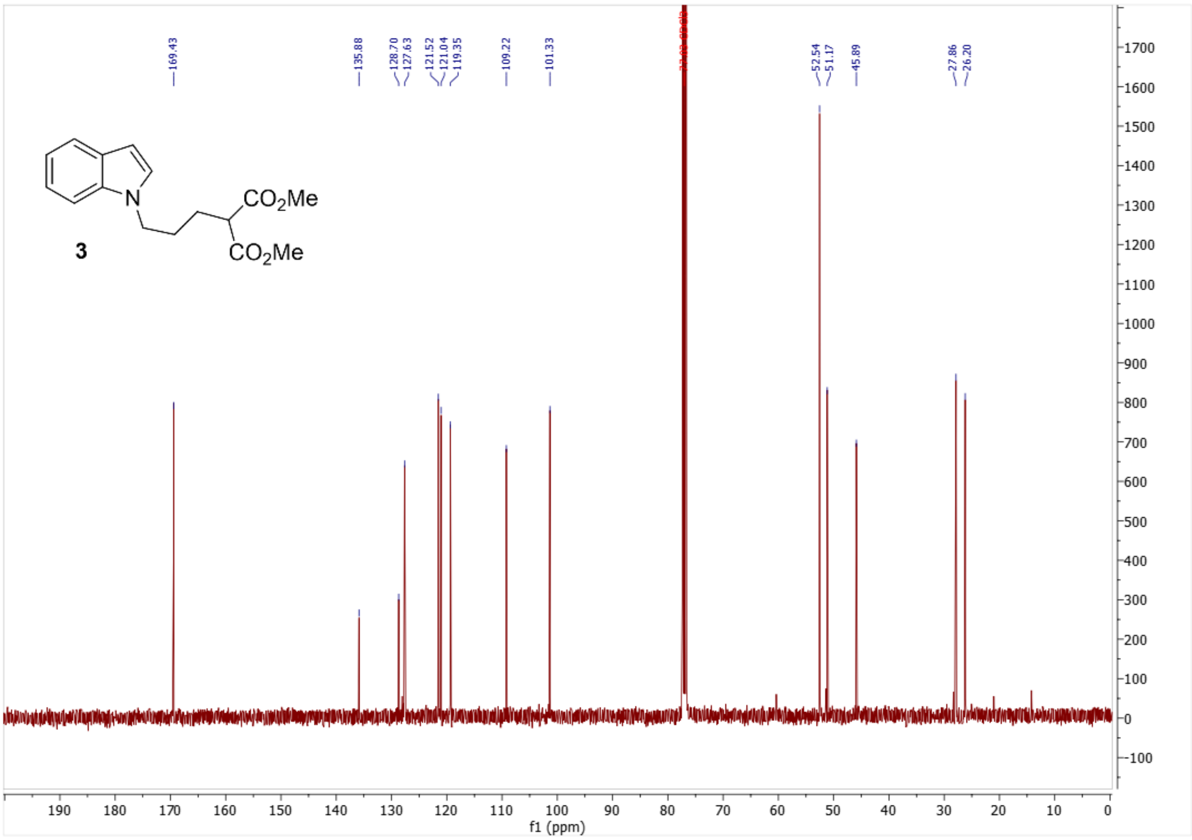

169.43
135.88
128.70
127.63
121.52
121.04
119.35
109.22
101.33
52.54
51.17
45.89
27.86
26.20
N
CO2Me
CO2Me
3
f1 (ppm)

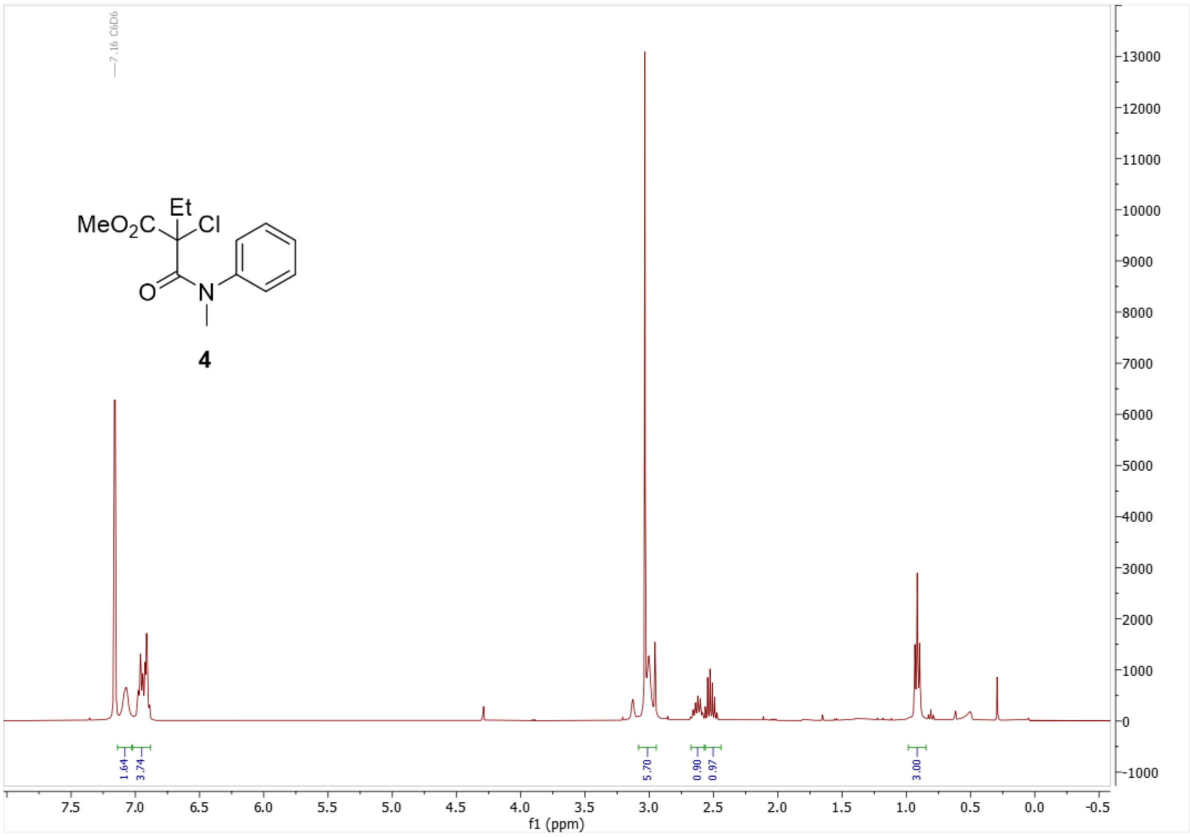
7.16 C6D6
MeO2C
Et
Cl
O
N
4
1.64
3.74
5.70
0.90
0.97
3.00
f1 (ppm)

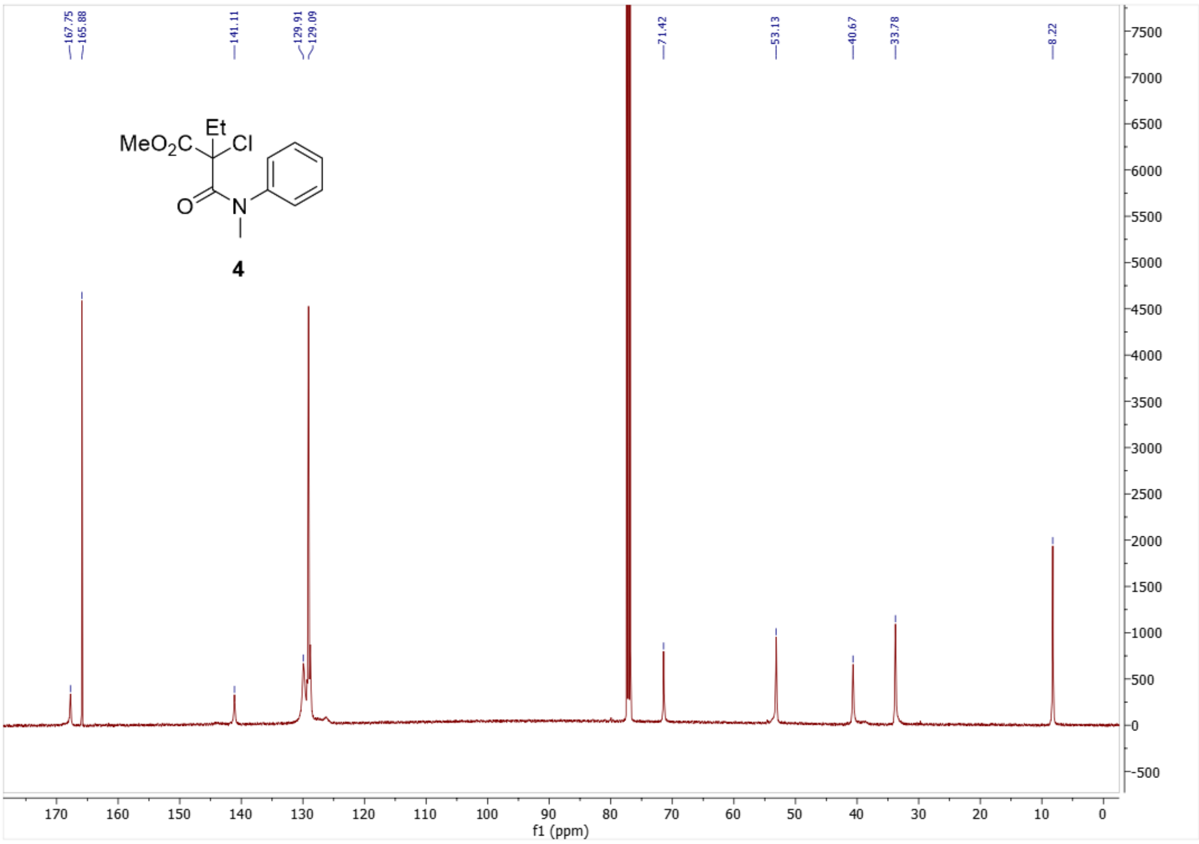
167.75
165.88
141.11
129.91
129.09
71.42
53.13
40.67
33.78
8.22
MeO2C
Et
Cl
O
N
4
f1 (ppm)

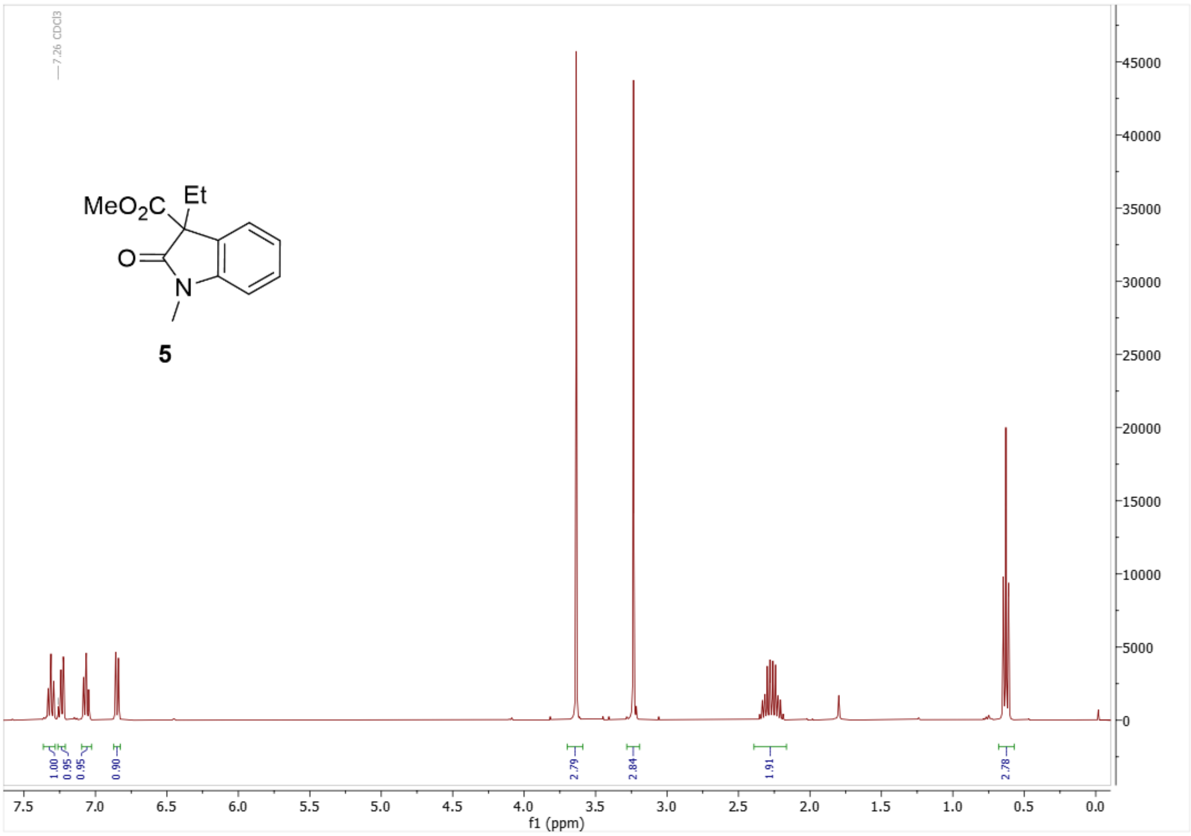
7.26 CDCl3
MeO2C
Et
O
N
5
1.00
0.95
0.95
0.90
2.79
2.84
1.91
2.78
f1 (ppm)

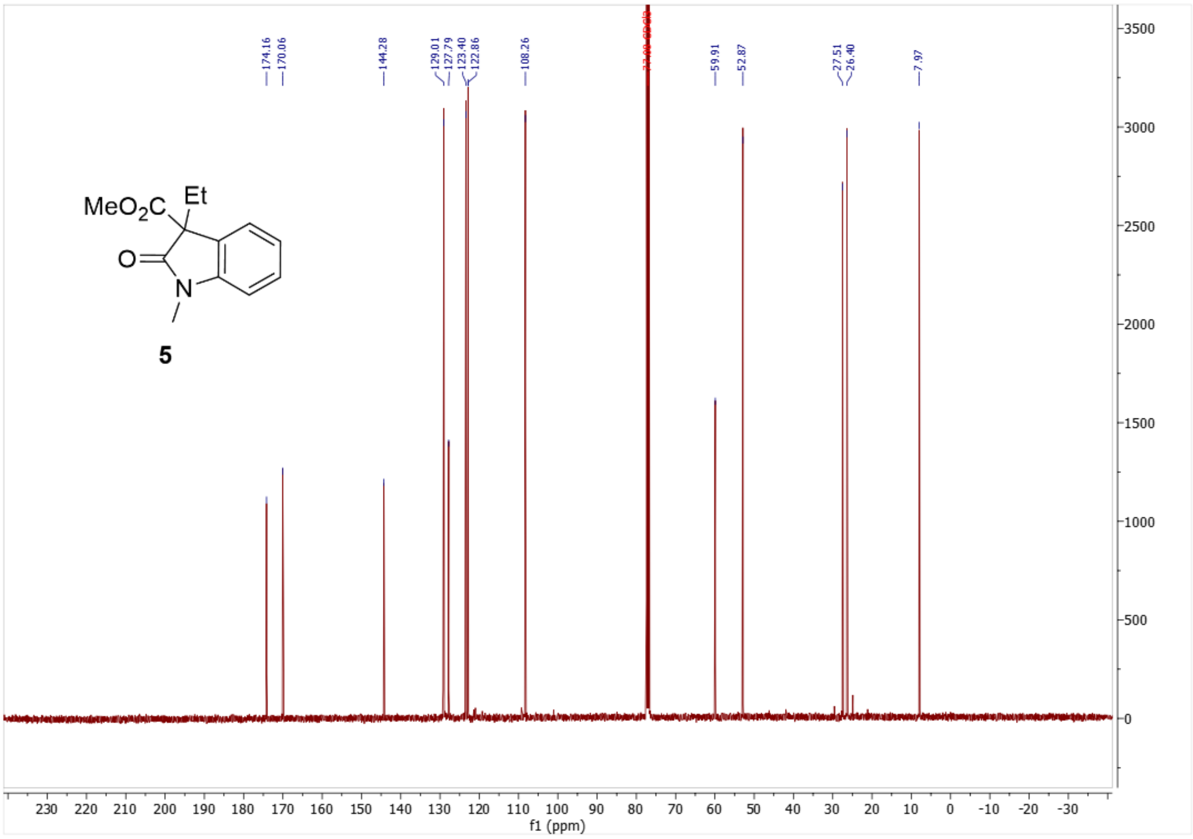
174.16
170.06
144.28
129.01
127.79
123.40
122.86
108.26
59.91
52.87
27.51
26.40
7.97
MeO2C
Et
O
N
5
f1 (ppm)

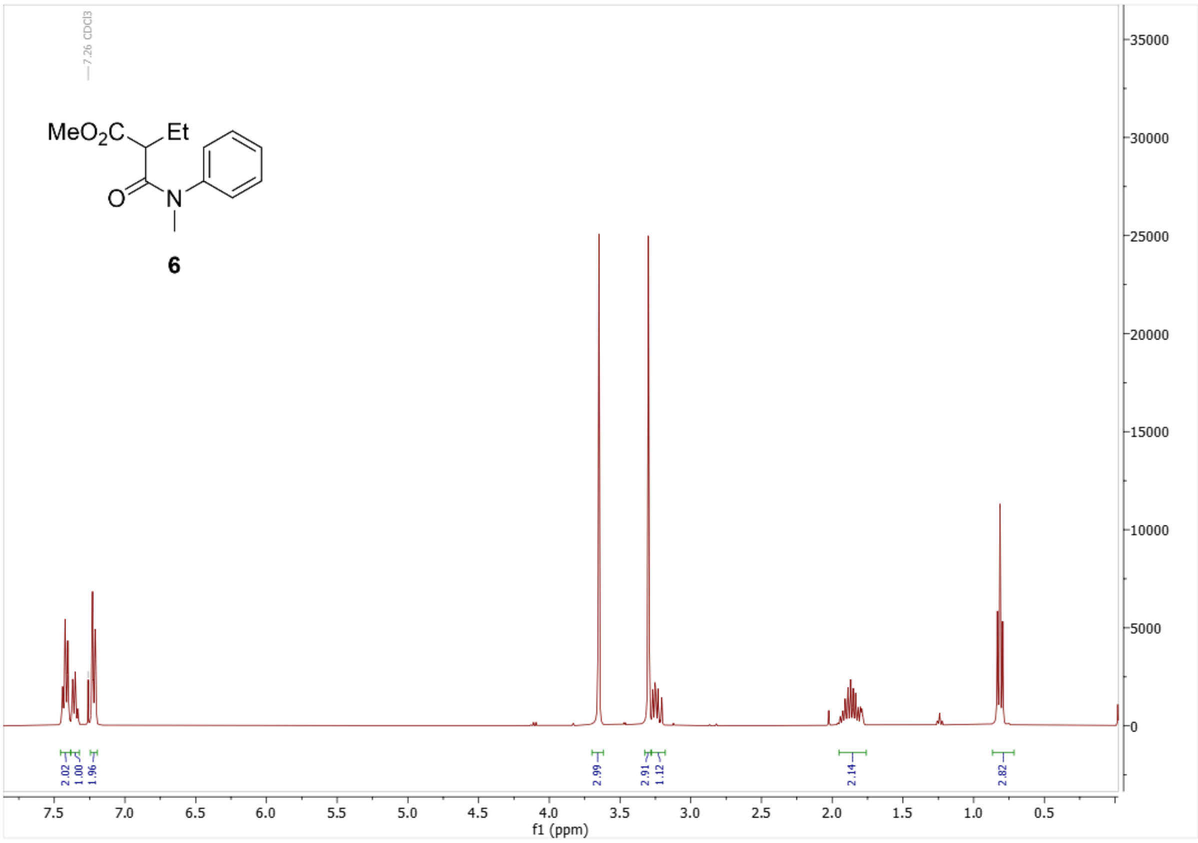
7.26 CDCl3
MeO2C
Et
O
N
6
2.02
1.00
1.96
2.99
2.91
1.12
2.14
2.82
f1 (ppm)

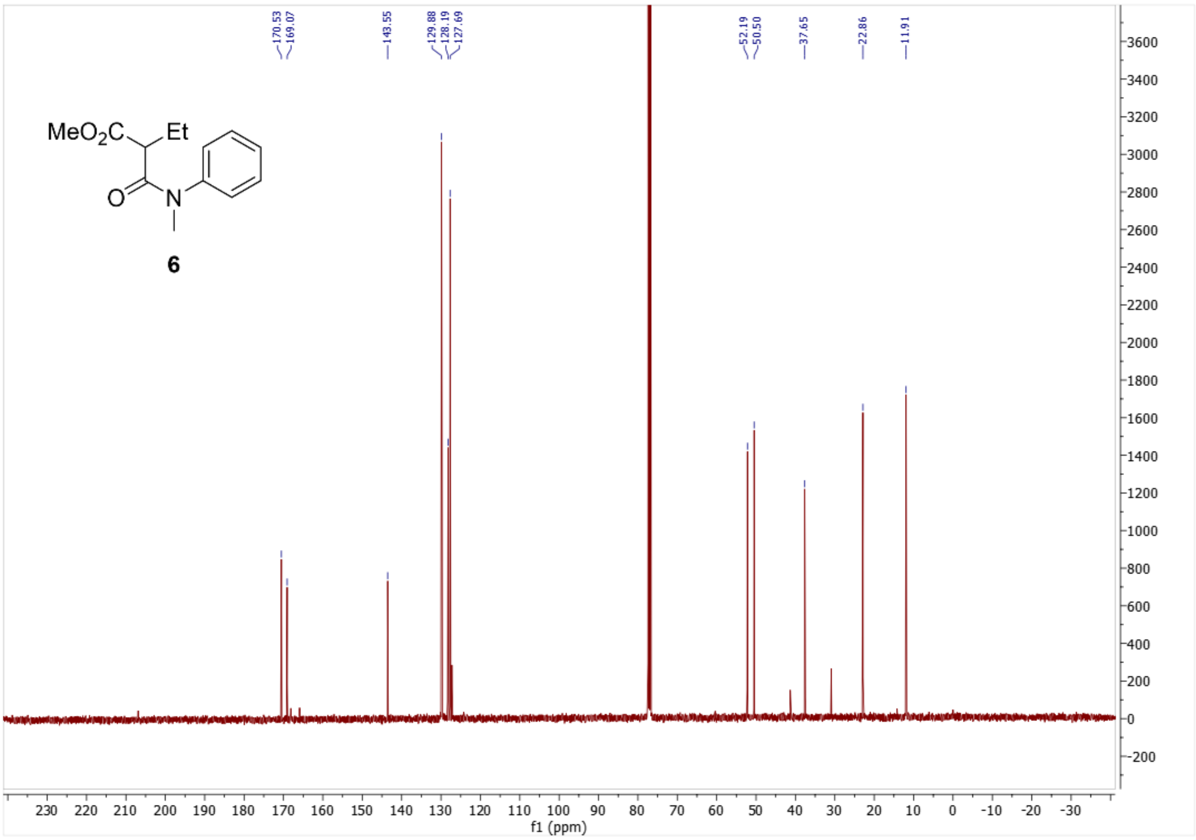
170.53
169.07
143.55
129.88
128.19
127.69
52.19
50.50
37.65
22.86
11.91
MeO2C
Et
O
N
6
f1 (ppm)

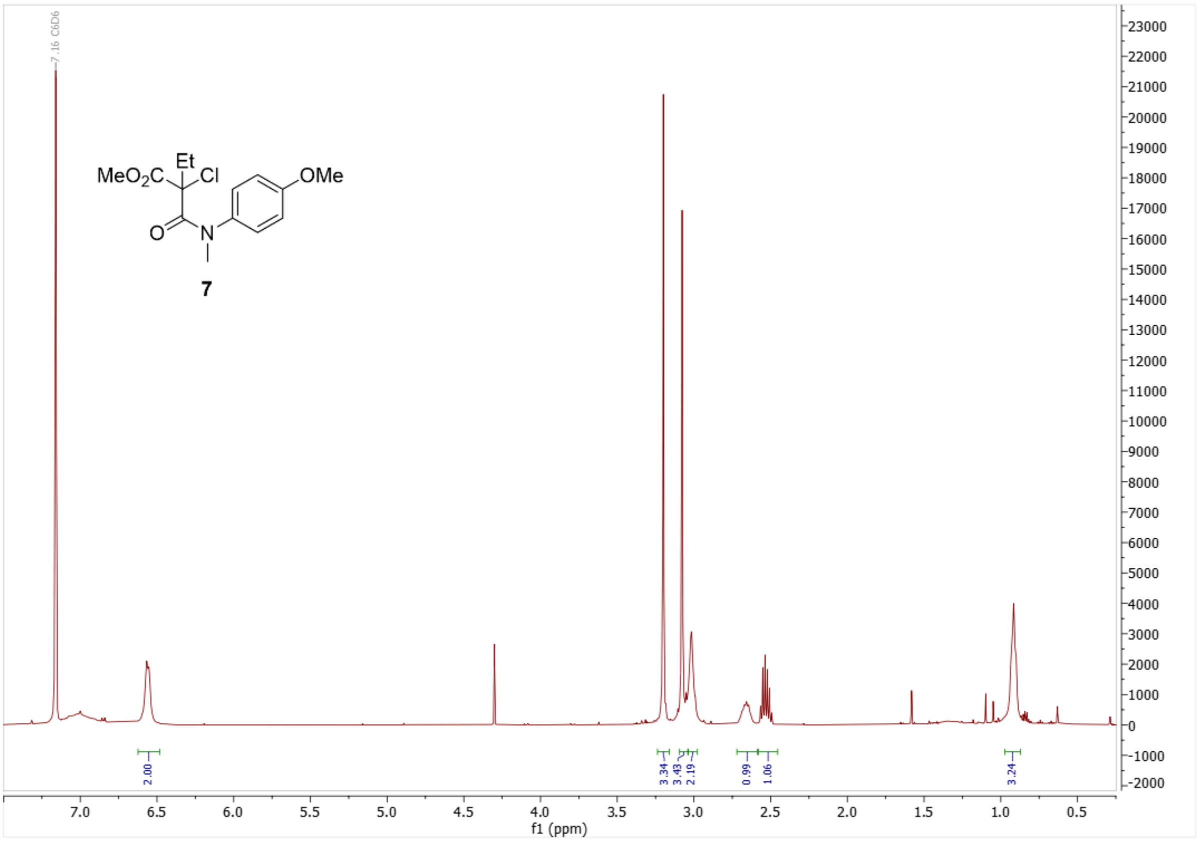
7.16 C6D6
MeO2C
Et
Cl
OMe
O
N
7
2.00
3.34
3.43
2.19
0.99
1.06
3.24
f1 (ppm)

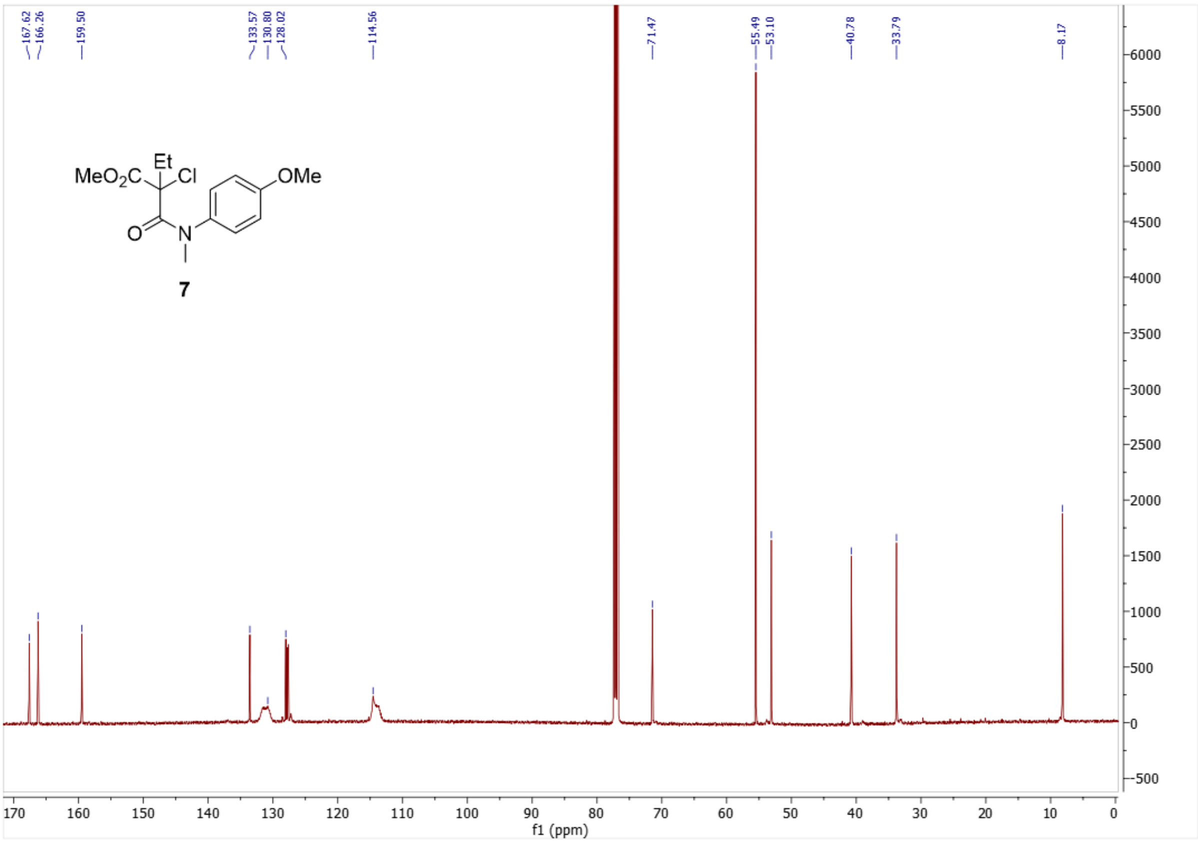
167.62
166.26
159.50
133.57
130.80
128.02
114.56
71.47
55.49
53.10
40.78
33.79
8.17
MeO2C
Et
Cl
OMe
O
N
7
f1 (ppm)

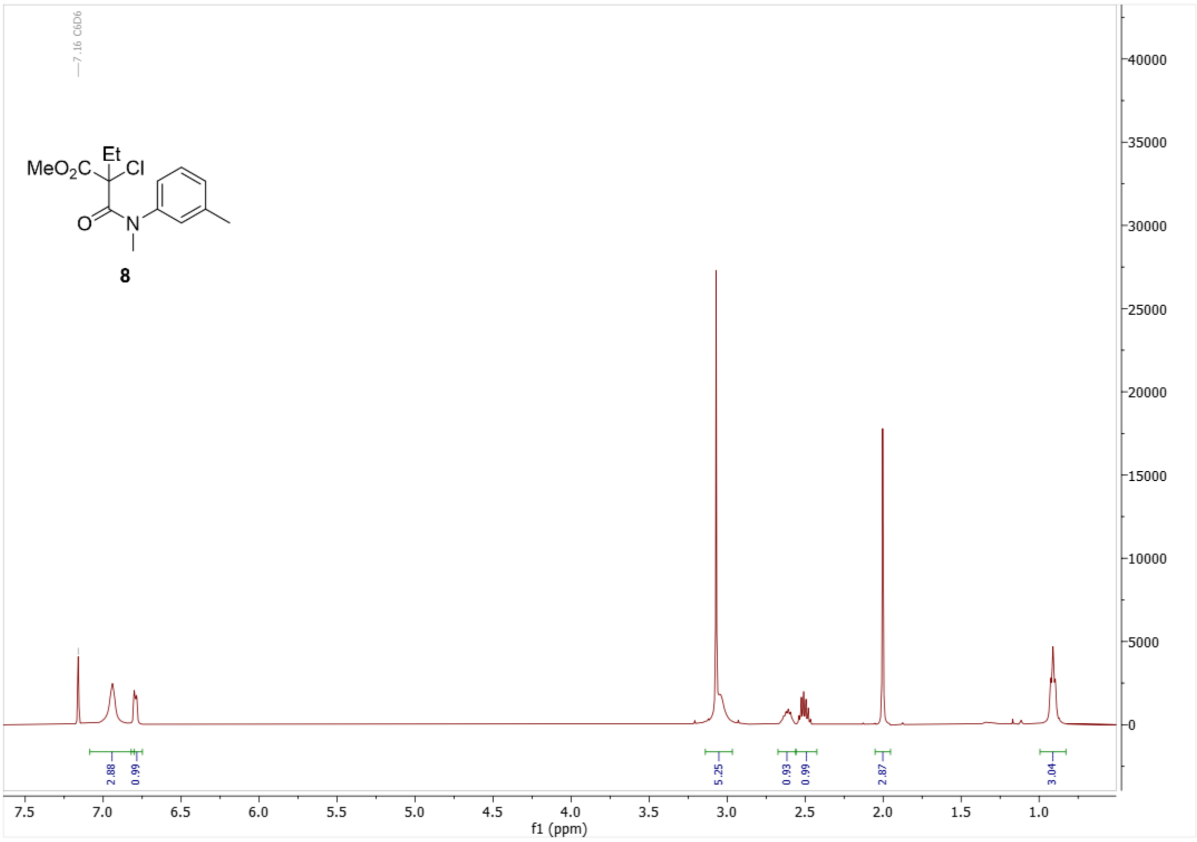

7.16 C6D6
MeO2C
Et
Cl
O
N
8
2.88
0.99
5.25
0.93
0.99
2.87
3.04
f1 (ppm)

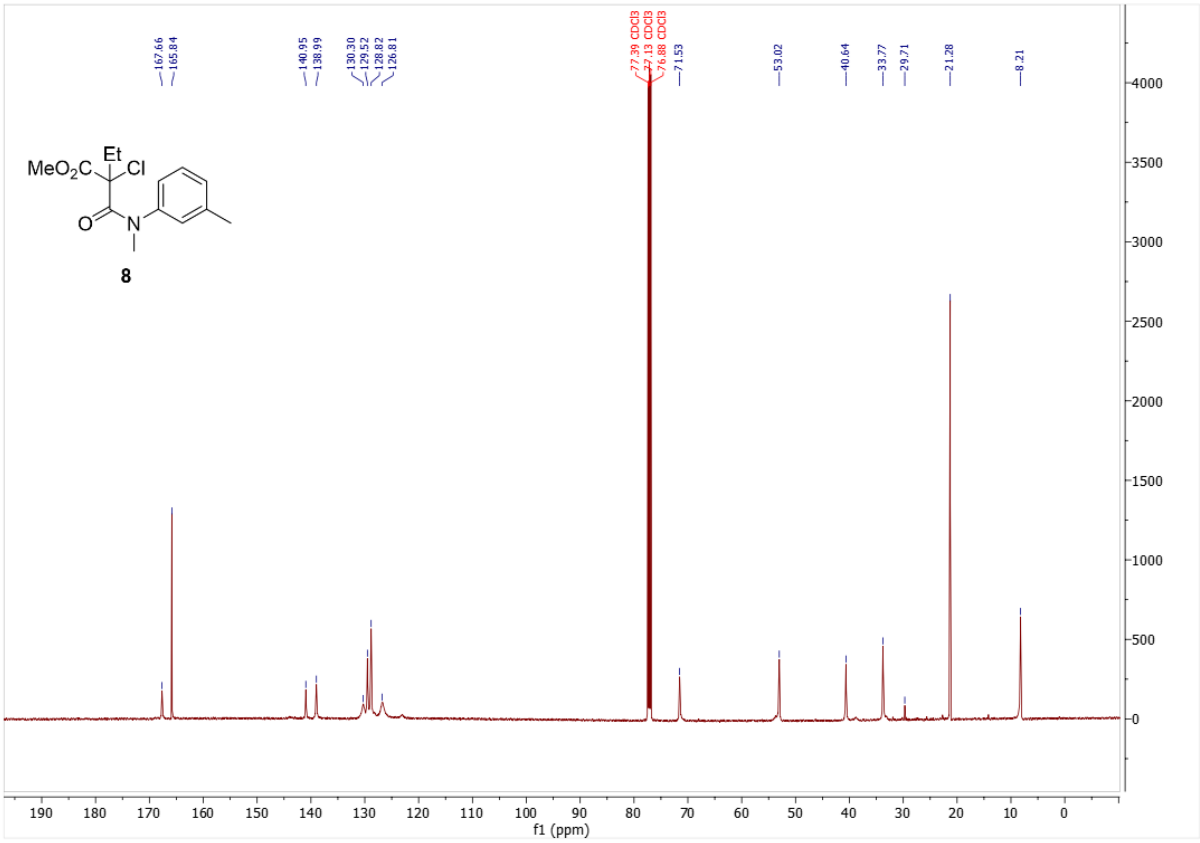

167.66
165.84
140.95
138.99
130.30
129.52
128.82
126.81
77.39 CDCl3
77.13 CDCl3
76.88 CDCl3
71.53
53.02
40.64
33.77
29.71
21.28
8.21
MeO2C
Et
Cl
O
N
8
f1 (ppm)

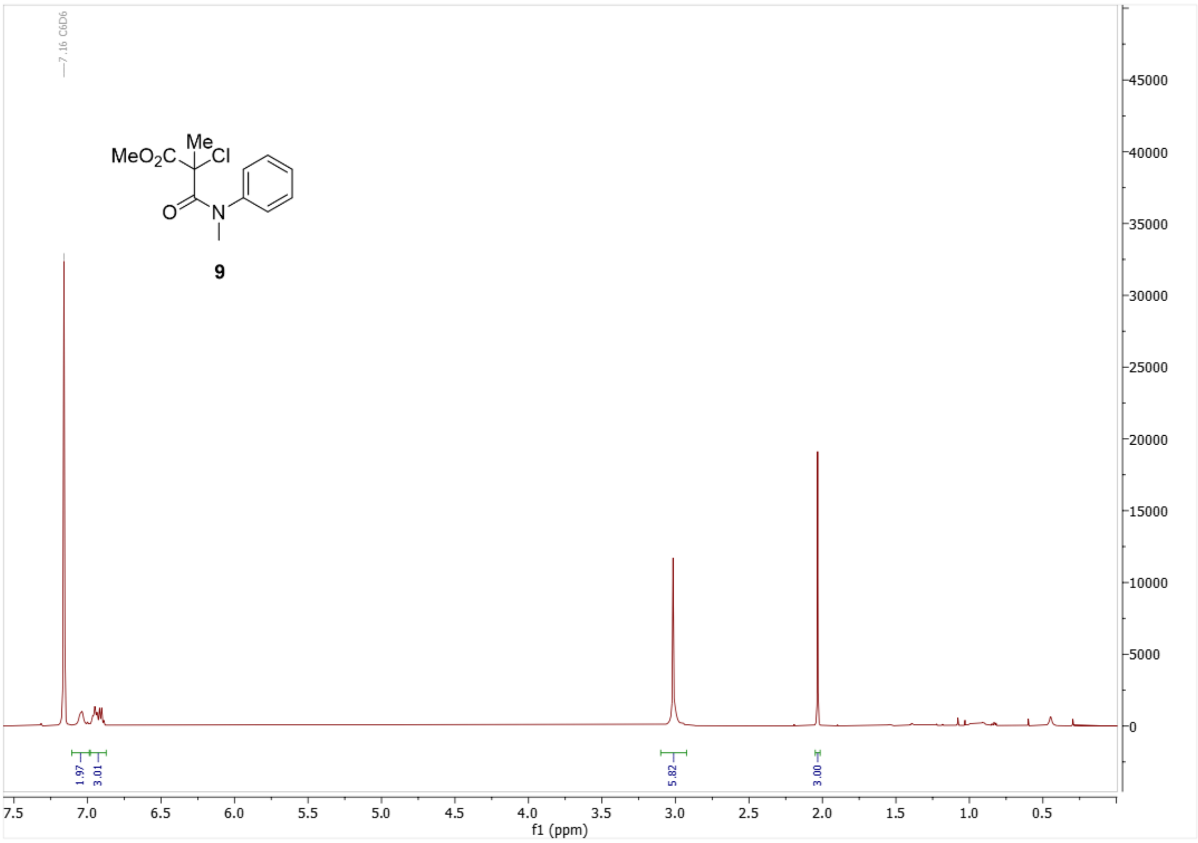
7.16 C6D6
MeO2C
Me
Cl
O
N
9
1.97
3.01
5.82
3.00
f1 (ppm)

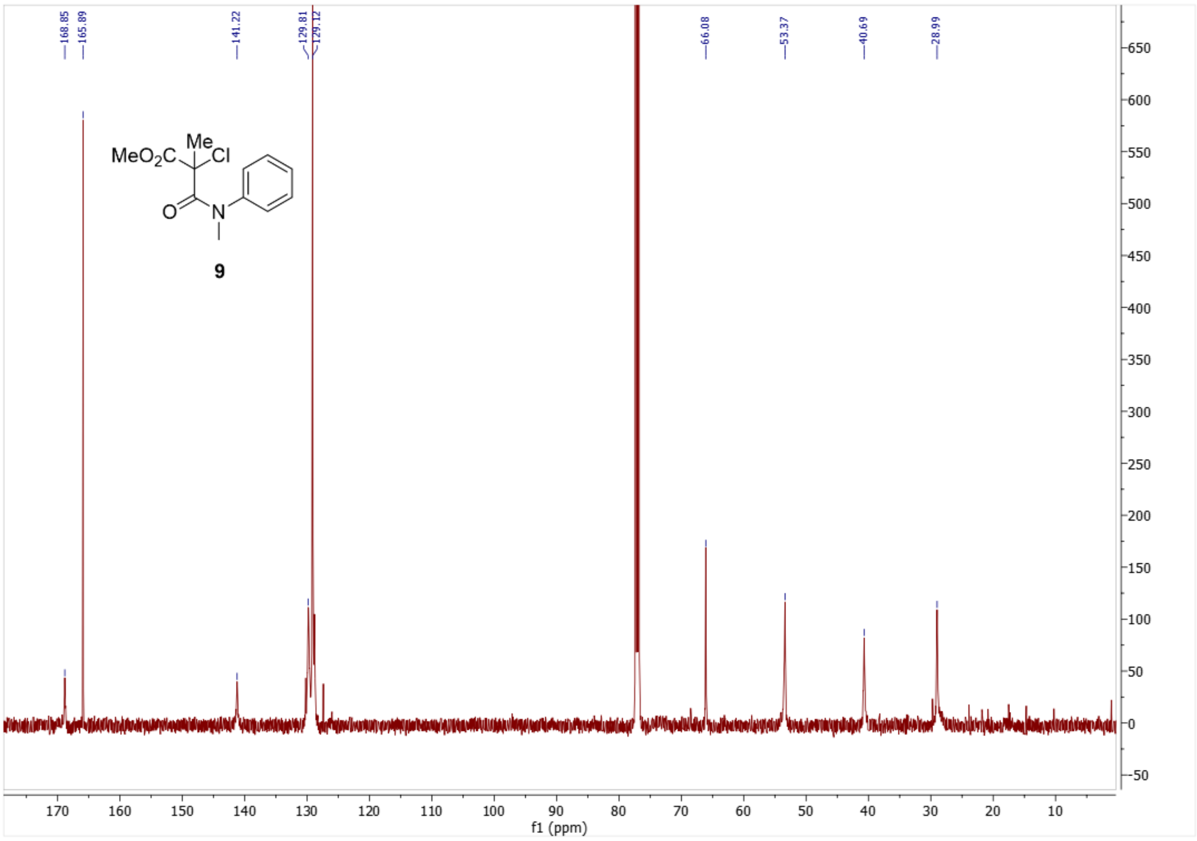
168.85
165.89
141.22
129.81
129.12
66.08
53.37
40.69
28.99
MeO2C
Me
Cl
O
N
9
f1 (ppm)

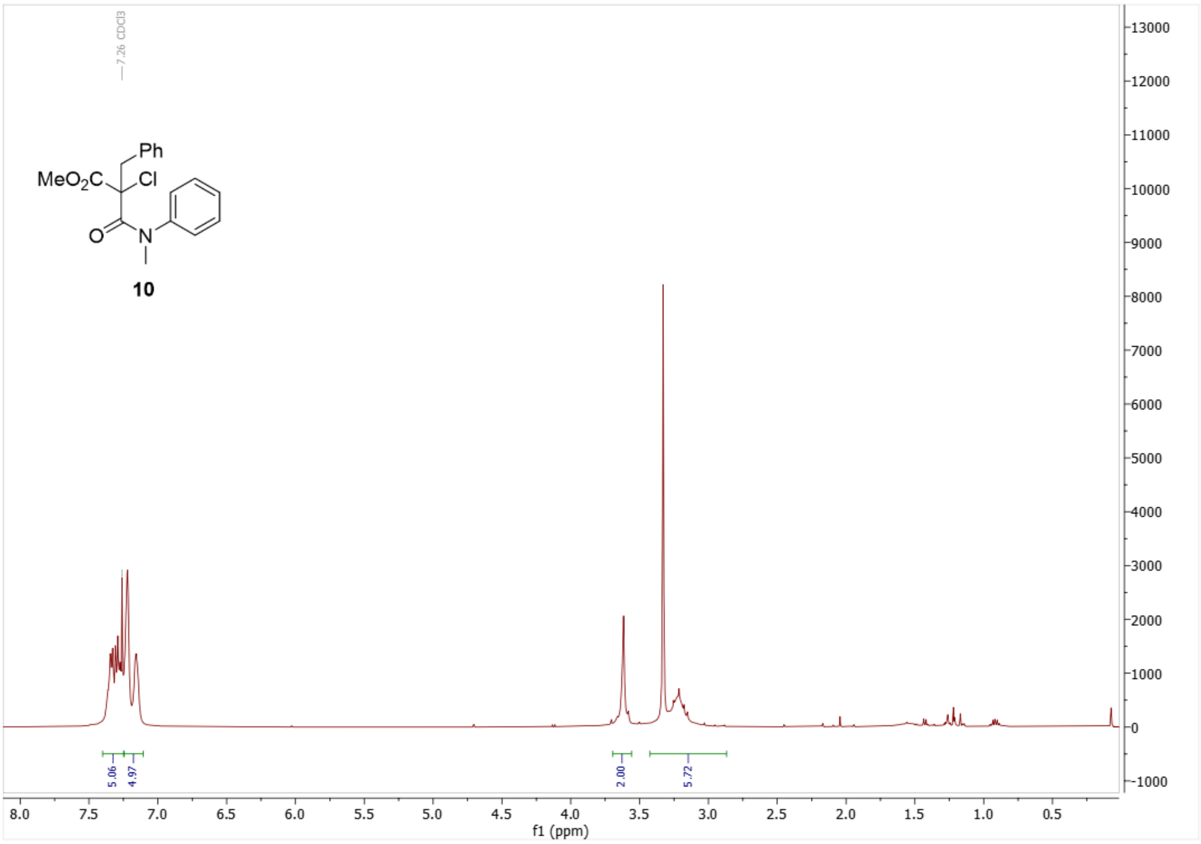
7.26 CDCl3
Ph
MeO2C
Cl
O
N
10
5.06
4.97
2.00
5.72
f1 (ppm)

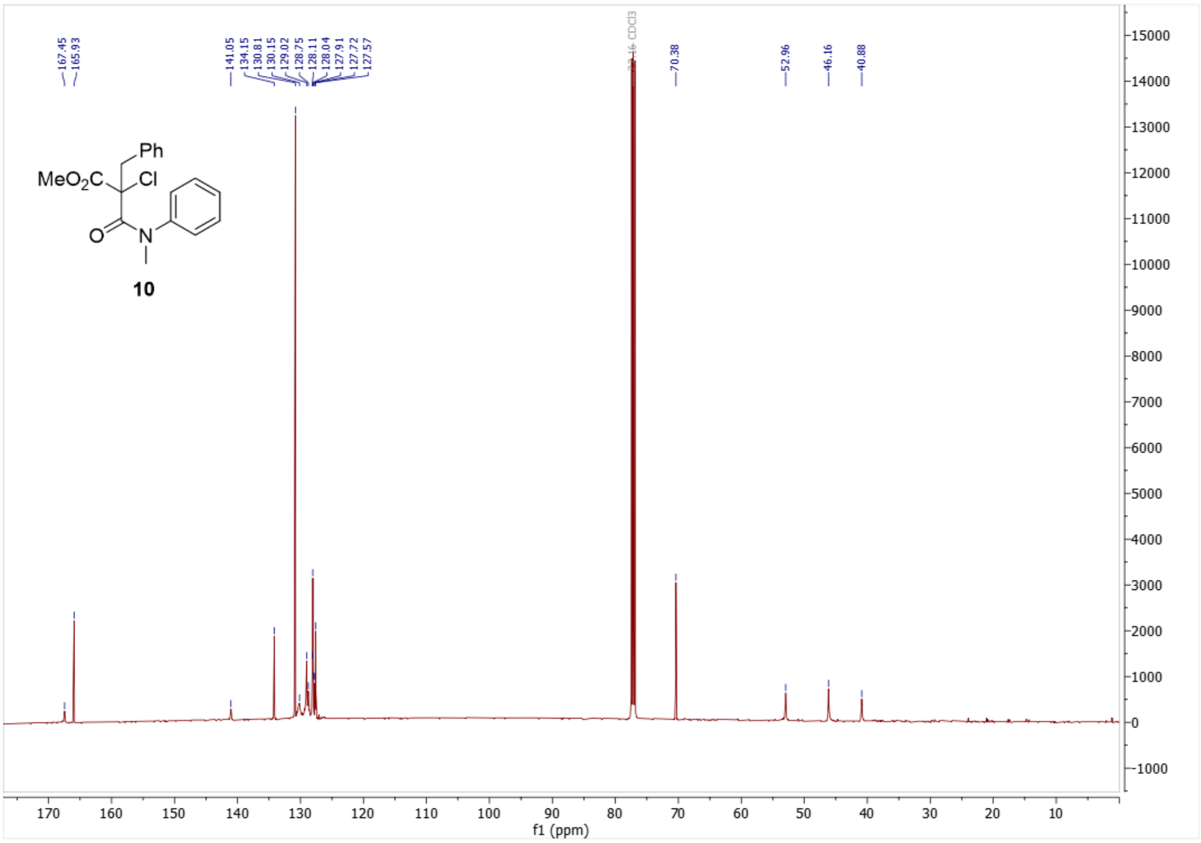
167.45
165.93
141.05
134.15
130.81
130.15
129.02
128.75
128.11
128.04
127.91
127.72
127.57
70.38
52.96
46.16
40.88
Ph
MeO2C
Cl
O
N
10
f1 (ppm)

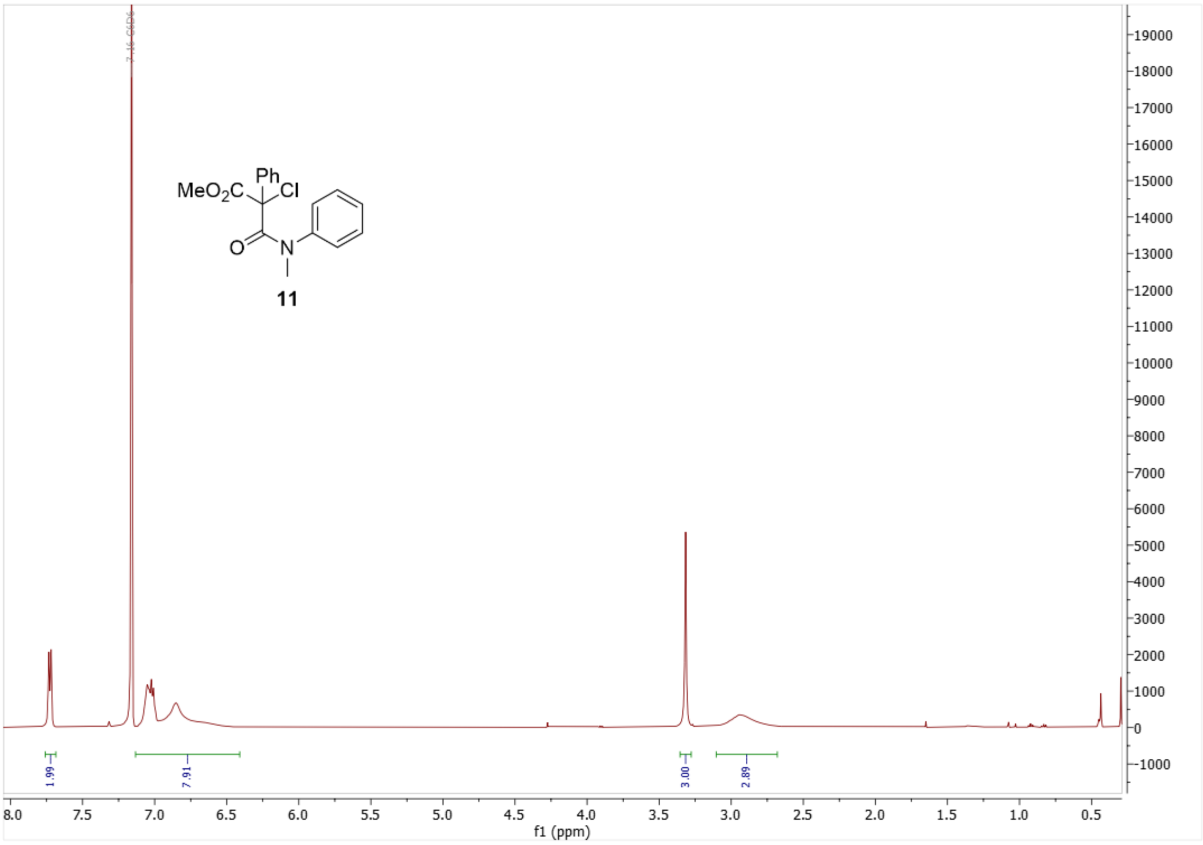
MeO2C
Ph
Cl
O
N
11
1.99
7.91
3.00
2.89
f1 (ppm)

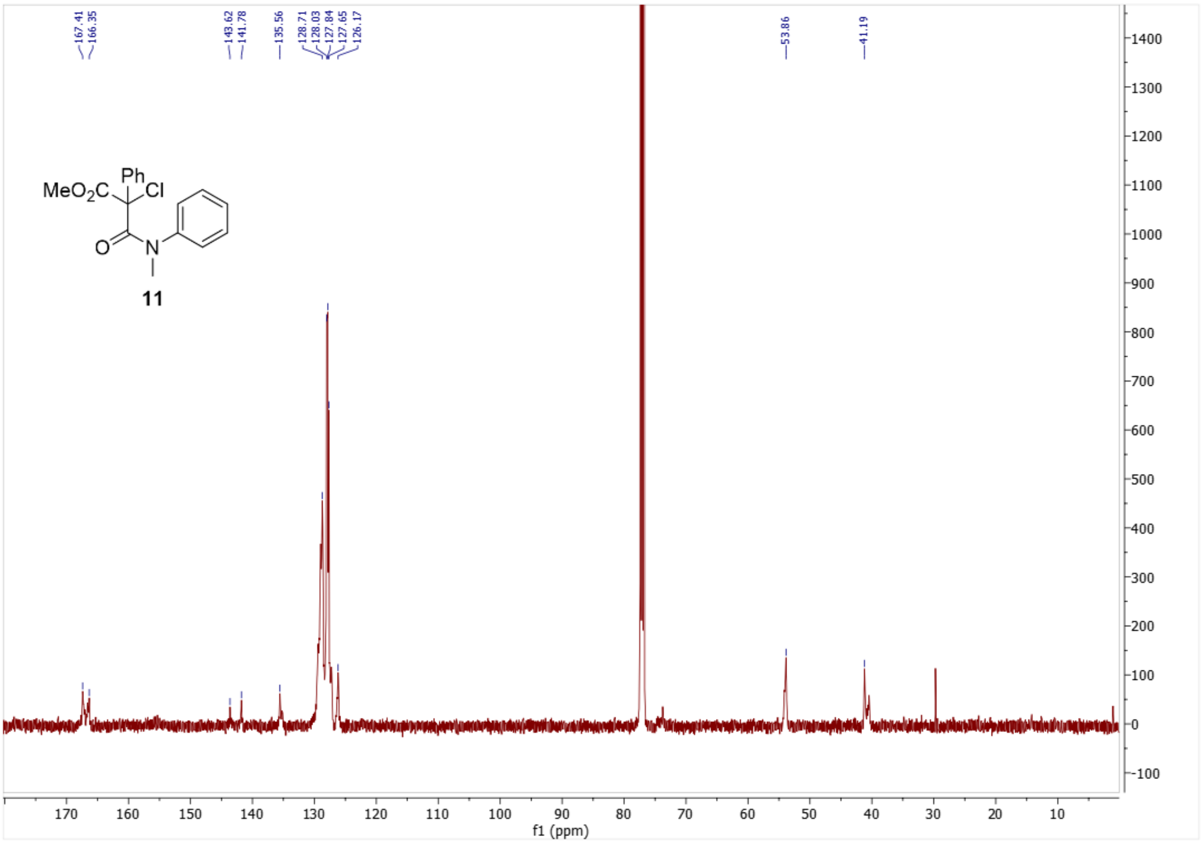
167.41
166.35
143.62
141.78
135.56
128.71
128.03
127.84
127.65
126.17
53.86
41.19
MeO2C
Ph
Cl
O
N
11
f1 (ppm)

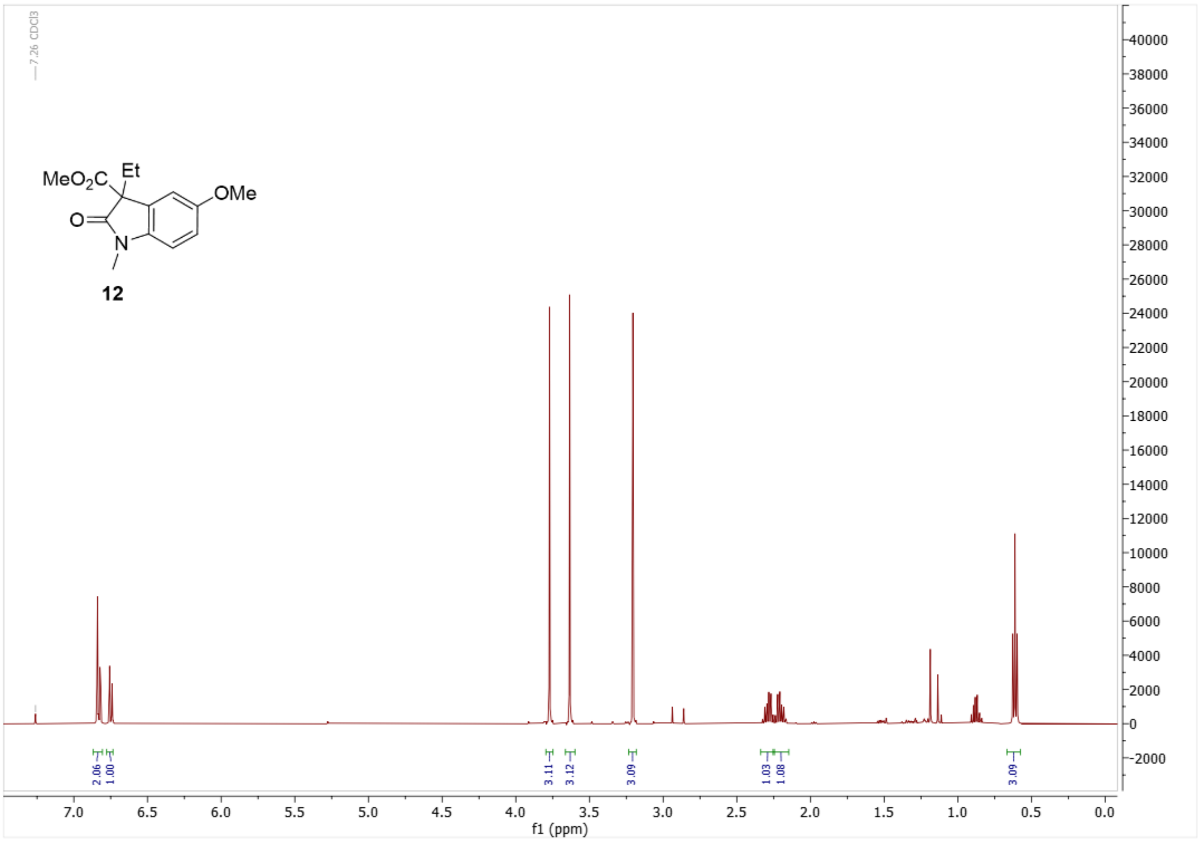

7.26 CDCl3
MeO2C
Et
OMe
O
N
12
2.06
1.00
3.11
3.12
3.09
1.03
1.08
3.09
f1 (ppm)

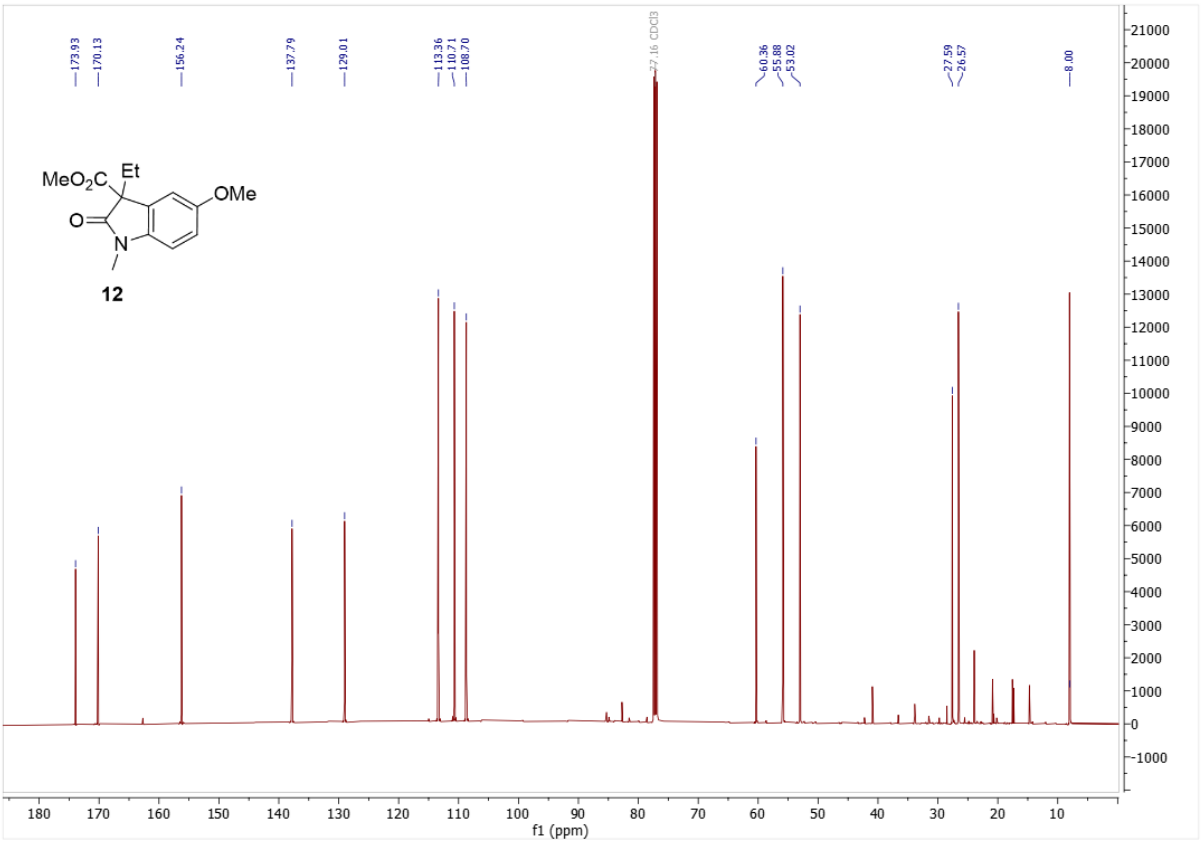

173.93
170.13
156.24
137.79
129.01
113.36
110.71
108.70
77.16 CDCl3
60.36
55.88
53.02
27.59
26.57
8.00
MeO2C
Et
OMe
O
N
12
f1 (ppm)

Compound **13** was obtained by chemical synthesis as a mixture of regioisomers methyl 3-ethyl-1,4-dimethyl-2-oxoindoline-3-carboxylate and methyl 3-ethyl-1,6-dimethyl-2-oxoindoline-3-carboxylate in ratio 2.7:1 respectively.

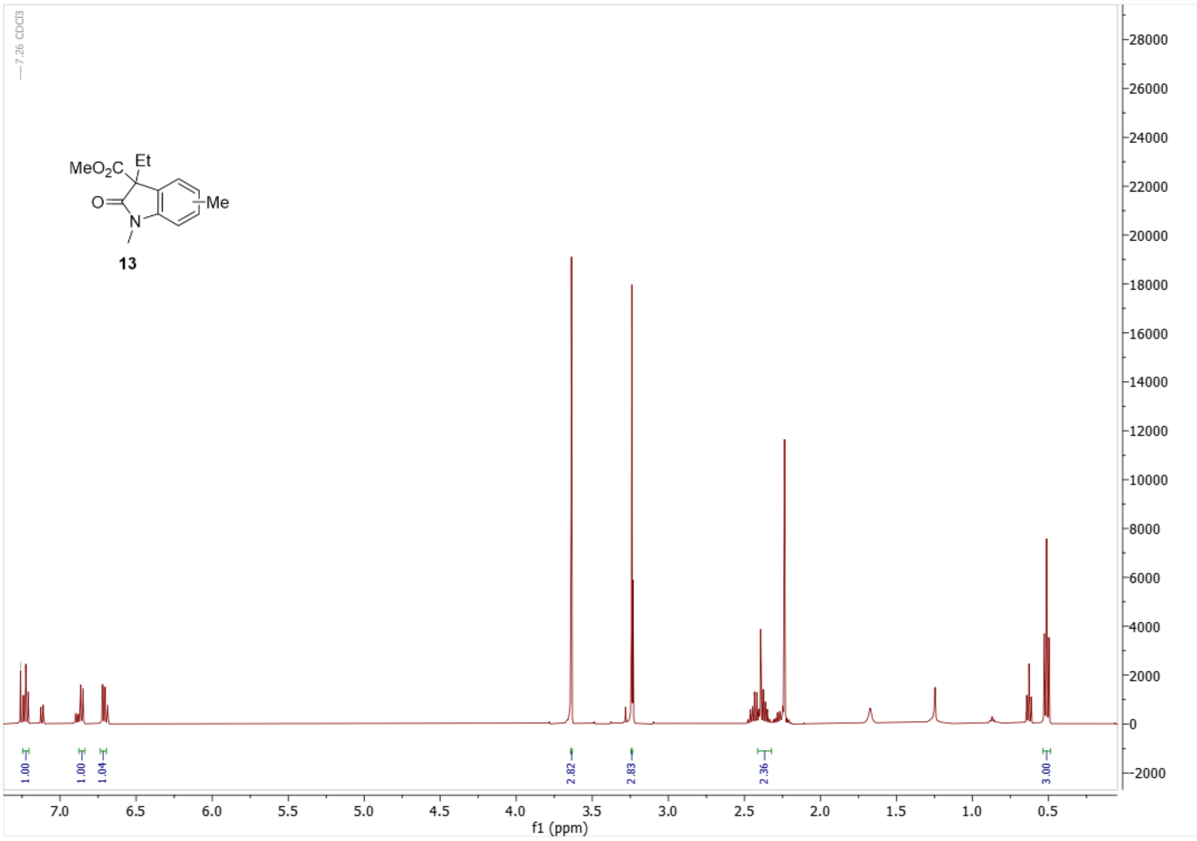

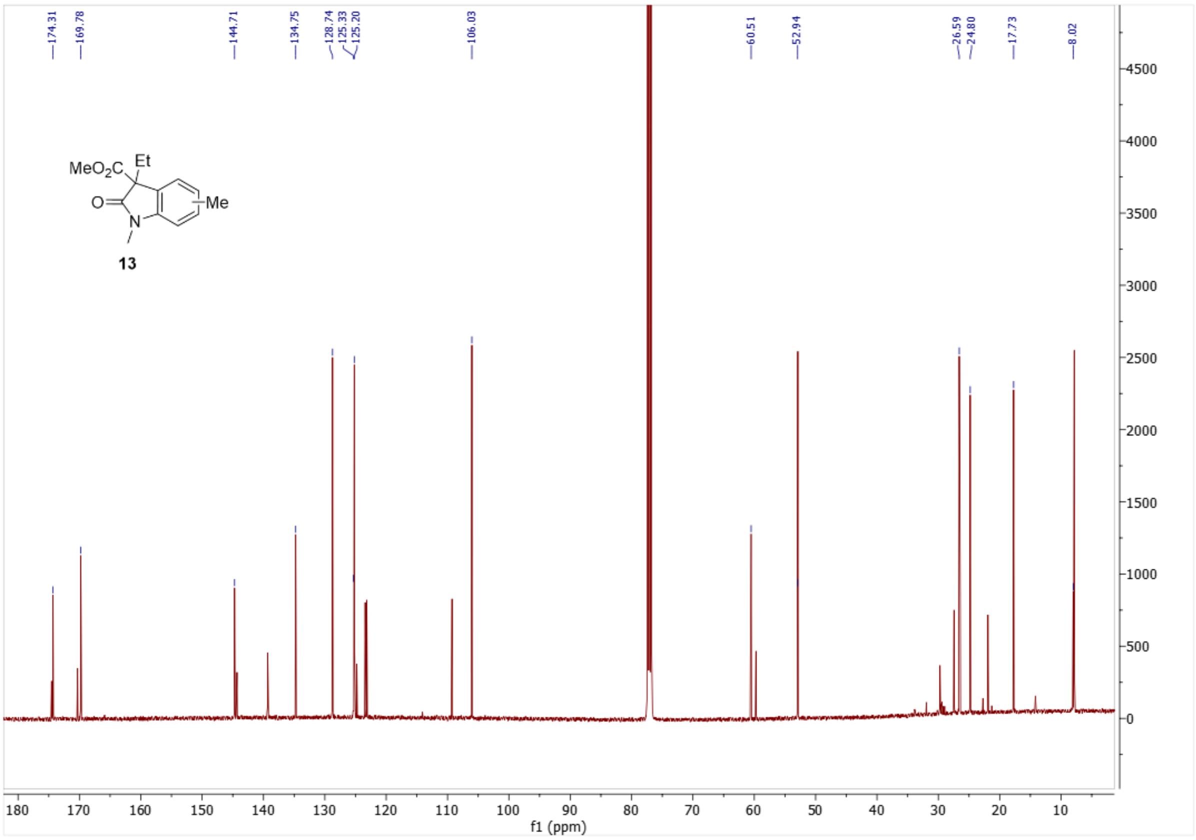

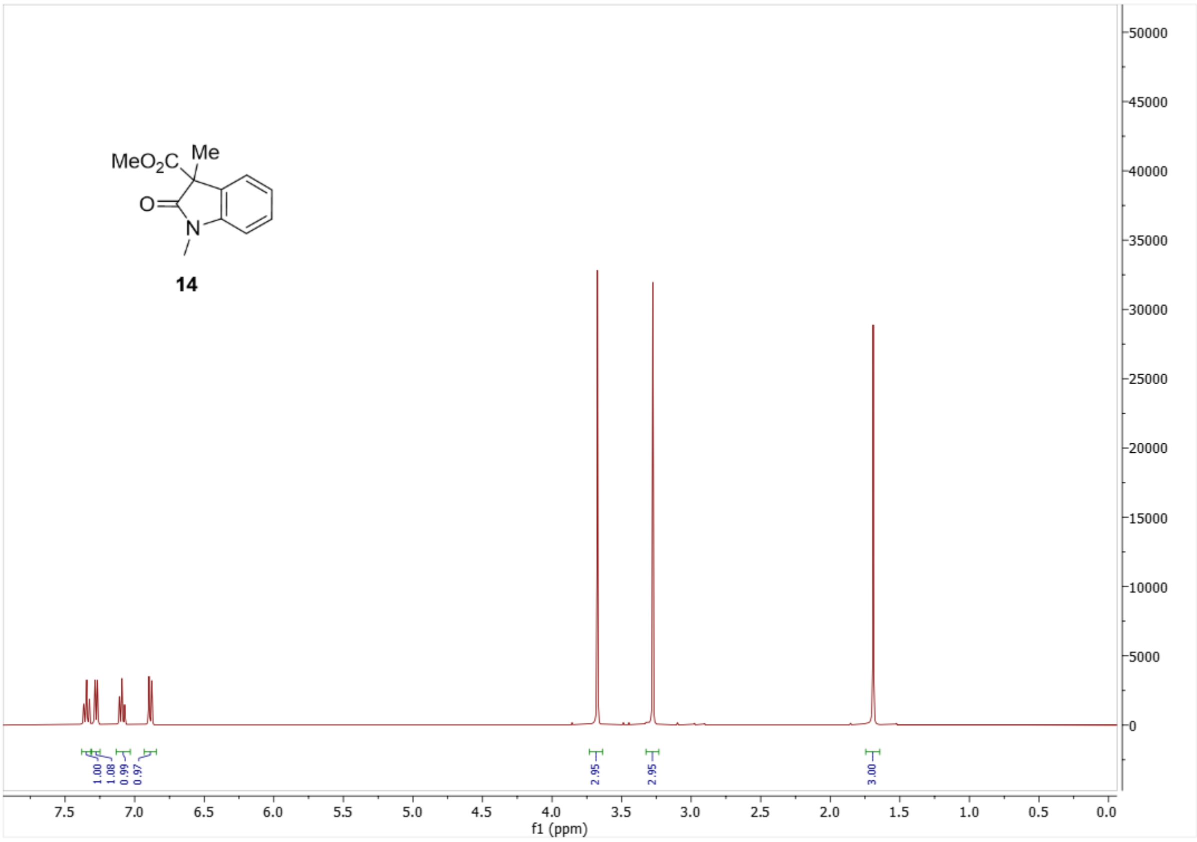
MeO2C
Me
O
N
14
1.00
1.08
0.99
0.97
2.95
2.95
3.00
f1 (ppm)

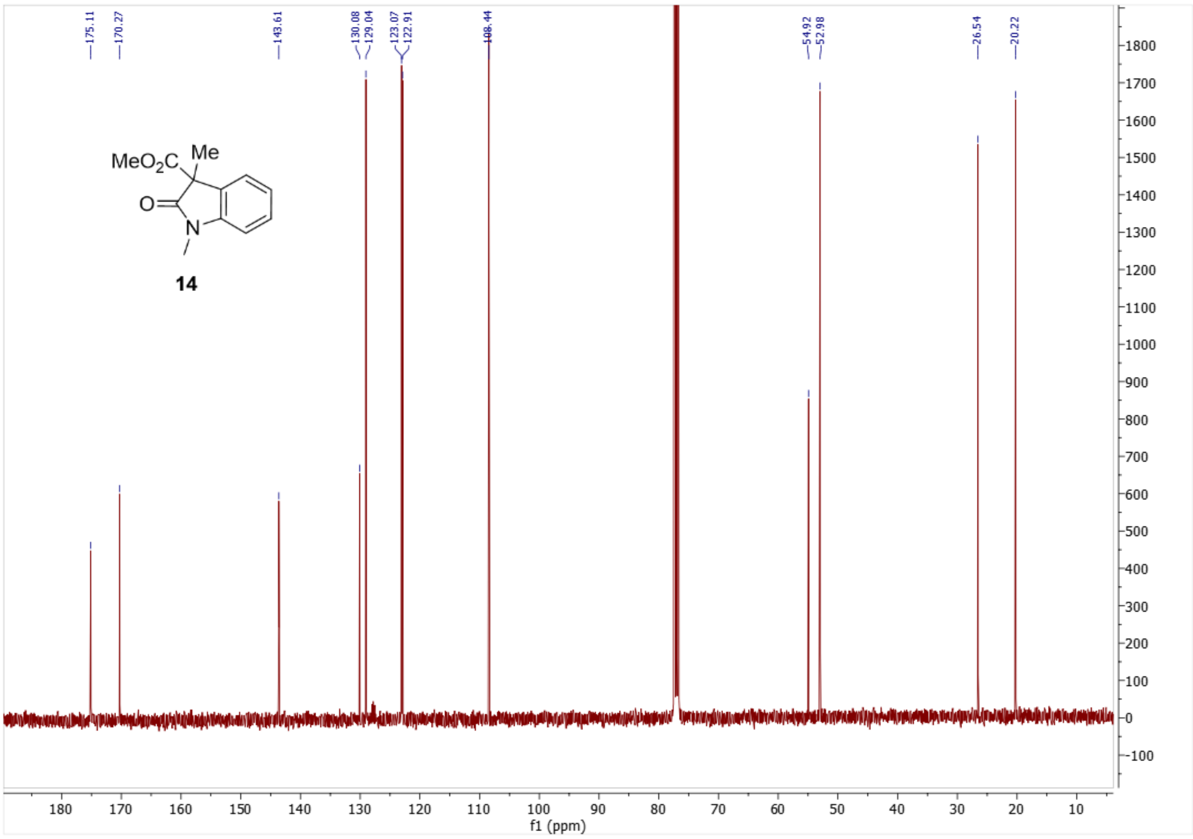
175.11
170.27
143.61
130.08
129.04
123.07
122.91
108.44
54.92
52.98
26.54
20.22
MeO2C
Me
O
N
14
f1 (ppm)

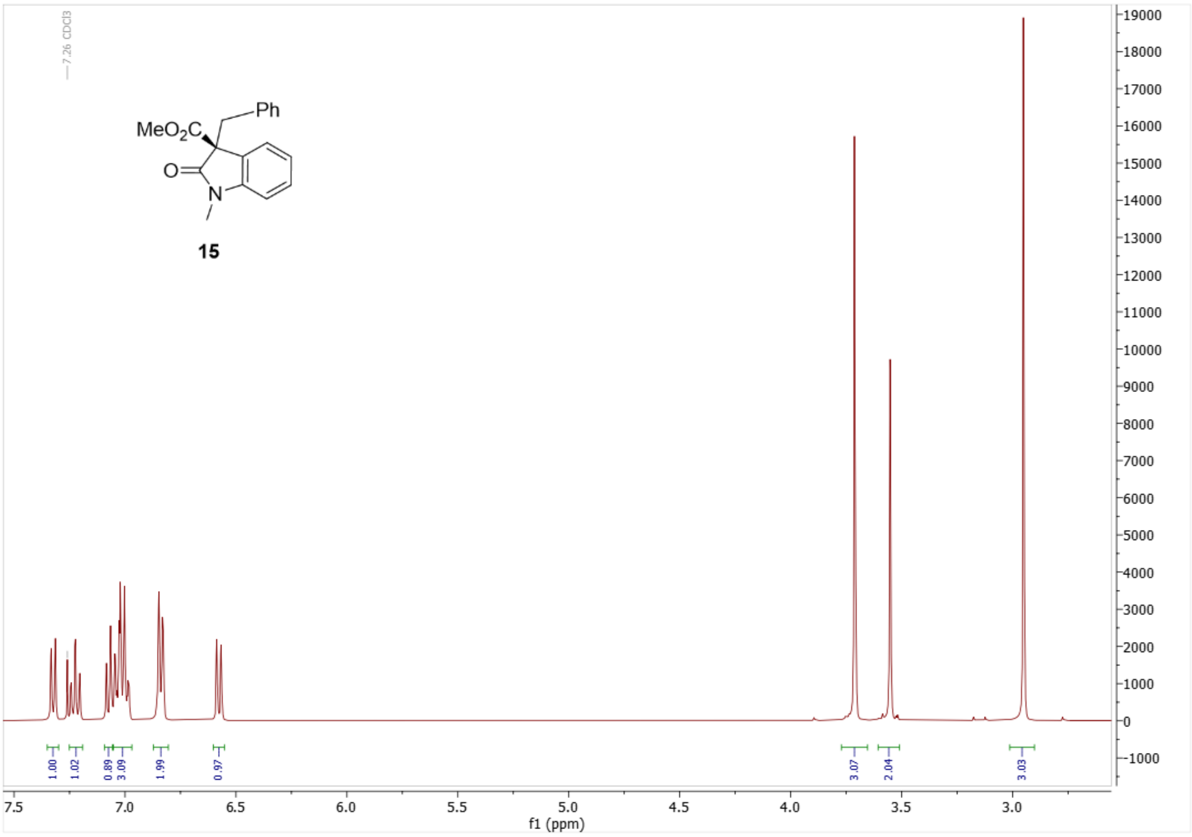
7.26 CDCl3
MeO2C
Ph
O
N
15
1.00
1.02
0.89
3.09
1.99
0.97
3.07
2.04
3.03
f1 (ppm)

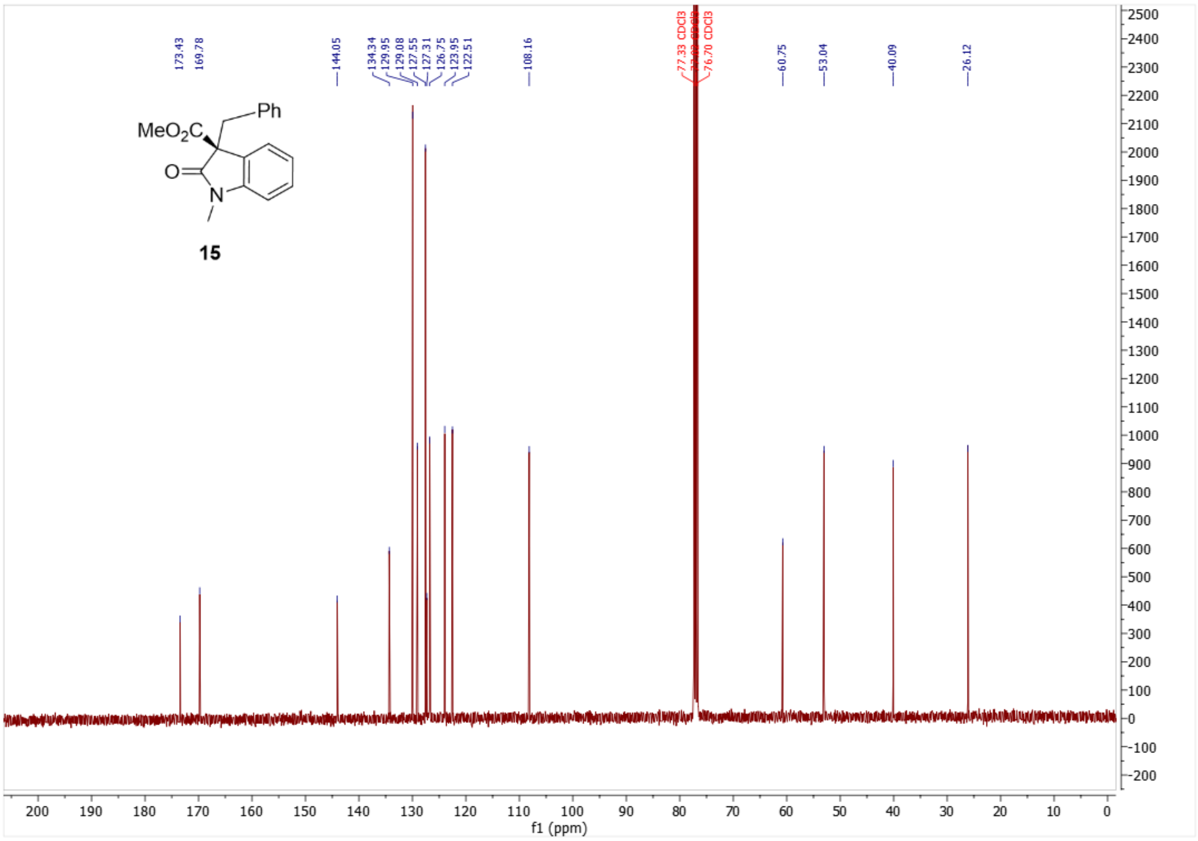
173.43
169.78
144.05
134.34
129.95
129.08
127.55
127.31
126.75
123.95
122.51
108.16
77.33 CDCl3
76.70 CDCl3
60.75
53.04
40.09
26.12
MeO2C
Ph
O
N
15
f1 (ppm)

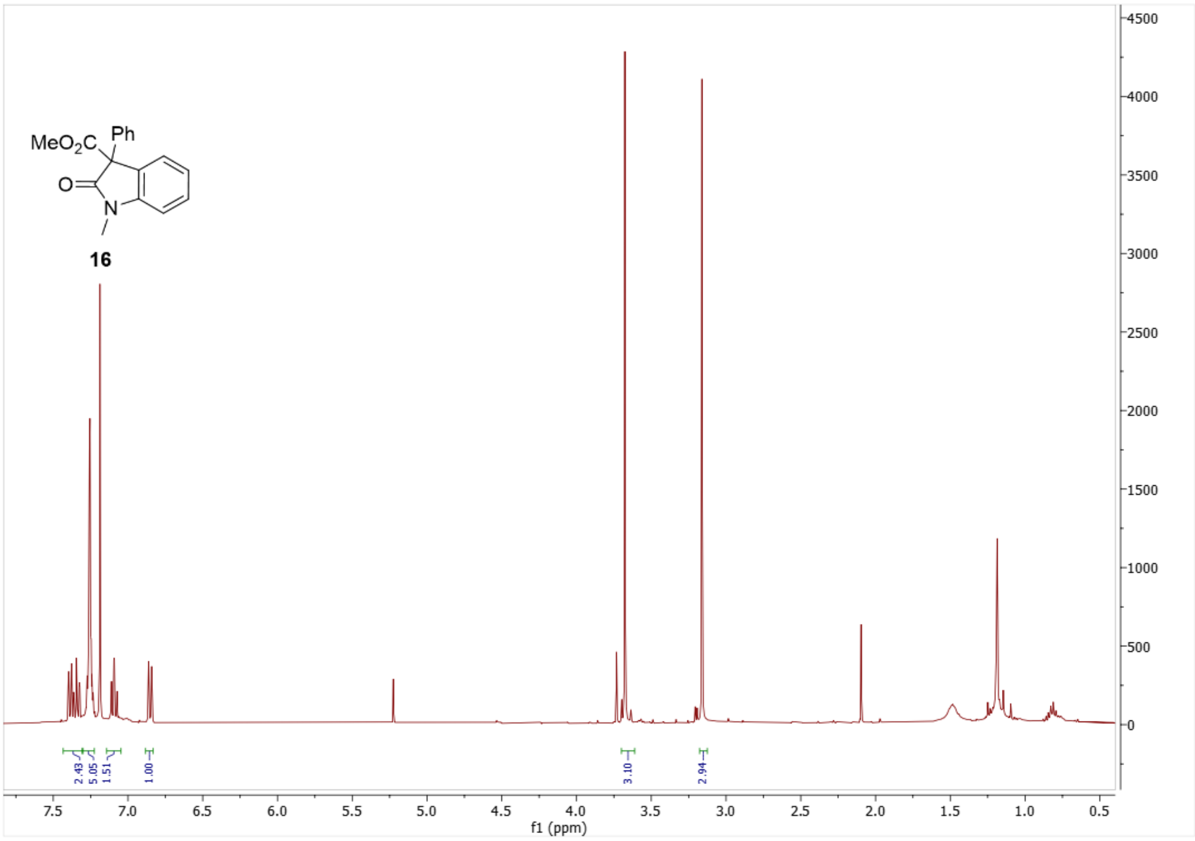
MeO2C
Ph
O
N
16
2.43
5.05
1.51
1.00
3.10
2.94
f1 (ppm)

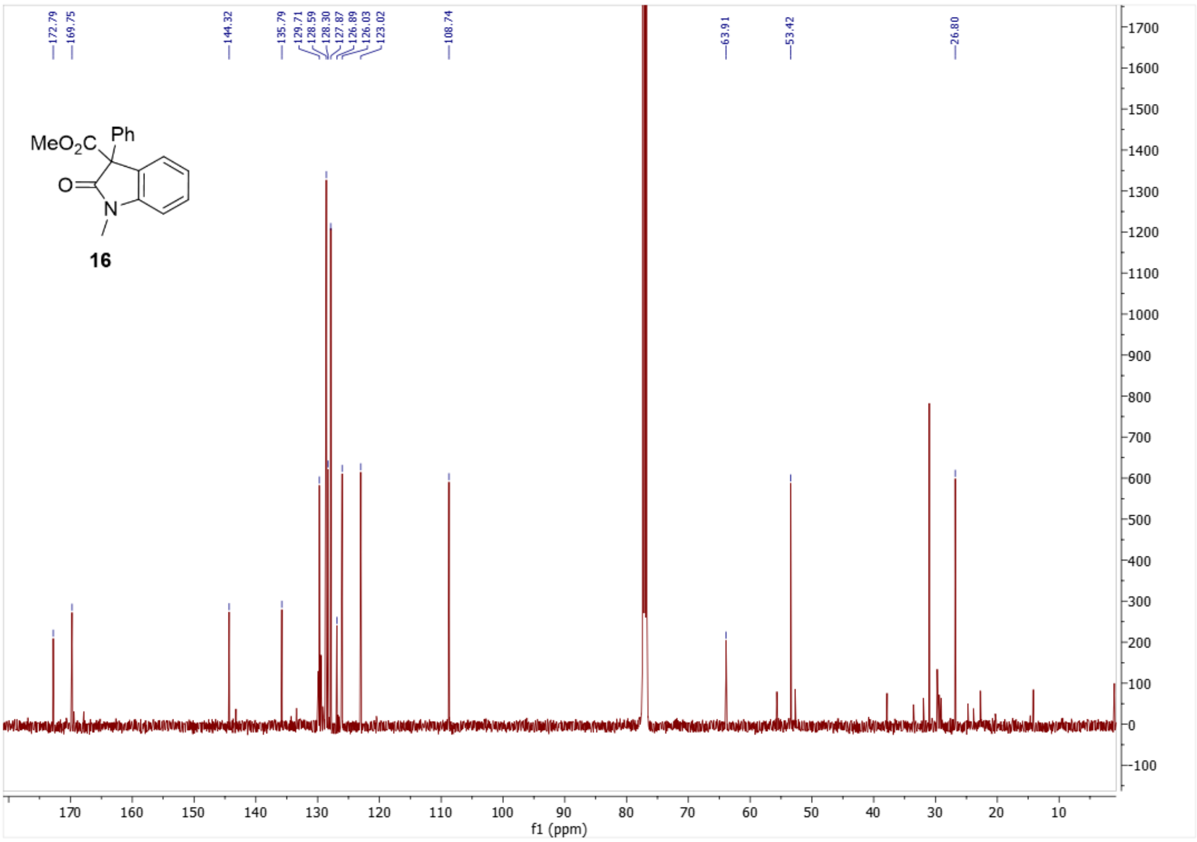
172.79
169.75
144.32
135.79
129.71
128.59
128.30
127.87
126.89
126.03
123.02
108.74
63.91
53.42
26.80
MeO2C
Ph
O
N
16
f1 (ppm)

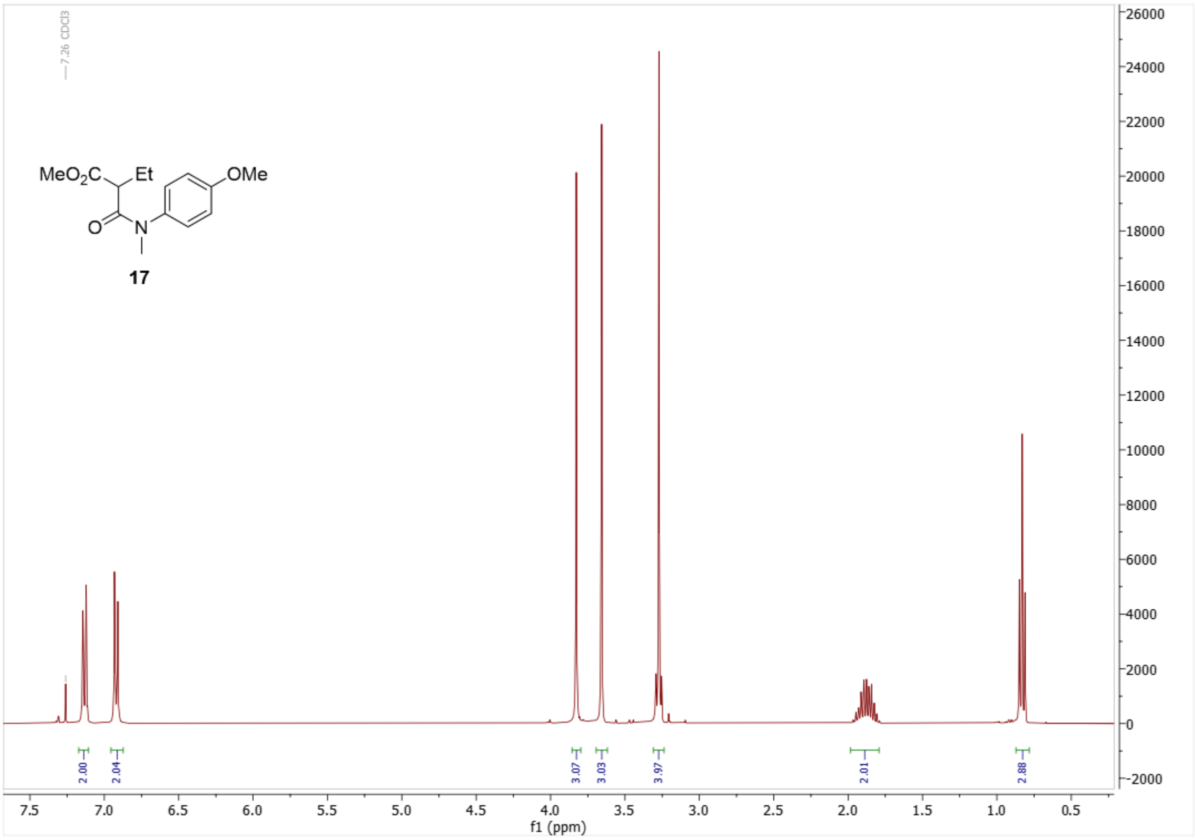
7.26 CDCl3
MeO2C
Et
OMe
O
N
17
2.00
2.04
3.07
3.03
3.97
2.01
2.88
f1 (ppm)

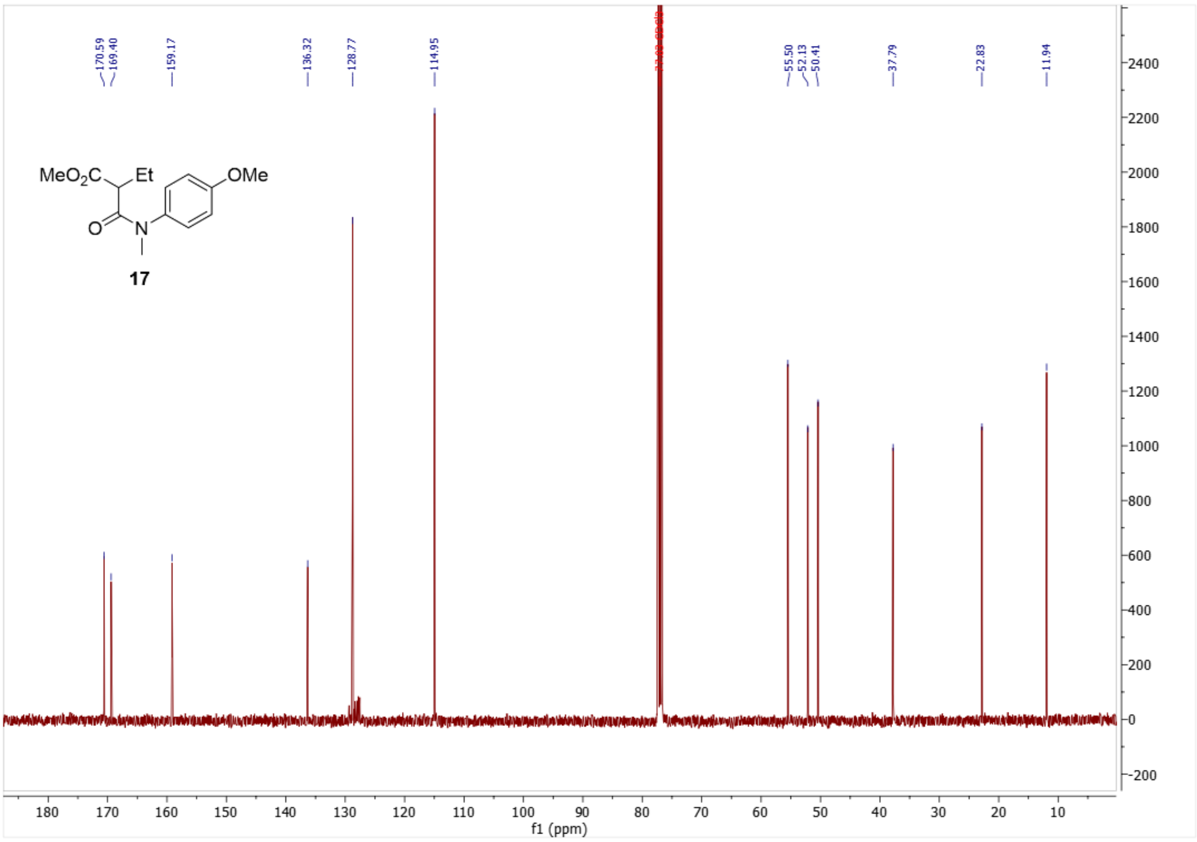
170.59
169.40
159.17
136.32
128.77
114.95
55.50
52.13
50.41
37.79
22.83
11.94
MeO2C
Et
OMe
O
N
17
f1 (ppm)

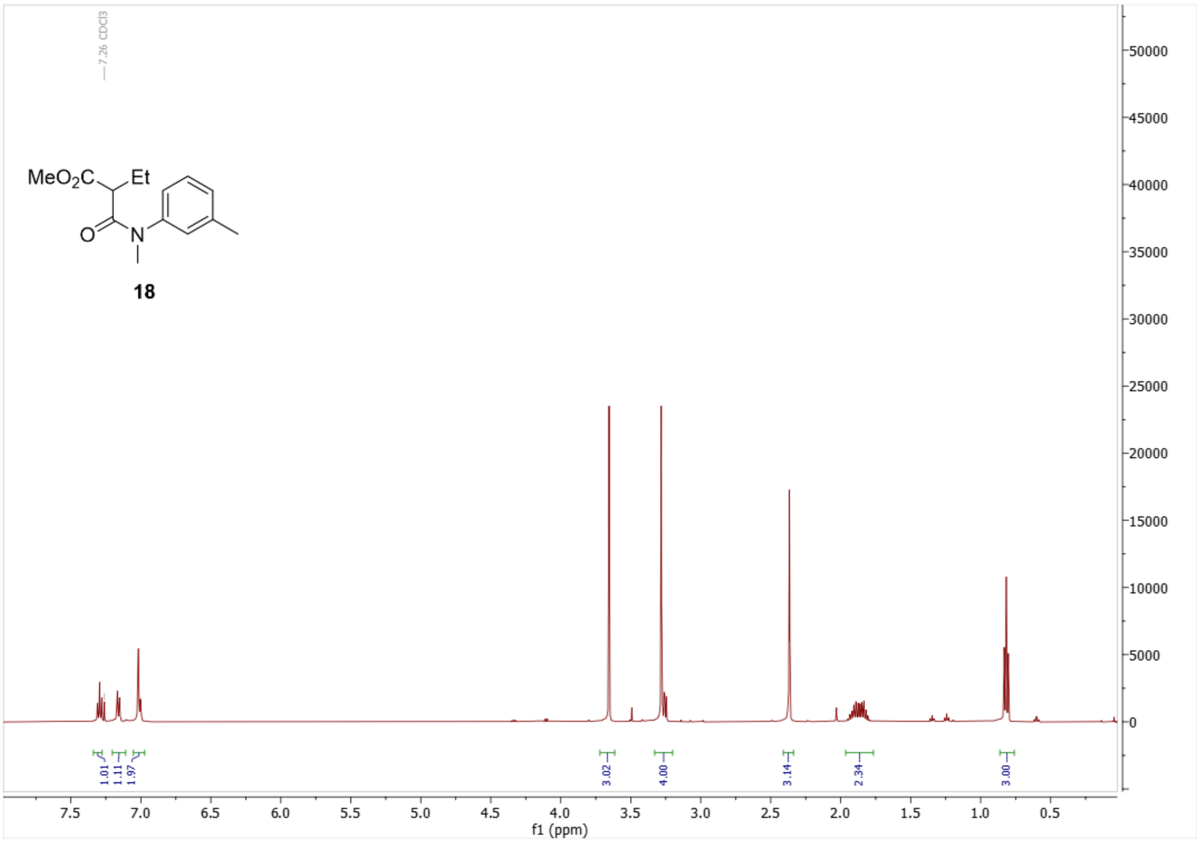

7.26 CDCl3
MeO2C
Et
O
N
18
1.01
1.11
1.97
3.02
4.00
3.14
2.34
3.00
f1 (ppm)

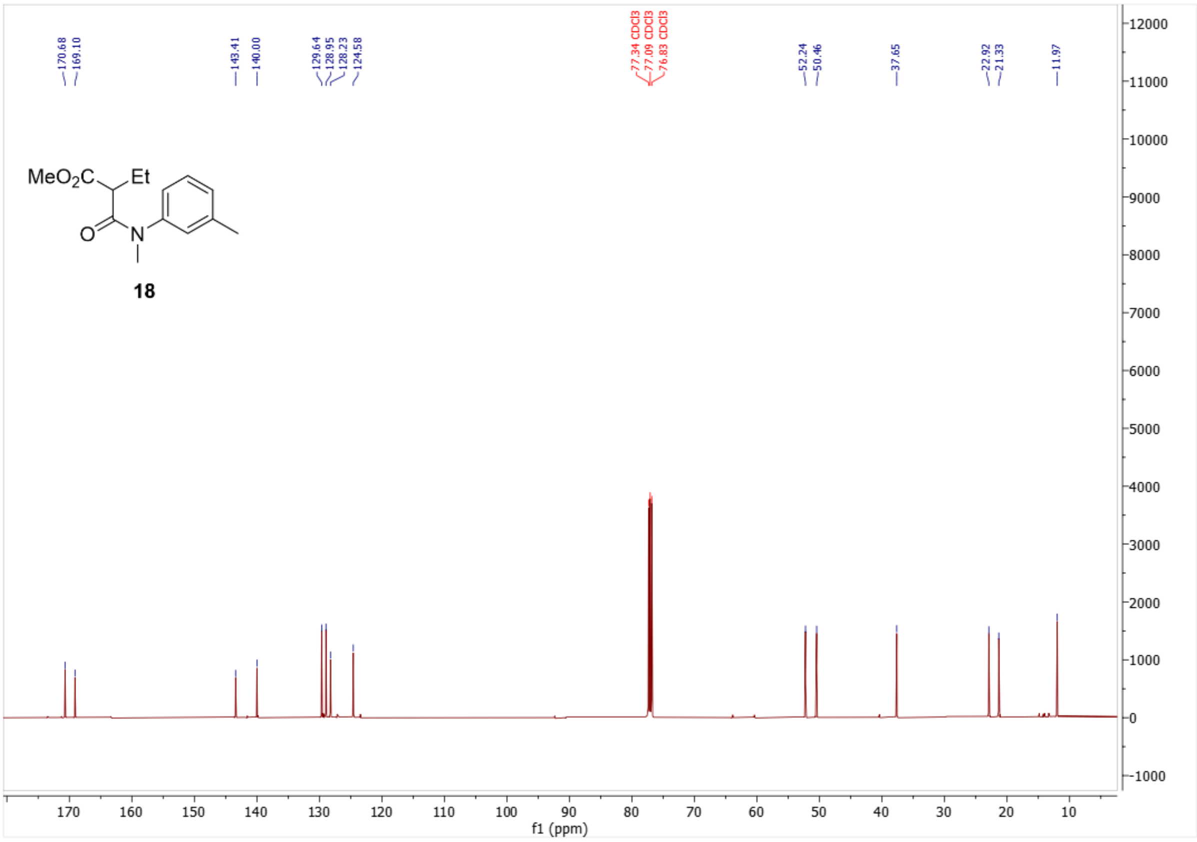

170.68
169.10
143.41
140.00
129.64
128.95
128.23
124.58
77.34 CDCl3
77.09 CDCl3
76.83 CDCl3
52.24
50.46
37.65
22.92
21.33
11.97
MeO2C
Et
O
N
18
f1 (ppm)

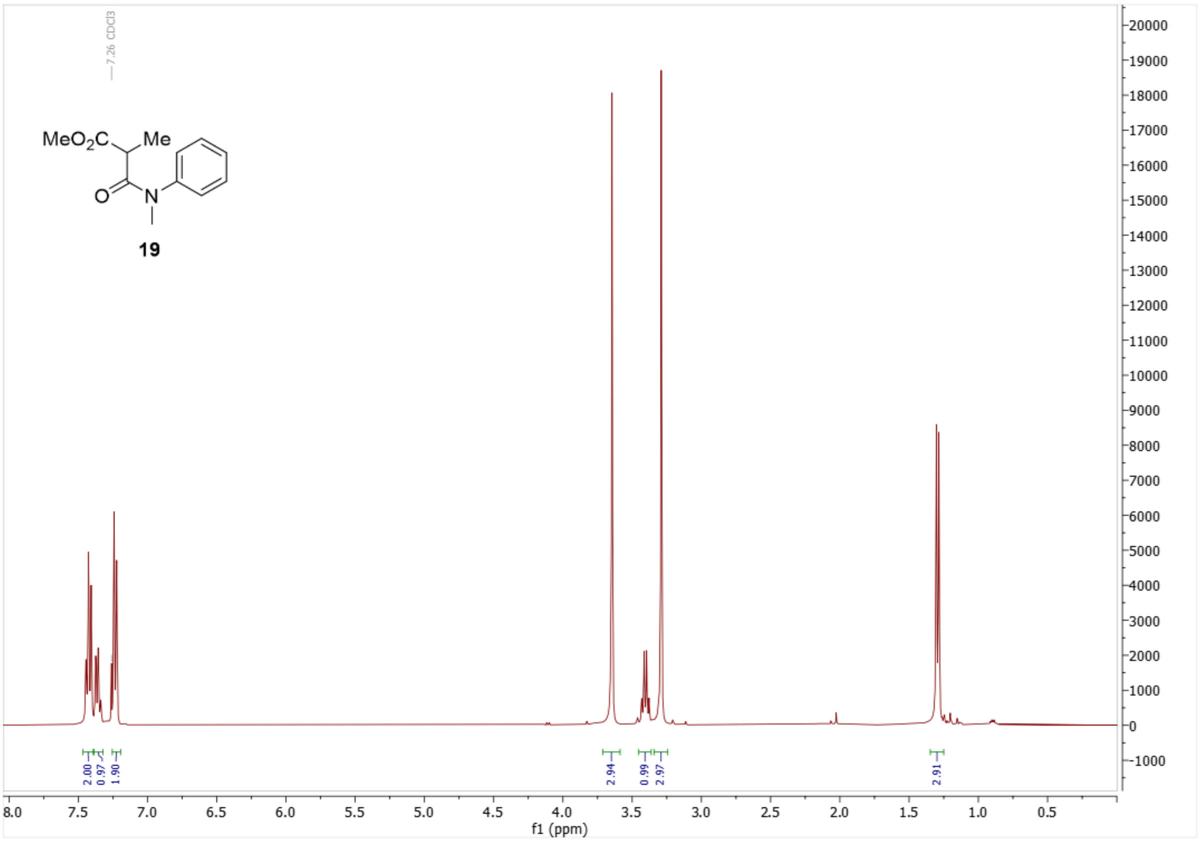
7.26 CDCl3
MeO2C
Me
O
N
19
2.00
0.97
1.90
2.94
0.99
2.97
2.91
f1 (ppm)

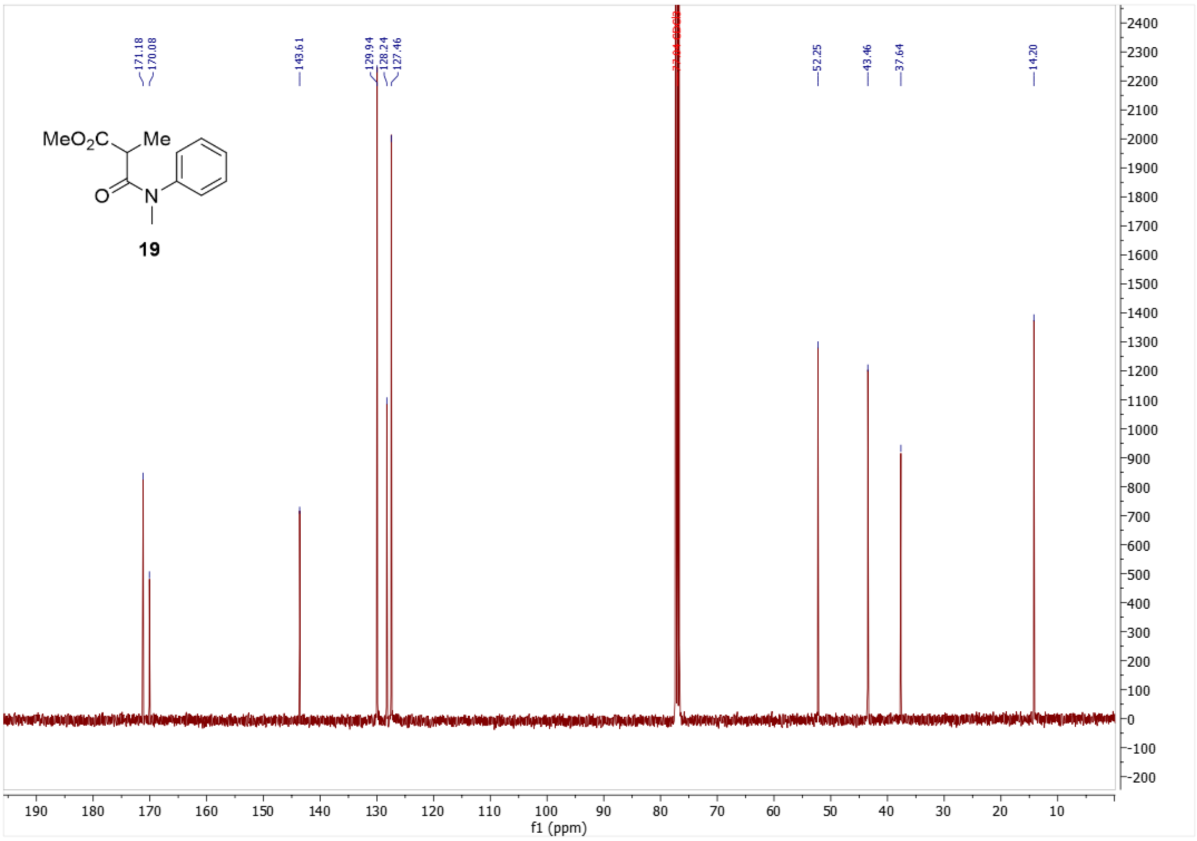
171.18
170.08
143.61
129.94
128.24
127.46
52.25
43.46
37.64
14.20
MeO2C
Me
O
N
19
f1 (ppm)

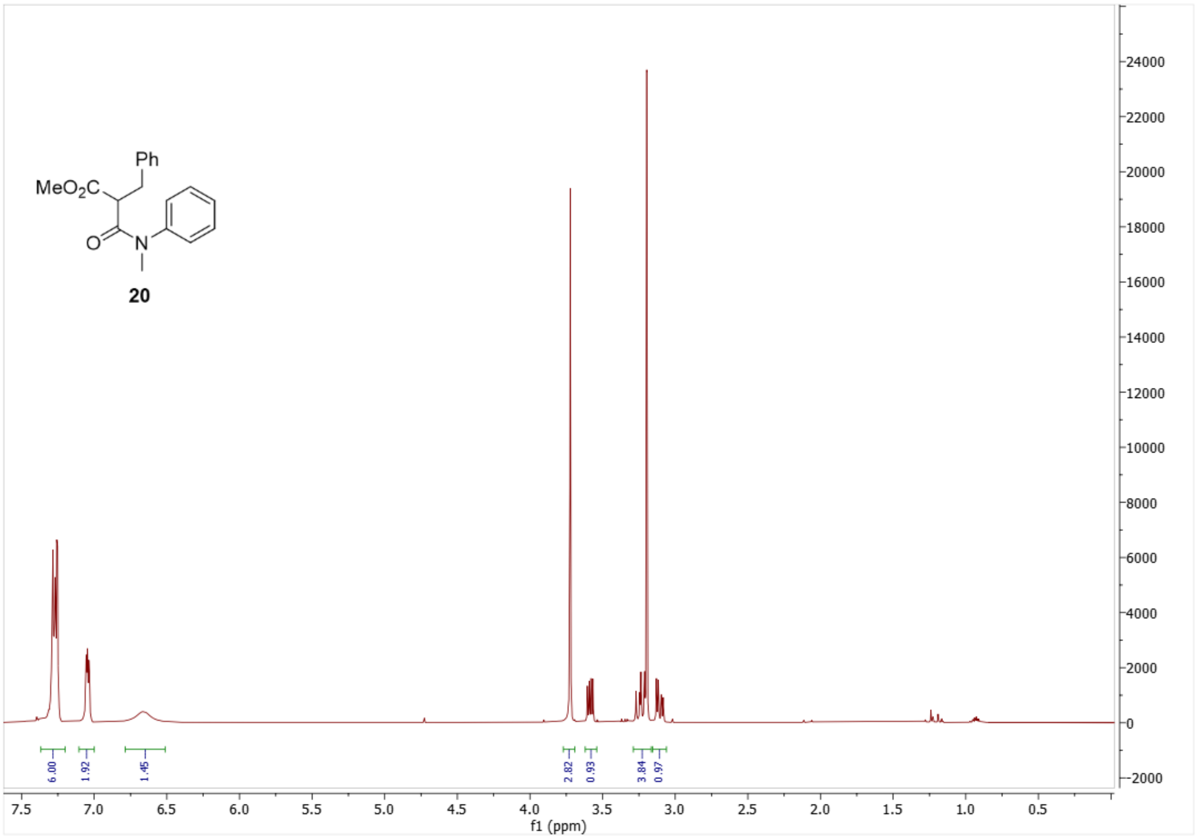

Ph
MeO2C
O
N
20
6.00
1.92
1.45
2.82
0.93
3.84
0.97
f1 (ppm)

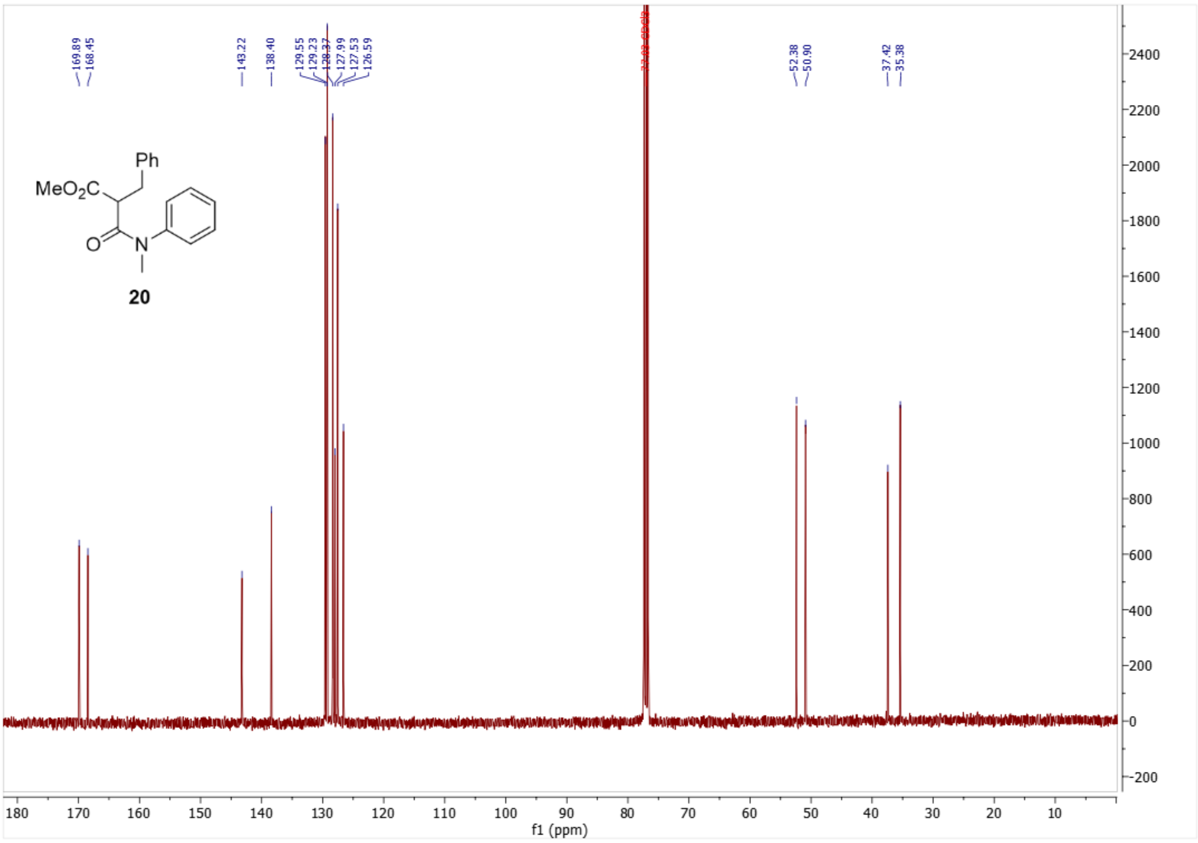

169.89
168.45
143.22
138.40
129.55
129.23
128.37
127.99
127.53
126.59
52.38
50.90
37.42
35.38
Ph
MeO2C
O
N
20
f1 (ppm)

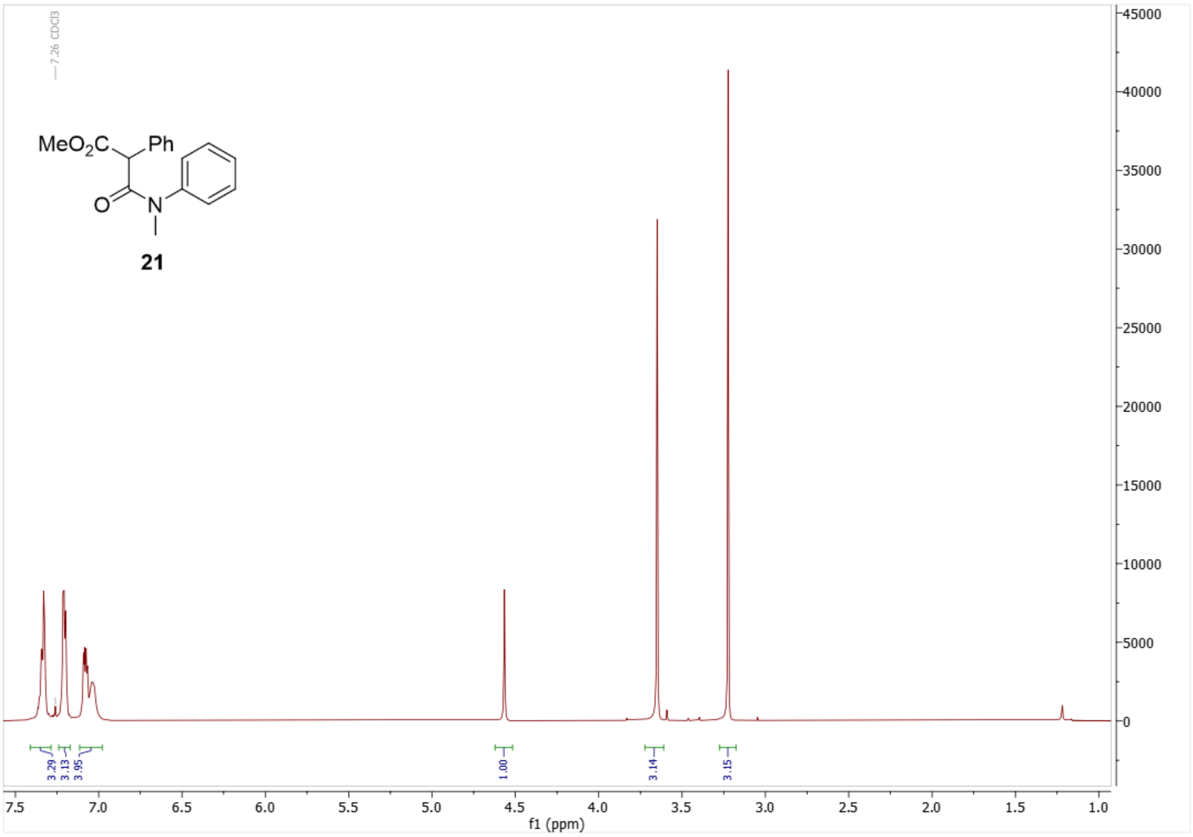

7.26 CDCl3
MeO2C
Ph
O
N
21
3.29
3.13
3.95
1.00
3.14
3.15
f1 (ppm)

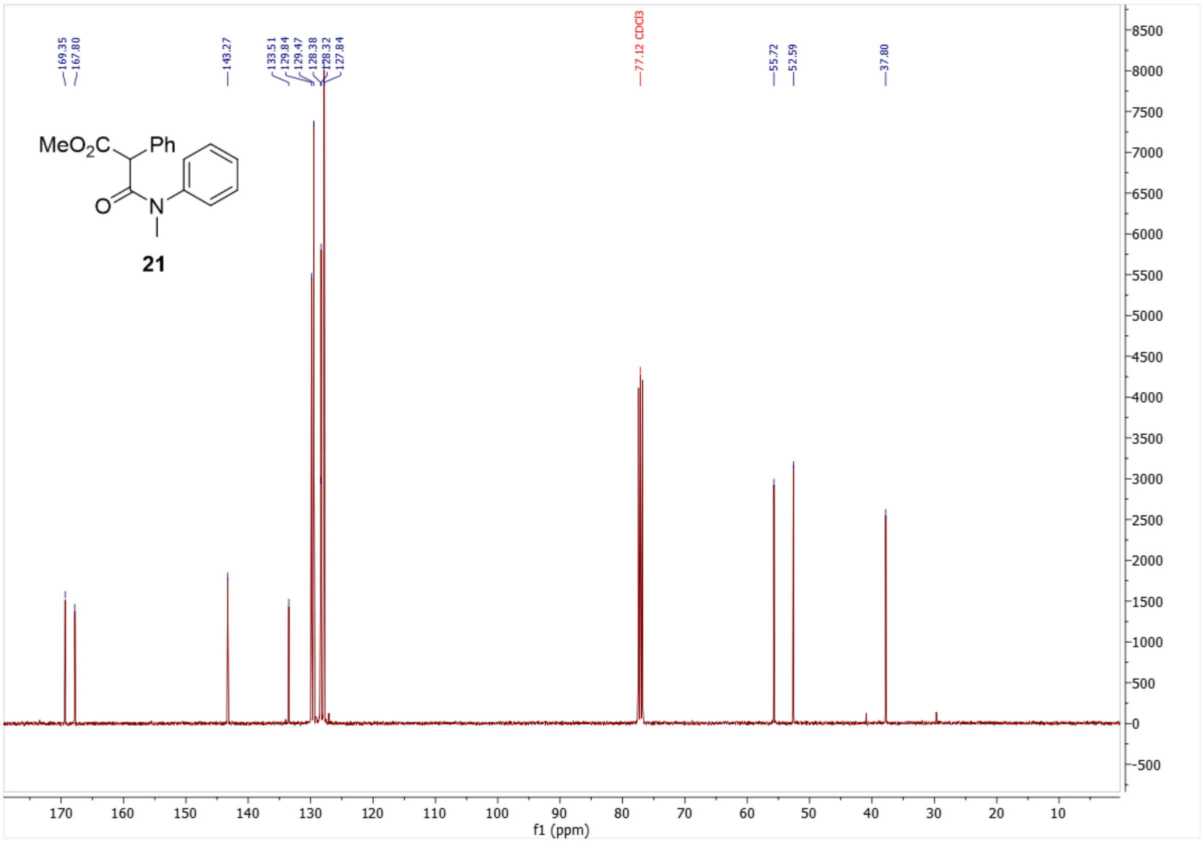

169.35
167.80
143.27
133.51
129.84
129.47
128.38
128.32
127.84
77.12 CDCl3
55.72
52.59
37.80
MeO2C
Ph
O
N
21
f1 (ppm)

S15 References

1. B. Jahn, N. S. W. Jonasson, H. Hu, H. Singer, A. Pol, N. M. Good, H. J. M. O. D. Camp, N. C. Martinez-Gomez, L. J. Daumann, *J. Biol. Inorg. Chem.* **2020**, *25*, 199–212.
2. J. A. Duine, J. F. Jzn, P. E. J. Verwiel, *Eur. J. Biochem.* **1980**, *108*, 187–192.
3. W. Dehaen, A. Hassner, *J. Org. Chem.* **1991**, *56*, 896–900.
4. J. Magolan, M. A. Kerr, *Org. Lett.* **2006**, *8*, 4561–4564.
5. J. W. Tucker, J. M. R. Narayanam, S. W. Krabbe, C. R. J. Stephenson, *Org. Lett.* **2009**, *12*, 368–371.
6. H. Abas, C. S. Frampton, A. C. Spivey, *J. Org. Chem.* **2016**, *81*, 9947–9956.
7. K. F. Biegasiewicz, S. J. Cooper, X. Gao, D. G. Oblinsky, J. H. Kim, S. E. Garfinkle, L. A. Joyce, B. A. Sandoval, G. D. Scholes, T. K. Hyster, *Science* **2019**, *364*, 1166–1169.
8. R. Shintani, M. Murakami, T. Hayashi, *J. Am. Chem. Soc.* **2007**, *129*, 12356–12357.
9. Niwayama, S. (2012). *U.S. Patent No. 8,338,635*. Washington, DC: U.S. Patent and Trademark Office.
10. J. Sun, G. Li, G. Zhang, Y. Cong, X. An, D. Zhang-Negrerie, Y. Du, *Adv. Synth. Catal.* **2018**, *360*, 2476–2481.
11. F. Zhou, Y. Liu, J. Zhou, *Adv. Synth. Catal.* **2010**, *352*, 1381–1407.
12. S. Itoh, H. Kawakami, S. Fukuzumi, *J. Am. Chem. Soc.* **1998**, *120*, 7271–7277.
13. Wohlwend, J.; Corso, G.; Passaro, S.; Reveiz, M.; Leidal, K.; Swiderski, W.; Portnoi, T.; Chinn, I.; Silterra, J.; Jaakkola, T.; Barzilay, R. Boltz-1 Democratizing Biomolecular Interaction Modeling. *bioRxiv* **2024**, https://doi.org/10.1101/2024.11.19.624167.
14. Gaussian 16, Revision C.01, M. J. Frisch, G. W. Trucks, H. B. Schlegel, G. E. Scuseria, M. A. Robb, J. R. Cheeseman, G. Scalmani, V. Barone, G. A. Petersson, H. Nakatsuji, X. Li, M. Caricato, A. V. Marenich, J. Bloino, B. G. Janesko, R. Gomperts, B. Mennucci, H. P. Hratchian, J. V. Ortiz, A. F. Izmaylov, J. L. Sonnenberg, D. Williams-Young, F. Ding, F. Lipparini, F. Egidi, J. Goings, B. Peng, A. Petrone, T. Henderson, D. Ranasinghe, V. G. Zakrzewski, J. Gao, N. Rega, G. Zheng, W. Liang, M. Hada, M. Ehara, K. Toyota, R. Fukuda, J. Hasegawa, M. Ishida, T. Nakajima, Y. Honda, O. Kitao, H. Nakai, T. Vreven, K. Throssell, J. A. Montgomery, Jr., J. E. Peralta, F. Ogliaro, M. J. Bearpark, J. J. Heyd, E. N. Brothers, K. N. Kudin, V. N. Staroverov, T. A. Keith, R. Kobayashi, J. Normand, K. Raghavachari, A. P. Rendell, J. C. Burant, S. S. Iyengar, J. Tomasi, M. Cossi, J. M. Millam, M. Klene, C. Adamo, R. Cammi, J. W. Ochterski, R. L. Martin, K. Morokuma, O. Farkas, J. B. Foresman, and D. J. Fox, Gaussian, Inc., Wallingford CT, 2016.
15. McNutt, A. T.; Francoeur, P.; Aggarwal, R.; Masuda, T.; Meli, R.; Ragoza, M.; Sunseri, J.; Koes, D. R. GNINA 1.0: molecular docking with deep learning. *J. Cheminf.* **2021**, *13* (1), 43.
16. M. J. Black, K. F. Biegasiewicz, A. J. Meichan, D. G. Oblinsky, B. Kudisch, G. D. Scholes, T. K. Hyster, *Nat. Chem.* **2019**, *12*, 71–75.
